# Electrophilic aromatic substitution using fluorinated isoxazolines at the C5 position *via* C–F bond cleavage†

**DOI:** 10.1039/d4ra07102f

**Published:** 2024-12-16

**Authors:** Kazuyuki Sato, Tomohiro Kuroki, Haruka Minami, Azusa Sato, Yukiko Karuo, Atsushi Tarui, Kentaro Kawai, Masaaki Omote

**Affiliations:** a Faculty of Pharmaceutical Sciences, Setsunan University 45-1 Nagaotoge-cho Hirakata Osaka 573-0101 Japan sato@pharm.setsunan.ac.jp; b School of Pharmacy, Tokyo University of Pharmacy and Life Sciences 1432-1 Horinouchi Hachioji Tokyo 192-0392 Japan

## Abstract

Electrophilic aromatic substitution at the C5 position of isoxazolines and construction of a new quaternary carbon center were achieved in this paper. This is the first report of carbon–carbon (C–C) bond formation onto isoxazoline without compromising the ring structure. Various aromatics including heteroaromatics gave the desired products in good yields, especially aromatics bearing electron-donating groups. The reaction proceeds *via* the S_E_Ar reaction mechanism, in which carbocation intermediates generated from the fluorinated isoxazolines *via* C–F bond cleavage reacted with aromatics.

## Abstract 2

Electrophilic aromatic substitution at the C5 position of isoxazolines and construction of a new quaternary carbon center were achieved in this paper.

## Graphical abstract

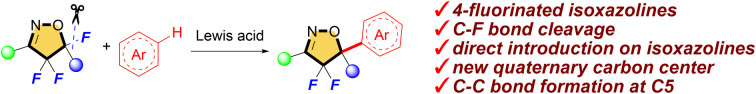

## Introduction

**Fig. 1:**
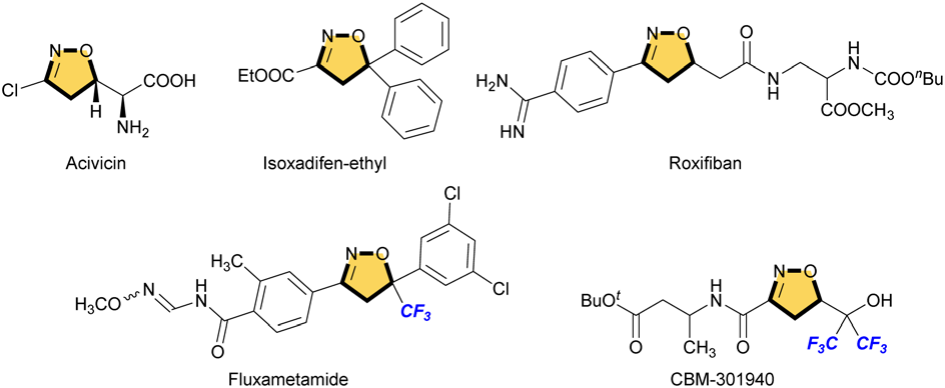
Isoxazolines in bio-active compounds.

Heterocycles are important frameworks that are widely distributed in nature. The isoxazoline pharmacophore is one of the most important classes of five-membered nitrogen–oxygen containing heterocyclic compounds.^1^ Acivicin is a fermentation product of *Streptomyces sviceus* and used as an effective inhibitor of γ-glutamyl transferase (Fig. 1).^2^ Isoxadifen-ethyl is used as a herbicide safener that minimizes the effect of the herbicide, and can effectively alleviate a sulfonylurea herbicide ‘nicosulfuron’ injury in maize.^3^ Roxifiban is a selective antagonist of the platelet glycoprotein IIb/IIIa receptor, which is the major receptor for fibrinogen on the platelet surface.^4^ Among such isoxazoline scaffolds, in particular, fluorinated or fluoroalkylated derivatives exhibit remarkable biological properties. For example, fluxametamide is an insecticide with a wide spectrum, and acts as an antagonist of GABA- and glutamate-gated chloride channels (GABA-Cl and Glu-Cl).^5^ Furthermore, CBM-301940 exhibited excellent *in vivo* PK/ADME properties and improved the cardiac efficiency in a rat heart global ischemia/reperfusion model.^6^

**Fig. 2:**
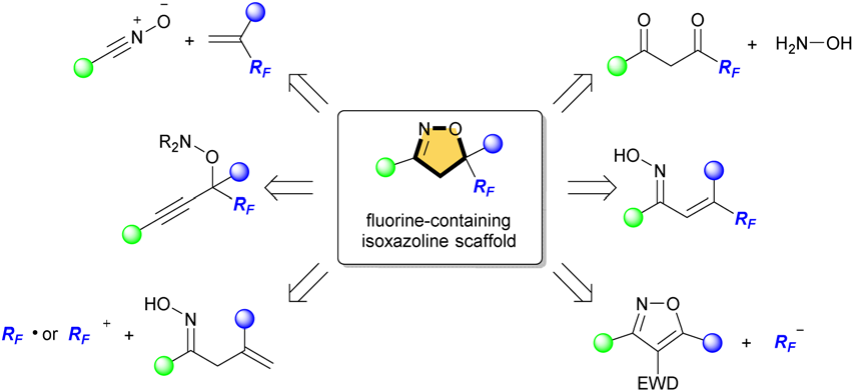
Synthetic methods for fluorine-containing isoxazolines.

**Scheme 1:**
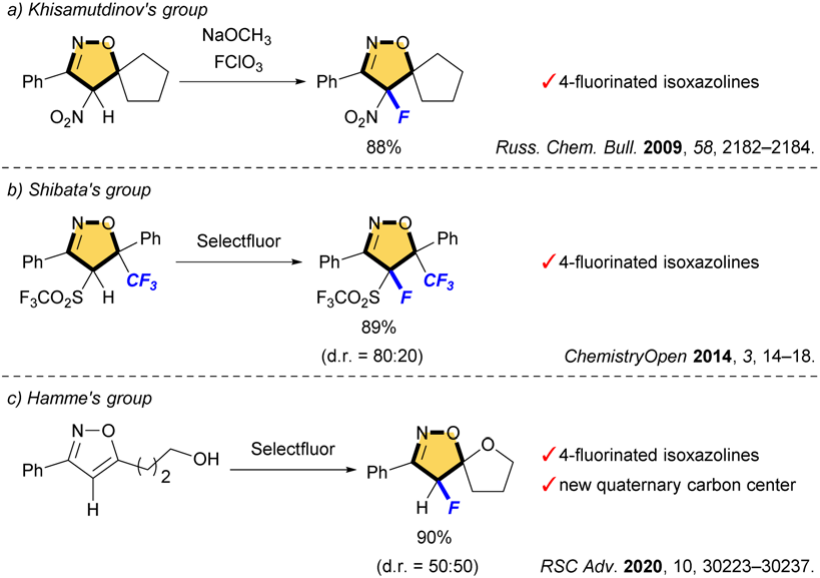
Synthesis of 4-fluorinated isoxazolines bearing a quaternary carbon at C5.

These scaffolds are commonly synthesized from fluorinated and/or fluoroalkylated building blocks which include classical reactions such as cycloaddition of nitrile oxide with alkene, condensation of diketone with hydroxylamine, and cyclization of oxime (Fig. 2).^7^ However, both 4-fluorinated and 4-fluoroalkylated isoxazolines scaffolds have been little synthesized, especially isoxazolines bearing a quaternary carbon center at C5 position, which is important for the expression of biological activity. The Khisamutdinov group and the Shibata group succeeded in fluorination of isoxazolines bearing electron-withdrawing group at C4, respectively.^8^ These reactions afforded the corresponding 4-fluorinated products in good yields, but the formation of a new quaternary carbon center was not achieved owing to the use of starting substances that already have substituents at the C5 position (Scheme 1a and b). In 2020, an interesting fluoro-spirocyclization of isoxazoles was reported by Hamme and his co-workers.^9^ They also succeeded in the synthesis of 4-fluorinated isoxazolines, and achieved the construction of a new quaternary carbon center at C5 *via* C–O bond formation as well (Scheme 1c). To the best of our knowledge, there are only three reports regarding fluorine-containing isoxazolines at C4 position, although Houk *et al.* also reported the [3 + 2] cycloaddition of nitrile oxides to give the related isoxazoline system, but those were not main products.^10^

On the other hand, a carbon–fluorine (C–F) bond is one of the most stable chemical bonds, because it has the highest bond dissociation energies.^11^ For this reason, transformations through stable C–F bond cleavage are difficult, but also challenging reactions. In recent years, several C–F bond cleavage reactions have been achieved by some groups, but there is still room in this field.^12^ Based on such important background, we are interested in the construction of fluorinated isoxazoline system bearing a quaternary carbon center at C5 by using stable C–F bonds cleavage aimed at synthesizing a wide range of functional fluorinated 5-membered heterocyclic systems.

**Scheme 2:**
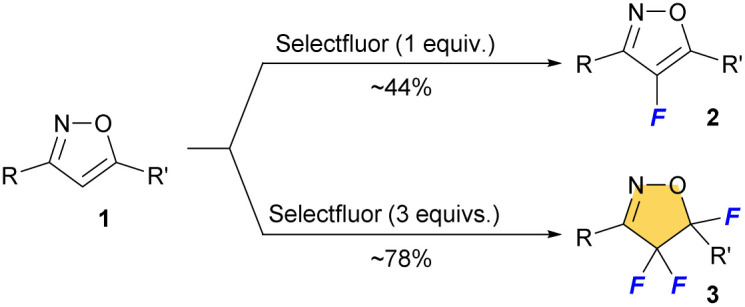
One-pot synthesis and selective fluorination of isoxazoles.

**Scheme 3:**
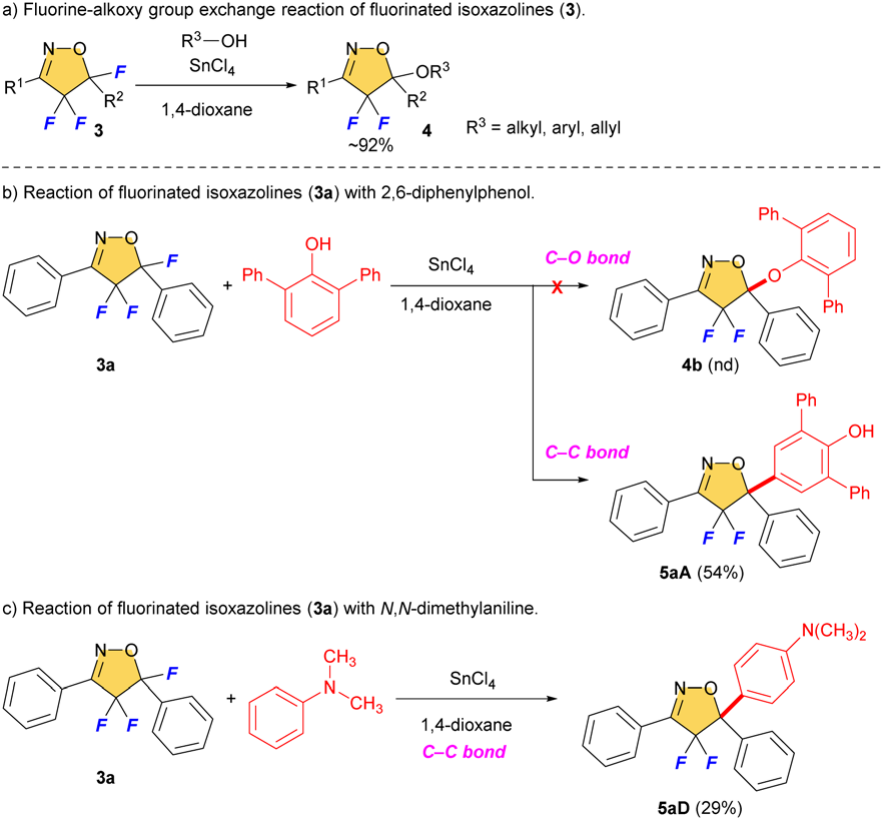
Fluorine-alkoxy group exchange reaction of fluorinated isoxazolines and unexpected S_E_Ar products.

**Scheme 4:**
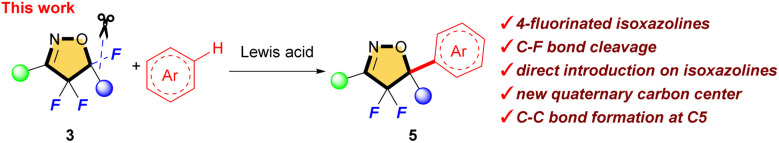
Direct introduction of carbon nucleophiles on fluorinated isoxazolines *via* C–F bond cleavage.

We reported selective fluorination of isoxazoles (1), which gave 4-fluorinated isoxazoles (2) or 4,4,5-trifluorinated isoxazolines (3) by using different amounts of Selectfluor, respectively (Scheme 2).^13^ Furthermore, when isoxazolines 3 and various alcohols were treated with SnCl_4_, the corresponding 5-alkoxylated products 4 were obtained in moderate to good yields (Scheme 3a).^14^ This reaction proceeds *via* an S_N_1 type process along with C–F bond cleavage, and then C–O bond formation by alcohol formed new quaternary carbon center at C5 position. In addition, this reaction could apply to other hetero atom nucleophiles such as thiols or amines to generate a new C–S or C–N bond at C5 position on starting isoxazolines.^15^ During the process of the synthesis of 4, sterically demanding phenol such as 2,6-diphenylphenol gave a novel aryl substituted product 5aA*via* electrophilic aromatic substitution (S_E_Ar) as shown in Scheme 3b.^14^ Furthermore, the reaction with *N*,*N*-dimethylaniline gave the similar C–C bond forming product 5aD that was introduced the aromatic ring directly at the C5 position of the isoxazoline scaffold, although the yield should be improved as shown in Scheme 3c. This is the first report for introducing carbon nucleophiles directly at the C5 position of the isoxazoline ring without compromising ring structure. In view of the results, we made the following hypothesis that the reaction of 3 with aromatic compounds might give various 4,4-difluoro-5-arylated isoxazolines (5) *via* S_E_Ar type processes which was directly constructed C–C bond at C5 position of isoxazolines (Scheme 4).

## Results and discussion

For introducing an aromatic ring at the C5 position of isoxazoline 3a, we investigated the reaction conditions by using toluene as a carbon nucleophile. According to the previous result, we applied the best condition of the fluorine-alkoxy group exchange reaction as shown in entry 1 (Table 1).^14^ However, compound 5aB was not obtained, but the main product is 5-hydroxylated compound 4a in 79%. Therefore, various reaction solvents were examined. As shown in entries 4 and 6, acetonitrile and sulfolane gave the desired product 5aB, and we found that sulfolane is the best solvent in this reaction. Next, various Lewis acids were examined. As shown in entry 7, using LiCl did not give the product at all, and only the starting material 3a was recovered. On the other hand, using other Lewis acids such as YbCl_3_, FeCl_3_, SnCl_2_ or TiCl_4_ gave 5-hydroxylated product 4a, although the 3a was consumed as shown in entries 9–12. Based on these examinations, BF_3_·Et_2_O was the best Lewis acid and it gave the desired product 5aB in 58% (entry 8). In further optimization of the reaction conditions, the solution concentrations and the temperature were investigated. In entry 13, increasing the solution concentration improved the yield of 5aB. However, further high concentration or high reaction temperature greatly decreased the yield as shown in entries 14 and 15. On the other hand, the shortened reaction time did not affect the reaction yield (entry 16). So, we decided entry 16 is the best condition to give the desired product 5aB.

**Table 1:** Reaction conditions for S_E_Ar using toluene

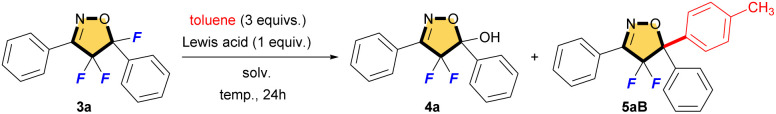						
Entry	Lewis acid	Solv. (mol L^−1^)	Temp (°C)	Yield^a^ (%)		
				3a	4a	5aB
1	SnCl_4_	14-Dioxane (0.125)	Reflux	Trace	79	Trace
2	SnCl_4_	DCE (0.125)	Reflux	(34)^b^	47	—
3	SnCl_4_	THE (0.125)	Reflux	(23)^b^	(20)^b^	—
4	SnCl_4_	CH_3_CN (0.125)	Reflux	—	57	18
5	SnCl_4_	DMF (0.125)	90	82	Trace	—
6	SnCl_4_	Sulfolane (0.125)	90	—	Trace	37
7	LiCl	Sulfolane (0.125)	90	96	—	—
8	BF_3_·Et_2_O	Sulfolane (0.125)	90	—	—	58
9	TiCl_4_	Sulfolane (0.125)	90	—	19	—
10	FeC1_3_·6H_2_O	Sulfolane (0.125)	90	—	61	—
11	SnCl_2_	Sulfolane (0.125)	90	—	98	—
12	YbC1_3_·6H_2_O	Sulfolane (0.125)	90	—	94	—
13	BF_3_·Et_2_O	Sulfolane (0.125)	90	—	—	66
14	BF_3_·Et_2_O	Sulfolane (0.5)	90	—	—	27
15	BF_3_·Et_2_O	Sulfolane (0.125)	120	—	—	12
16^c^	BF_3_·Et_2_O	Sulfolane (0.125)	90	—	—	64

a Isolated yield.

b The yield in parentheses was calculated by ^19^F NMR using PhCF_3_ as an internal standard.

c The reaction was carried out for 1 h.

On the basis of the optimized conditions, we explored the scope for this reaction, and the results are summarized in Table 2. The aromatics that are bearing on electron-donating groups proceeded smoothly to give the corresponding compounds (5aB–5aD, 5aH and 5aI) in moderate to good yields. On the other hand, the electron-deficient aromatics such as bromobenzene, benzotrifluoride and acetophenone did not work well, but only 5-hydroxylated product 4a was identified on ^19^F NMR. Heteroaromatic compounds could be applied to this reaction. Using *N*-methylpyrrole as the substrate, the desired compound 5aJ was obtained in 43% together with its regioisomer 5aJ′ in 43% yield. Fortunately, these isomers (5aJ and 5aJ′) were separable by column chromatography and the total yield of this reaction was 86%. With furan and thiophene, the reaction gave rise to the corresponding S_E_Ar products (5aK and 5aL) including a small amount of their regioisomers in 54% and 86% yields. However, pyridine did not give the product at all, but the only starting material 3a was recovered. Pyridine is a basic amine, so the formation of a BF_3_ salt might be predominated during the reaction. Isoxazolines having substituted aromatics and alkyl group also reacted with toluene to give the corresponding products (5bB, 5cB, and 5dB). It is interesting that 5cB involving chlorophenyl group at C5 could be obtained in a good yield by using the corresponding starting material, although the introduction of halobenzene *via* the S_E_Ar reaction failed (see 5aE). As a side note, the molecular structures of 5aH, 5aI and 5aJ were characterized by using single-crystal XRD analyses (5aH (CCDC: 2349494), 5aI (CCDC: 2349492) and 5aJ (CCDC: 2349493)).

**Table 2:** S_E_Ar reaction of 4,4,5-trifluorinated isoxazolines with aromatics *via* C–F bond cleavage

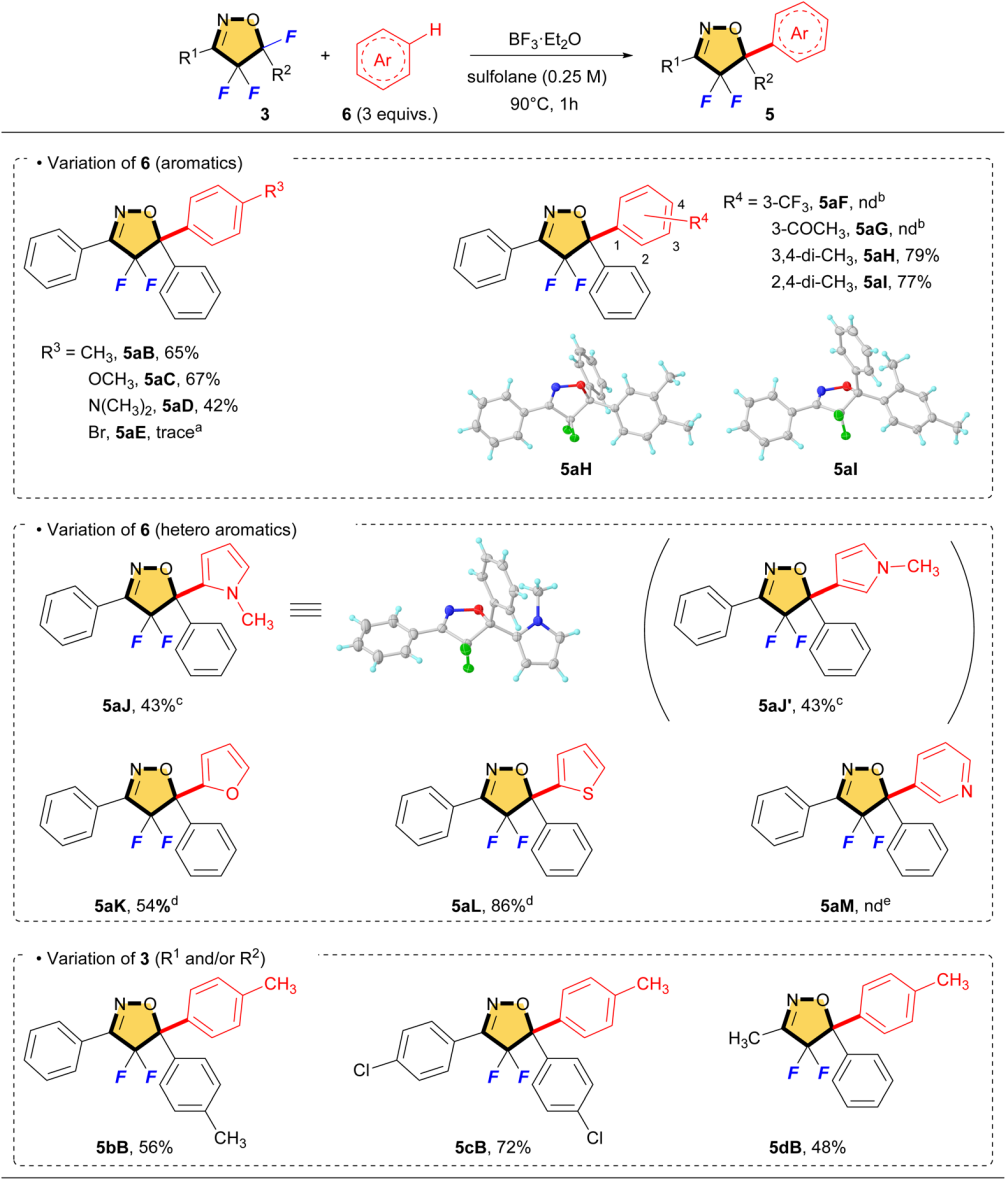

a The product and the starting material (3a) were identified on ^19^F NMR.

b Only the 5-hydroxylated product (4aA) was identified on ^19^F NMR.

c Separable regioisomer (5aJ′) was obtained in 43%.

d lnseparable regioisomer was obtained in a small amount.

e The starting material (3a) was recovered.

**Fig. 3:**
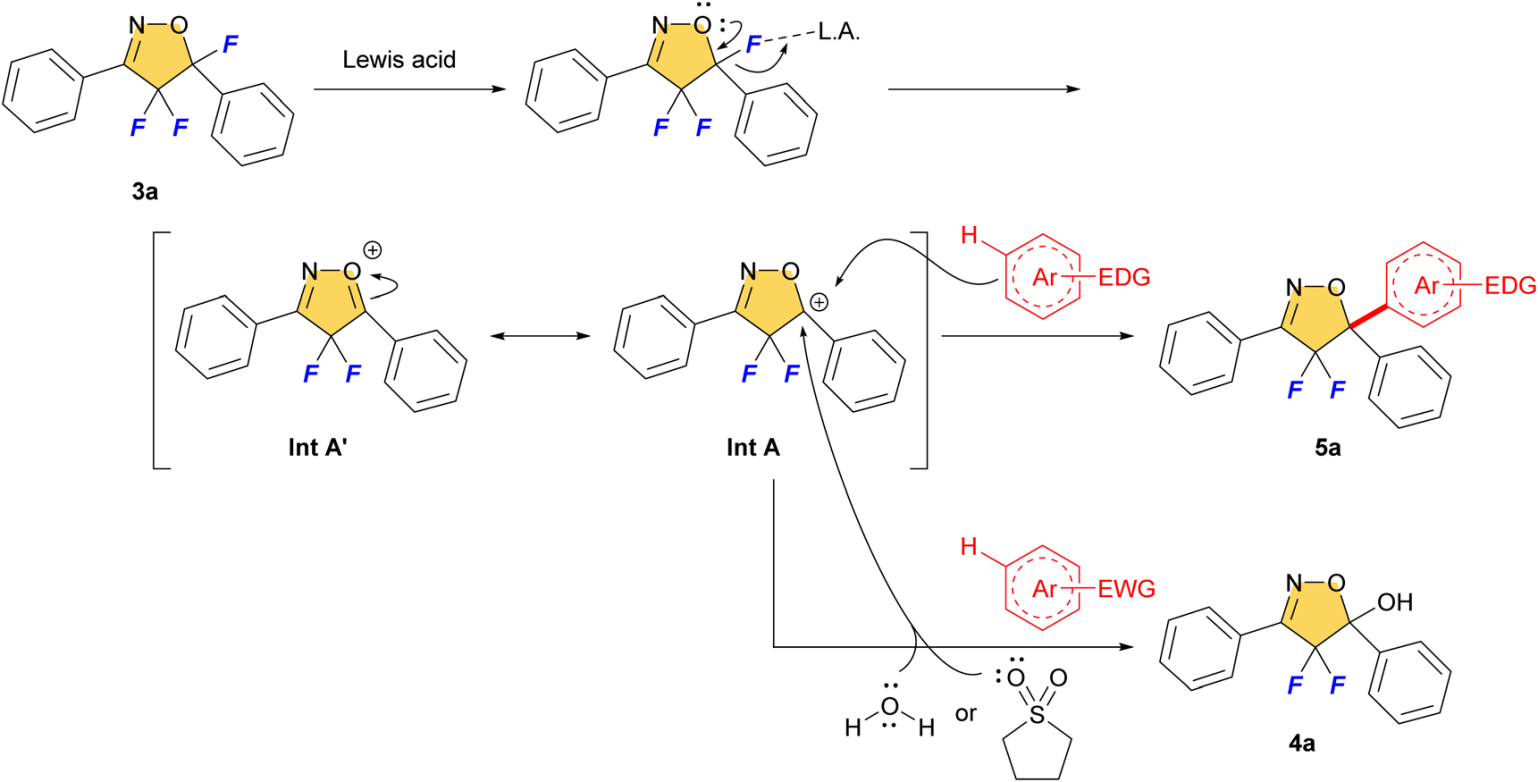
Proposed reaction mechanism of S_E_Ar reaction.

We envisage that the reaction proceeds *via* S_E_Ar reaction mechanism (Fig. 3). The C–F bond of 3a would be dissociated by Lewis acid and give the carbocation intermediate A (Int A). The stabilized Int A by the adjacent oxygen atom and/or benzene ring was trapped by the aromatics *via* S_E_Ar mechanism to give 5-arylated product 5 that bears a new quaternary carbon center. On the other hand, using poorly reactive electron-deficient aromatics preferentially led to the trapping of sulfolane and/or H_2_O towards Int A to give 5-hydroxylated product 4a reflecting the lower nucleophilicity of the aromatic substrate bearing an electron withdrawing substituent. So, it is important to use ‘dry’ solvent in this reaction.

**Scheme 5:**
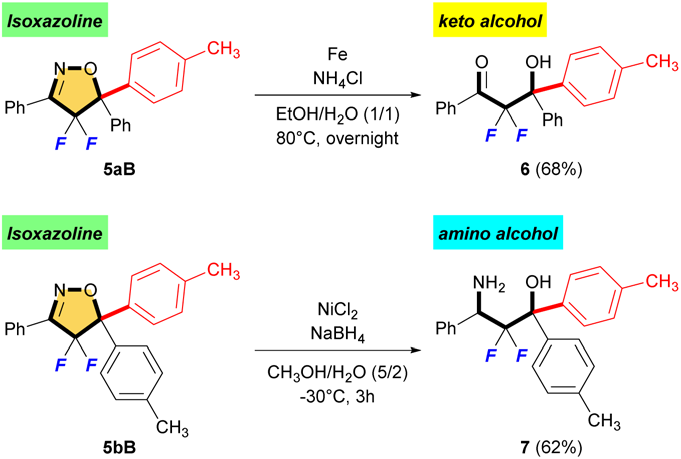
The synthetic utility of fluorinated isoxazolines.

In the last part, we explored several constructive reactions to demonstrate the synthetic utility of fluorinated isoxazoline products (Scheme 5). A reductive N–O bond cleavage of 5aB followed by hydrolysis of imine to give α,α-difluoro-β-hydroxy ketone 6 in 68% yield.^16^ Furthermore, treatment of 5bB under reductive ring-opening condition by NaBH_4_ and NiCl_2_ afforded the corresponding α,α-difluoro-β-amino alcohol 7 in 62% yield.^17^ Interestingly, these are the first synthetic examples of α,α-difluoro-β-hydroxy ketone and α,α-difluoro-β-amino alcohol bearing two aromatic rings on the quaternary carbon center, although many syntheses of similar compounds using Reformatsky-type reaction and/or aldol-type reaction have been reported.^18^

## Conclusions

In conclusion, we succeeded in introducing various aromatic substituents at the C5 position of the fluorinated isoxazolines. Using the aromatics bearing electron-donating groups and heteroaromatics gave the desired products 5 in good yields. On the other hand, electron deficient aromatics did not give the products. The reaction would proceed *via* the S_E_Ar reaction mechanism, in which carbocation intermediates generated from the fluorinated isoxazolines *via* stable C–F bond cleavage reacted with aromatics. Isoxazoline is an important framework for bioactive compounds, and we expect that these products also have interesting activities.

## Data availability

All experimental procedures and additional data can be found in the ESI.†

## Author contributions

KS wrote the manuscript. KS and MO conceived and designed the experiments. KS, TK and HM performed the experiments and analyzed the data. AS carried out the single-crystal XRD analyses. All authors discussed the results and reviewed the manuscript.

## Conflicts of interest

There are no conflicts to declare.

## Supplementary Material

RA-014-D4RA07102F-s001

RA-014-D4RA07102F-s002
