# Supplementary material for: Electrophilic aromatic substitution using fluorinated isoxazolines at the C5 position via C–F bond cleavage
Source: RSC Adv. 2024 Dec 16;14(53):39543–9. doi: 10.1039/d4ra07102f (PMC11647199; doi:10.1039/d4ra07102f)
Supplement: RA-014-D4RA07102F-s001 [file RA-014-D4RA07102F-s001.pdf]

Supplementary Information (SI) for RSC Advances.
This journal is © The Royal Society of Chemistry 2024

Electrophilic aromatic substitution using fluorinated isoxazolines at C5 position via C-F bond cleavage.

Kazuyuki Sato,*[1] Tomohiro Kuroki,[1] Haruka Minami,[1] Azusa Sato,[2] Yukiko Karuo,[1] Atsushi Tarui,[1] Kentaro Kawai,[1] Masaaki Omote*[1]

1) Faculty of Pharmaceutical Sciences, Setsunan University, 45-1 Nagaotoge-cho, Hirakata, Osaka, 573-0101, Japan

2) School of Pharmacy, Tokyo University of Pharmacy and Life Sciences, 1432-1 Horinouchi, Hachioji, Tokyo 192-0392, Japan

Electronic Supplementary Information (ESI)

General Information …………………………………………………. S2
Materials ……………………………………………….…………… S2
Experimental Section
    General procedure for the synthesis
        of 4,4,5-trifluoroisoxazolines. …………….. S2
    General procedure for S_EAr reaction
        with fluorinated isoxazolines. …………..… S2
    Typical procedure for the synthesis of **6**
        via reductive N-O bond cleavage. ……..… S3
    Typical procedure for the synthesis of **7**
        via reductive N-O bond cleavage. ……..… S3
Spectroscopic Data …………………………………………... S4–S9
References ……………………………………………………... S9
NMR charts ……………………………………….……. S10–S37
X-ray crystallographic data ………………………………. S38–S40

General Information:

^{1}H NMR, ^{13}C NMR and ^{19}F NMR spectra were recorded on a JEOL JNM-ECZS-400 spectrometer. Chemical shifts of ^{1}H NMR are reported in ppm from tetramethylsilane (TMS: 0 ppm) as an internal standard. Chemical shifts of ^{13}C NMR are reported in ppm from tetramethylsilane (TMS: 0 ppm) as an internal standard. Chemical shifts of ^{19}F NMR are reported in ppm from trichlorofluoromethane ($CFCl_3$: 0 ppm) as an internal standard. All data are reported as follows: chemical shifts, relative integration value, multiplicity (s = singlet, d = doublet, t = triplet, q = quartet, br = broad, m = multiplet), coupling constants (Hz). Mass spectra were obtained on JEOL JMS-700T spectrometers. Melting points were measured on Yanagimoto micro melting point apparatus MP-S3. Details of high quality and resolution X-ray diffraction experiments are shown in the section "X-ray crystallographic data"

Materials:

Sulfolane was distilled before use. Tetrahydrofuran (THF) was distilled over benzophenone ketyl sodium just before use. All commercially available reagents were used without further purification. All experiments were carried out under argon atmosphere in flame-dried glassware using standard inert techniques for introducing reagents and solvents unless otherwise noted. 3,5-Diphenyl-4,4,5-trifluoroisoxazoline (3) was synthesized according to the previous paper and used as a solution of 2.0 M in cyclohexane.[1]

Experimental Section:

General procedure for the synthesis of 4,4,5-trifluoroisoxazolines.[1]

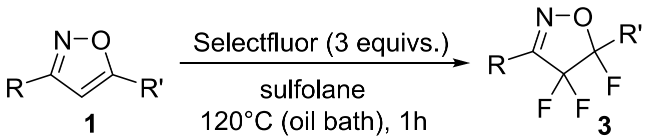

Isoxazole (**1**; 1 mmol) and Selectfluor (3 mmol) were suspended in sulfolane (4 mL) and stirred for 1 h at 120 °C. The resulting mixture was quenched with saturated aqueous $NaHCO_3$ and extracted with AcOEt. The AcOEt layer was washed with saturated aqueous NaCl and dried over $MgSO_4$. The solvent was removed in vacuo and the residue was purified by column chromatography to give 4,4,5-trifluorinated isoxazole (**3**).

General procedure for S_EAr reaction with fluorinated isoxazolines.

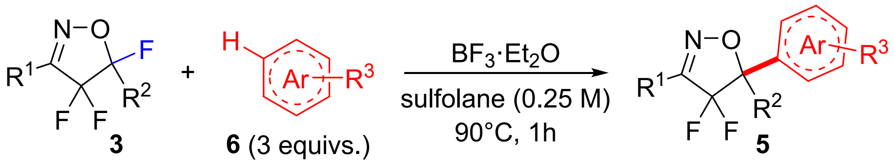

To a solution of 2.0 M 4,4,5-trifluoroisoxazoline (**3**) in cyclohexane (0.5 mL, 1 mmol of **3**) was added sulfolane (3.5 mL) and aromatics (**6**, 3 mmol) followed by $BF_3{\cdot}Et_2O$ (1 mmol) at ambient temperature. The reaction mixture was stirred at 90°C for 1 h. After that time, the resulting mixture was quenched with saturated aqueous $NaHCO_3$ and extracted with AcOEt. The AcOEt layer was washed with saturated aqueous NaCl and dried over $MgSO_4$. The solvent was removed in vacuo and the residue was purified by column chromatography to give 5-arylated 4,4-difluoroisoxazoline (**5**).

Typical procedure for the synthesis of 6 via reductive N-O bond cleavage.[2]

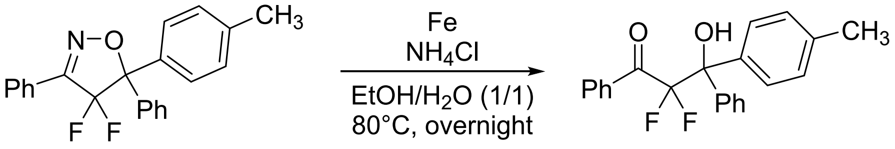

5aB **6**

The mixture of **5aB** (141 mg, 0.4 mmol), Fe powder (223 mg, 4 mmol), and NH_4Cl (214 mg, 4 mmol) of $EtOH/H_2O$ (1/1) 20 mL was stirred overnight at 80 °C. After that time, the reaction mixture was filtered through Celite, then the filtrate was diluted with AcOEt and washed with saturated aqueous NaCl. The AcOEt layer was separated and dried over $MgSO_4$. The solvent was removed in vacuo and the residue was purified by column chromatography to give the keto alcohol (**6**, 96 mg, 68%).

Typical procedure for the synthesis of 7 via reductive N-O bond cleavage.[3]

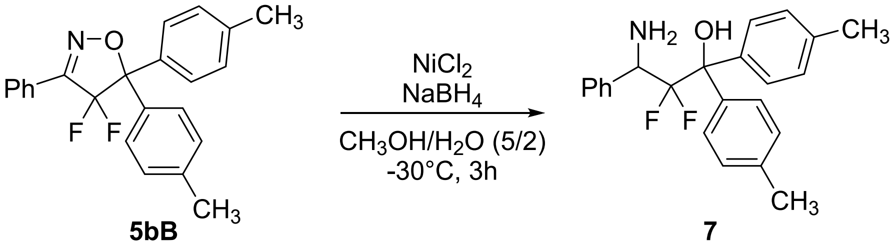

5bB **7**

The solution of **5bB** (200 mg, 0.55 mmol) and $NiCl_2$ (392 mg, 1.65 mmol) in MeOH (11 mL) and THF (4.4 mL) was cooled to -30°C and stirred for 10 min. After that time, $NaBH_4$ (209 mg, 5.5 mmol) was added and the mixture was stirred for 3 h under the same temperature. The resulting mixture was quenched with H_2O and extracted with AcOEt. The AcOEt layer was washed with saturated aqueous NaCl and dried over $MgSO_4$. The solvent was removed in vacuo and the residue was purified by column chromatography to give the amino alcohol (**7**, 125 mg, 62%).

Spectroscopic Data:

4,4-Difluoro-3,5-diphenyl-4,5-dihydroisoxazol-5-ol (4a)[1]

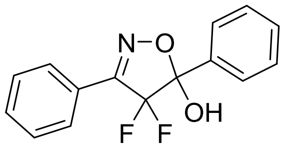

Colorless solid; M.p. 109.0–111.0 °C (recrystallized from hexane–AcOEt); ^{1}H NMR (400 MHz, $CDCl_3$) δ: 3.96 (1H, s), 7.41–7.53 (6H, m), 7.63–7.66 (2H, m), 7.83–7.85 (2H, m); ^{13}C NMR (100 MHz, $CDCl_3$) δ: 103.0 (dd, *J* = 33.1, 19.4 Hz), 124.6 (dd, *J* = 267.8, 255.2 Hz), 124.9 (m), 126.8 (d, *J* = 1.5 Hz), 127.1 (d, *J* = 1.5 Hz), 128.5, 129.1, 130.3, 131.6, 132.7 (d, *J* = 1.4 Hz), 153.7 (dd, *J* = 25.9, 24.9 Hz); ^{19}F NMR (376 MHz, $CDCl_3$) δ: -37.54 (1F, d, *J* = 266.3 Hz), -57.23 (1F, d, *J* = 266.3 Hz); MS *m/z*: 275 (M^+); HRMS Calcd for $C_{15}H_{11}F_2NO_2$: 275.076 (M^+), Found: 275.076; IR (KBr) cm^{-1}: 2982, 1450, 1365, 1242, 1127, 1098.

4,4-difluoro-3,5-diphenyl-5-(*p*-tolyl)-4,5-dihydroisoxazole (5aB)

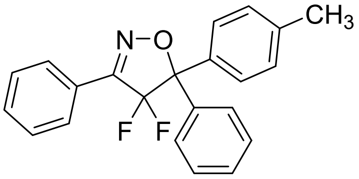

Colorless solid; M.p. 86.5–88.0 °C (recrystallized from hexane); ^{1}H NMR (400 MHz, $CDCl_3$) δ: 2.33 (3H, s), 7.17–7.19 (2H, m), 7.31–7.49 (8H, m), 7.54–7.56 (2H, m), 7.86–7.87 (2H, m); ^{13}C NMR (100 MHz, $CDCl_3$) δ: 21.09, 90.77 (t, *J* = 23.0 Hz), 125.3, 126.8, 127.1, 127.2, 127.2, 128.2 (t, *J* = 260.1 Hz), 128.3, 128.5, 129.0 (d, *J* = 3.3 Hz), 131.2, 132.6 (t, *J* = 2.5 Hz), 135.6 (t, *J* = 2.4 Hz), 138.5, 153.57 (t, *J* = 25.8 Hz).; ^{19}F NMR (376 MHz, $CDCl_3$) δ: -97.41 (1F, d, *J* = 258.1 Hz), -98.48 (1F, d, *J* = 258.1 Hz); MS *m/z*: 349 (M^+); HRMS Calcd for $C_{22}H_{17}F_2NO$: 349.1278 (M^+), Found: 349.1275.

4,4-difluoro-5-(4-methoxyphenyl)-3,5-diphenyl-4,5-dihydroisoxazole (5aC)

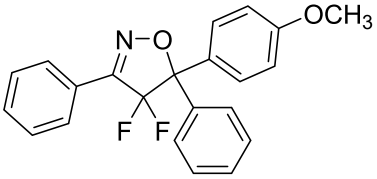

Colorless solid; M.p. 77.0–79.0 °C (recrystallized from hexane); ^{1}H NMR (400 MHz, $CDCl_3$) δ: 3.79 (3H, s), 6.87–6.91 (2H, m), 7.32–7.47 (8H, m), 7.54–7.56 (2H, m), 7.85–7.87 (2H, m); ^{13}C NMR (100 MHz, $CDCl_3$) δ: 55.26, 90.7 (t, *J* = 23.2 Hz) , 113.7, 125.3, 126.8, 127.20 (d, *J* = 1.6 Hz) , 127.6 (t, *J* = 3.0 Hz) , 128.2 (t, *J* = 259.9 Hz) , 128.3, 128.5, 128.7 (d, *J* = 1.4 Hz) , 129.0, 131.2, 135.7, 153. 6 (t, *J* = 25.9 Hz), 159.7; ^{19}F NMR (376 MHz, $CDCl_3$) δ: -96.99 (1F, d, *J* = 258.0 Hz), -98.78 (1F, d, *J* = 258.0 Hz); MS *m/z*: 365 (M^+); HRMS Calcd for $C_{22}H_{17}F_2NO_2$: 365.1227 (M^+), Found: 365.1230.

4-(4,4-difluoro-3,5-diphenyl-4,5-dihydroisoxazol-5-yl)-*N*,*N*-dimethylaniline (5aD)

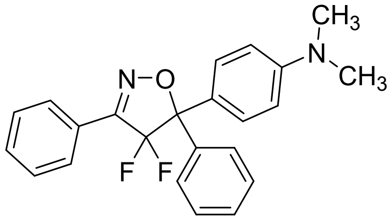

Yellowish solid; M.p. 130.0–131.5 °C; ^{1}H NMR (400 MHz, $CDCl_3$) δ: 2.93 (6H, s), 6.65–6.69 (2H, m), 7.33–7.49 (8H, m), 7.55–7.58 (2H, m), 7.86–7.88 (2H, m); ^{13}C NMR (100 MHz, $CDCl_3$) δ: 40.25, 91.09 (t, *J* = 23.0 Hz), 111.7, 122.8 (t, *J* = 2.6 Hz), 125.5, 126.8, 128.1, 127.3 (d, *J* = 1.6 Hz), 128.2 (t, *J* = 259.3 Hz), 128.3, 128.3, 128.9, 131.0, 135.9, 150.3, 153.5 (t, *J* = 26.0 Hz); ^{19}F NMR (376 MHz, $CDCl_3$) δ: -96.18 (1F, d, *J* = 257.8 Hz), -99.64 (1F, d, *J* = 257.8 Hz); MS *m/z*: 378 (M^+); HRMS Calcd for $C_{23}H_{20}F_2N_2O$: 378.1544 (M^+), Found: 378.1542.

5-(3,4-dimethylphenyl)-4,4-difluoro-3,5-diphenyl-4,5-dihydroisoxazole (5aH)

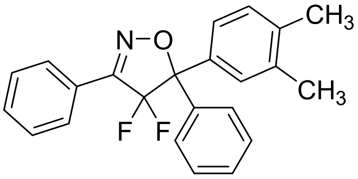

Colorless solid; M.p. 102.0–105.0 °C (recrystallized from hexane); ^{1}H NMR (400 MHz, $CDCl_3$) δ: 2.24 (3H, s), 2.26 (3H, s), 7.14 (1H, d, *J* = 7.9 Hz), 7.17–7.49 (8H, m), 7.54–7.56 (2H, m), 7.85–7.87 (2H, m); ^{13}C NMR (100 MHz, $CDCl_3$) δ: 19.45, 20.03, 90.74 (t, *J* = 23.1 Hz), 124.6, 125.3, 126.8, 127.2, 128.2 (t, *J* = 260.1 Hz), 128.2, 128.3, 128.4, 129.0, 129.5, 131.2, 132.9 (t, *J* = 2.4 Hz), 135.8 (t, *J* = 2.4 Hz), 136.6, 137.2, 153.6 (t, *J* = 25.9 Hz); ^{19}F NMR (376 MHz, $CDCl_3$) δ: -97.92 (2F, s); MS *m/z*: 363 (M^+); HRMS Calcd for $C_{23}H_{19}F_2NO$: 363.1435 (M^+), Found: 363.1439.

5-(2,4-dimethylphenyl)-4,4-difluoro-3,5-diphenyl-4,5-dihydroisoxazole (5aI)

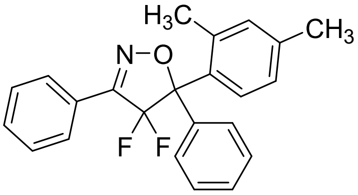

Colorless solid; M.p. 148.5–150.5 °C (recrystallized from hexane–AcOEt); ^{1}H NMR (400 MHz, $CDCl_3$) δ: 2.20 (3H, s), 2.36 (3H, s), 7.07 (1H, m), 7.13 (1H, m), 7.26–7.30 (5H, m), 7.43–7.51 (3H, m), 7.73 (1H, m), 7.88–7.90 (2H, m); ^{13}C NMR (100 MHz, $CDCl_3$) δ: 20.98, 21.46, 92.52 (t, *J* = 22.9 Hz), 125.3 (d, *J* = 1.5 Hz), 126.2, 126.8 (d, *J* = 0.6 Hz),127.1 (d, *J* = 3.7 Hz), 127.9 (d, *J* = 1.5 Hz), 128.2, 128.7, 128.8 (dd, *J* = 261.8, 258.4 Hz), 129.0, 130.0 (d,

J = 3.5 Hz), 131.1, 133.0, 136.1 (d, J = 4.1 Hz), 137.6, 138.5, 153.4 (t, J = 26.1 Hz); ^{19}F NMR (376 MHz, $CDCl_3$) δ: -88.58 (1F, d, J = 260.6 Hz), -106.3 (1F, d, J = 260.6 Hz); MS m/z: 363 (M^+); HRMS Calcd for $C_{23}H_{19}F_2NO$: 363.1435 (M^+), Found: 363.1432.

4,4-difluoro-5-(1-methyl-1*H*-pyrrol-2-yl)-3,5-diphenyl-4,5-dihydroisoxazole (5aJ)

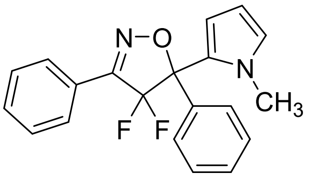

Pale yellow solid; M.p. 93.0–95.5 °C (recrystallized from hexane); ^{1}H NMR (400 MHz, $CDCl_3$) δ: 3.33 (3H, s), 6.10 (1H, m), 6.58 (1H, m), 6.65 (1H, m), 7.33–7.38 (5H, m), 7.42–7.51 (3H, m), 7.84–7.86 (2H, m); ^{13}C NMR (100 MHz, $CDCl_3$) δ: 35.95, 88.75 (dd, J = 25.2, 21.2 Hz), 106.6 (d, J = 1.2 Hz), 111.9 (d, J = 11.9 Hz), 125.0 (d, J = 3.4 Hz), 125.2, 125.8, 126.8, 127.5 (d, J = 1.0 Hz), 127.8 (dd, J = 264.9, 257.3 Hz), 128.4, 128.9, 129.0, 131.2, 134.4 (m), 153.7 (t, J = 25.4 Hz); ^{19}F NMR (376 MHz, $CDCl_3$) δ: -97.98 (1F, d, J = 259.2 Hz), -103.9 (1F, dd, J = 259.2, 5.2 Hz); MS m/z: 338 (M^+); HRMS Calcd for $C_{20}H_{16}F_2N_2O$: 338.1231 (M^+), Found: 338.1233.

4,4-difluoro-5-(1-methyl-1*H*-pyrrol-3-yl)-3,5-diphenyl-4,5-dihydroisoxazole (5aJ')

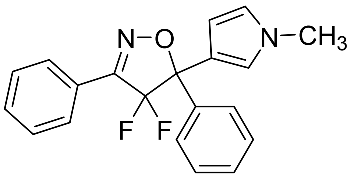

Pale yellow oil; ^{1}H NMR (400 MHz, $CDCl_3$) δ: 3.56 (3H, s), 6.13 (1H, m), 6.52–6.55 (2H, m), 7.34–7.48 (6H, m), 7.61–7.63 (2H, m), 7.86–7.89 (2H, m); ^{13}C NMR (100 MHz, $CDCl_3$) δ: 36.29, 89.19 (t, J = 23.0 Hz), 108.8 (d, J = 3.3 Hz), 119.7 (t, J = 3.6 Hz), 122.2, 122.4 (d, J = 2.9 Hz), 125.7 (m), 126.7, 127.0 (d, J = 1.0 Hz), 127.4 (dd, J = 260.1, 256.8 Hz), 128.0, 128.3, 128.9, 130.9, 135.4 (m), 153.3 (t, J = 25.9 Hz); ^{19}F NMR (376 MHz, $CDCl_3$) δ: -95.14 (1F, d, J = 258.2 Hz), -104.1 (1F, d, J = 258.2 Hz); MS m/z: 338 (M^+); HRMS Calcd for $C_{20}H_{16}F_2N_2O$: 338.1231 (M^+), Found: 338.1232.

4,4-difluoro-5-(furan-2-yl)-3,5-diphenyl-4,5-dihydroisoxazole (5aK)

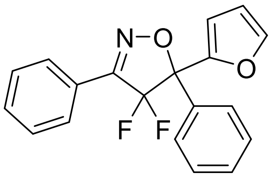

Pale yellow solid; M.p. 61.0–65.0 °C; ^{1}H NMR (400 MHz, $CDCl_3$) δ: 6.30–6.32 (2H, m), 7.40–7.51 (7H, m), 7.64–7.66 (2H, m), 7.87–7.90 (2H, m); ^{13}C NMR (100 MHz, $CDCl_3$) δ: 87.26 (dd, J = 26.7, 22.7 Hz), 110.1, 111.9, 125.1, 126.8, 127.4 (dd, J = 261.9, 258.0 Hz),

127.9 (d, $J = 11.7$ Hz), 128.3, 129.0, 129.2, 131.3, 132.1 (d, $J = 3.9$ Hz), 144.2, 149.3 (m), 153.0 (t, $J = 25.5$ Hz); ^{19}F NMR (376 MHz, $CDCl_3$) δ: -90.86 (1F, d, $J = 261.3$ Hz), -106.1 (1F, d, $J = 261.3$ Hz); MS *m/z*: 325 (M^+); HRMS Calcd for $C_{19}H_{13}F_2NO_2$: 325.0914 (M^+), Found: 325.0916.

4,4-difluoro-3,5-diphenyl-5-(thiophen-2-yl)-4,5-dihydroisoxazole (5aL)

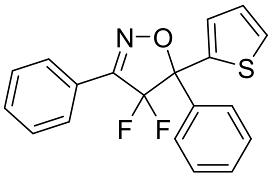

Colorless solid; M.p. 65.5–66.5 °C (recrystallized from hexane); ^{1}H NMR (400 MHz, $CDCl_3$) δ: 6.98 (1H, dd, $J = 5.1, 3.8$ Hz), 7.15 (1H, m), 7.33 (1H, dd, $J = 5.1, 1.2$ Hz), 7.39–7.52 (6H, m), 7.64–7.67 (2H, m), 7.86–7.88 (2H, m); ^{13}C NMR (100 MHz, $CDCl_3$) δ: 89.30 (dd, $J = 25.8, 22.4$ Hz), 125.1, 126.8, 126.8, 126.9, 127.0, 127.3 (dd, $J = 262.0, 259.4$ Hz), 128.3, 128.4, 128.4, 129.0, 131.4, 134.5 (d, $J = 3.1$ Hz), 139.4 (m), 153.6 (t, $J = 25.7$ Hz); ^{19}F NMR (376 MHz, $CDCl_3$) δ: -93.54 (1F, d, $J = 258.0$ Hz), -103.93 (1F, d, $J = 258.0$ Hz); MS *m/z*: 341 (M^+); HRMS Calcd for $C_{19}H_{13}F_2NOS$: 341.0686 (M^+), Found: 341.0687.

4,4-difluoro-3-phenyl-5,5-di-*p*-tolyl-4,5-dihydroisoxazole (5bB)

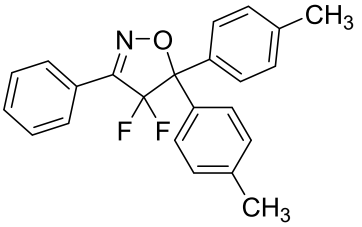

Pale yellow oil; ^{1}H NMR (400 MHz, $CDCl_3$) δ: 2.33 (6H, s), 7.15–7.18 (4H, m), 7.40–7.48 (7H, m), 7.85–7.89 (2H, m); ^{13}C NMR (100 MHz, $CDCl_3$) δ: 21.08, 90.80 (t, $J = 23.1$ Hz), 125.3 (m), 126.8, 127.1, 128.2 (t, $J = 259.8$ Hz), 129.0, 129.0, 131.1, 132.7 (t, $J = 2.4$ Hz), 138.4, 153.5 (t, $J = 25.8$ Hz); ^{19}F NMR (376 MHz, $CDCl_3$) δ: -98.03 (2F, s); MS *m/z*: 363 (M^+); HRMS Calcd for $C_{23}H_{19}F_2NO$: 363.1435 (M^+), Found: 363.1432.

3,5-bis(4-chlorophenyl)-4,4-difluoro-5-(*p*-tolyl)-4,5-dihydroisoxazole (5cB)

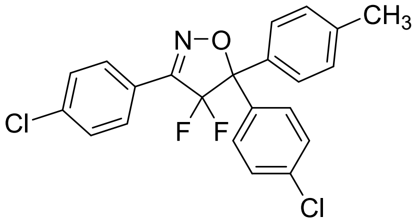

Colorless oil; ^{1}H NMR (400 MHz, $CDCl_3$) δ: 2.34 (3H, s), 7.19–7.21 (2H, m), 7.34–7.39 (4H, m), 7.42–7.48 (4H, m), 7.78–7.80 (2H, m); ^{13}C NMR (100 MHz, $CDCl_3$) δ: 21.11, 90.55 (t, $J = 23.5$ Hz), 123.5, 127.0, 127.9 (t, $J = 260.1$ Hz), 128.0, 128.6, 128.6, 129.2, 129.4, 131.1

(m), 134.0 (m), 134.7, 137.6, 138.9, 152.8 (t, J = 26.3 Hz); ^{19}F NMR (376 MHz, $CDCl_3$) δ: -97.48 (1F, d, J = 258.2 Hz), -98.40 (1F, d, J = 258.2 Hz); MS *m/z*: 417 (M^+); HRMS Calcd for $C_{22}H_{15}Cl_2F_2NO$: 417.0499 (M^+), Found: 417.0499.

4,4-difluoro-3-methyl-5-phenyl-5-(*p*-tolyl)-4,5-dihydroisoxazole (5dB)

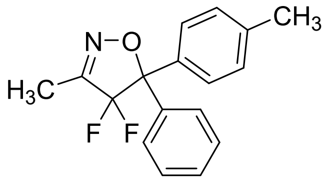

Pale yellow oil; ^{1}H NMR (400 MHz, $CDCl_3$) δ: 2.11 (3H, t, J = 1.5 Hz), 2.33 (3H, s), 7.16–7.18 (2H, m), 7.30–7.39 (5H, m), 7.49–7.51 (2H, m); ^{13}C NMR (100 MHz, $CDCl_3$) δ: 8.56, 21.08, 88.83 (t, J = 22.4 Hz), 127.7 (t, J = 258.5 Hz), 127.0 (m), 127.0 (m), 128.2, 128.3, 129.0, 132.8 (t, J = 2.7 Hz), 135.8 (t, J = 2.5 Hz), 138.3, 152.9 (t, J = 27.9 Hz); ^{19}F NMR (376 MHz, $CDCl_3$) δ: -102.5 (dq, J = 258.9, 1.5 Hz), -103.6 (dq, J = 258.9, 1.6 Hz); MS *m/z*: 287 (M^+); HRMS Calcd for $C_{17}H_{15}F_2NO$: 287.1122 (M^+), Found: 287.1123.

2,2-difluoro-3-hydroxy-1,3-diphenyl-3-(*p*-tolyl)propan-1-one (6)

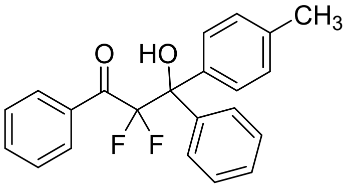

Pale yellow oil; ^{1}H NMR (400 MHz, $CDCl_3$) δ: 2.33 (3H, s), 4.27 (1H, s), 7.12–7.14 (2H, m), 7.28–7.37 (5H, m), 7.45–7.49 (4H, m), 7.62–7.67 (1H, m), 8.00–8.03 (2H, m); ^{13}C NMR (100 MHz, $CDCl_3$) δ: 21.07, 80.25 (t, J = 23.0 Hz), 115.5 (t, J = 264.9 Hz), 127.4, 127.6, 127.9, 128.0, 128.7, 128.8, 130.3 (t, J = 3.1 Hz), 132.6 (t, J = 2.7 Hz), 134.7, 137.8, 137.9, 140.8, 191.9 (t, J = 31.6 Hz); ^{19}F NMR (376 MHz, $CDCl_3$) δ: -101.5 (2F, s); MS *m/z*: 352 (M^+); HRMS Calcd for $C_{22}H_{18}F_2O_2$: 352.1275 (M^+), Found: 352.1278.

3-amino-2,2-difluoro-3-phenyl-1,1-di-*p*-tolylpropan-1-ol (7)

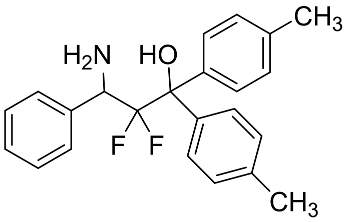

Colorless solid; M.p. 181.5–188.0 °C; ^{1}H NMR (400 MHz, $CDCl_3$) δ: 2.28 (3H, s), 2.39 (3H, s), 4.36 (1H, d, J = 26.5 Hz), 7.06–7.08 (2H, m), 7.23–7.29 (4H, m), 7.31–7.37 (3H, m), 7.45–7.47 (2H, m), 7.69–7.71 (2H, m); ^{13}C NMR (100 MHz, $CDCl_3$) δ: 20.98, 21.06, 58.15 (dd, J = 23.0, 31.7 Hz), 81.27 (t, J = 24.3 Hz), 120.7 (dd, J = 255.3, 260.5 Hz), 127.0 (d, J =

4.5 Hz), 127.8, 128.0, 128.3, 128.4, 128.6, 129.1, 137.0, 137.3, 137.8, 138.4, 140.6 (m); ^{19}F NMR (376 MHz, $CDCl_3$) δ: -100.6 (1F, d, *J* = 265.0 Hz), -124.0 (1F, dd, *J* = 26.5, 265.0 Hz); MS *m/z*: 367 (M^+); HRMS Calcd for $C_{23}H_{23}F_2NO$: 367.1748 (M^+), Found: 367.1747.

References:

[1] Sato, K.; Sandford, G.; Shimizu, K.; Akiyama, S.; Lancashire, M. J.; Yufit, D. S.; Tarui, A.; Omote, M.; Kumadaki, I.; Harusawa, S.; Ando, A. *Tetrahedron* **2016**, *72*, 1690–1698.

[2] Wang, L.; Zhang, K.; Wang, Y.; Li, W.; Chen, M.; Zhang, J. *Angew. Chem., Int. Ed.* **2020**, *59*, 4421–4427.

[3] Li, X.-T.; Gu, Q.-.; Dong, X.-Y.; Meng, X.; Liu, X.-Y. *Angew. Chem., Int. Ed.* **2018**, *57*, 7668–7672.

NMR charts:

^{1}H NMR of **5aB**

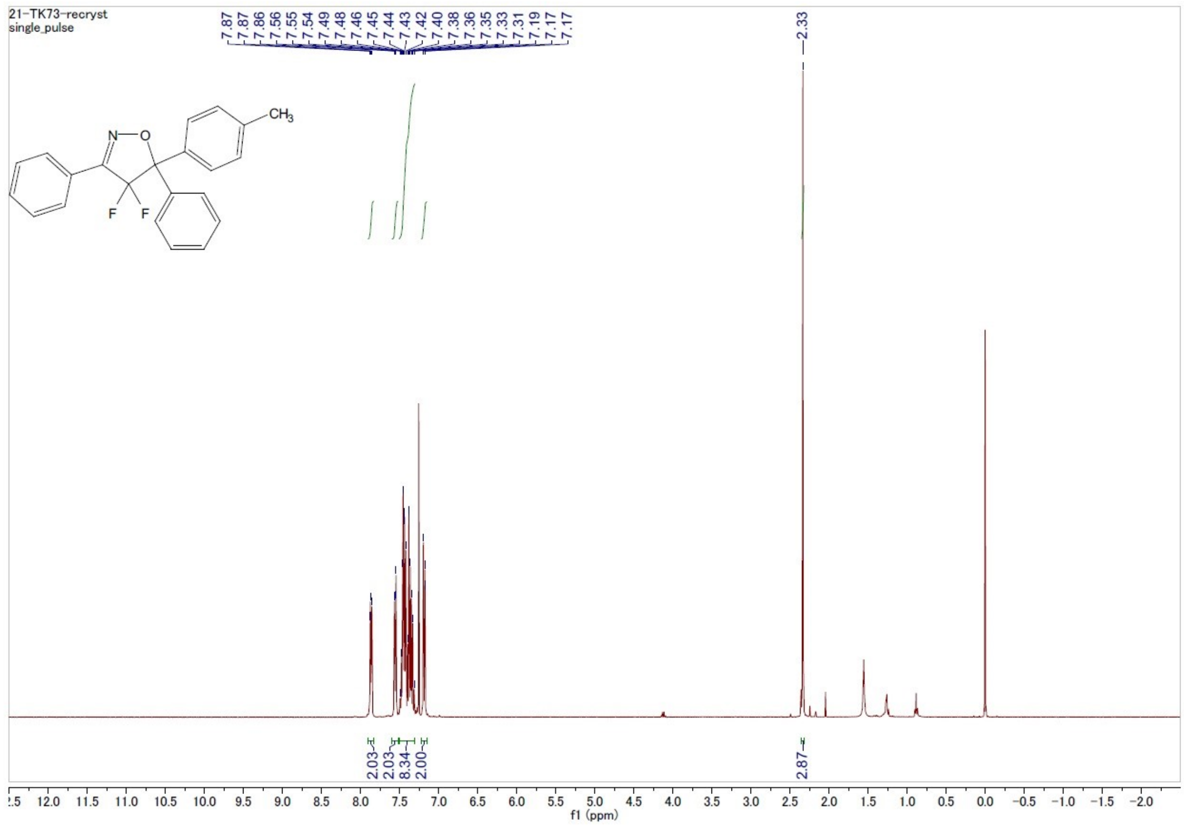

^{19}F NMR of **5aB**

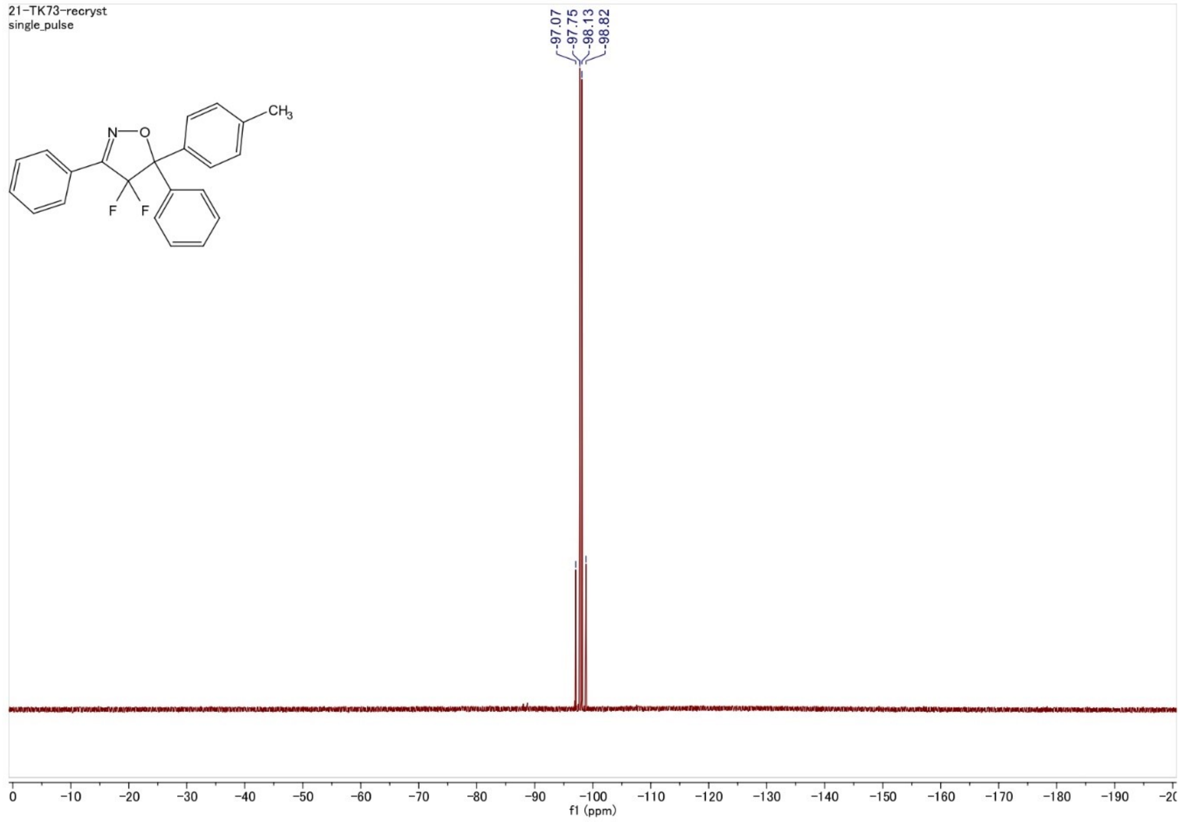

^{13}C NMR of **5aB**

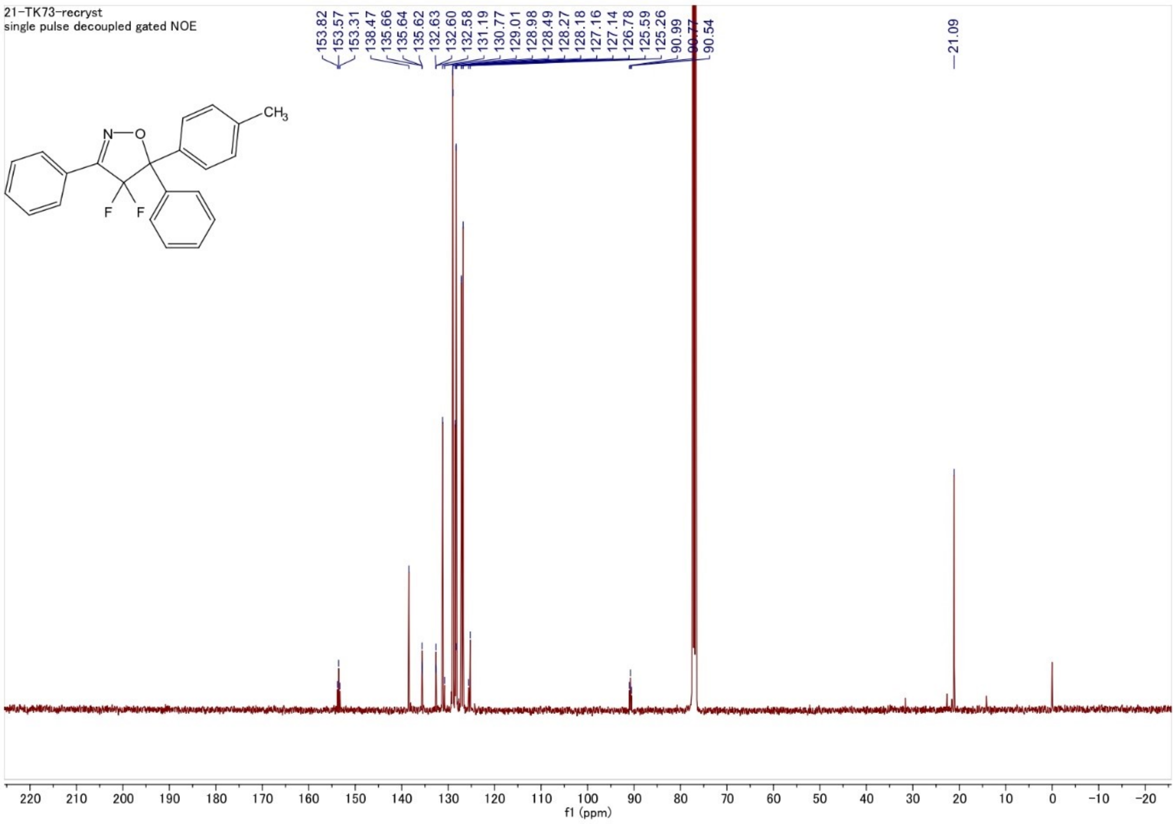

DEPT135 of **5aB**

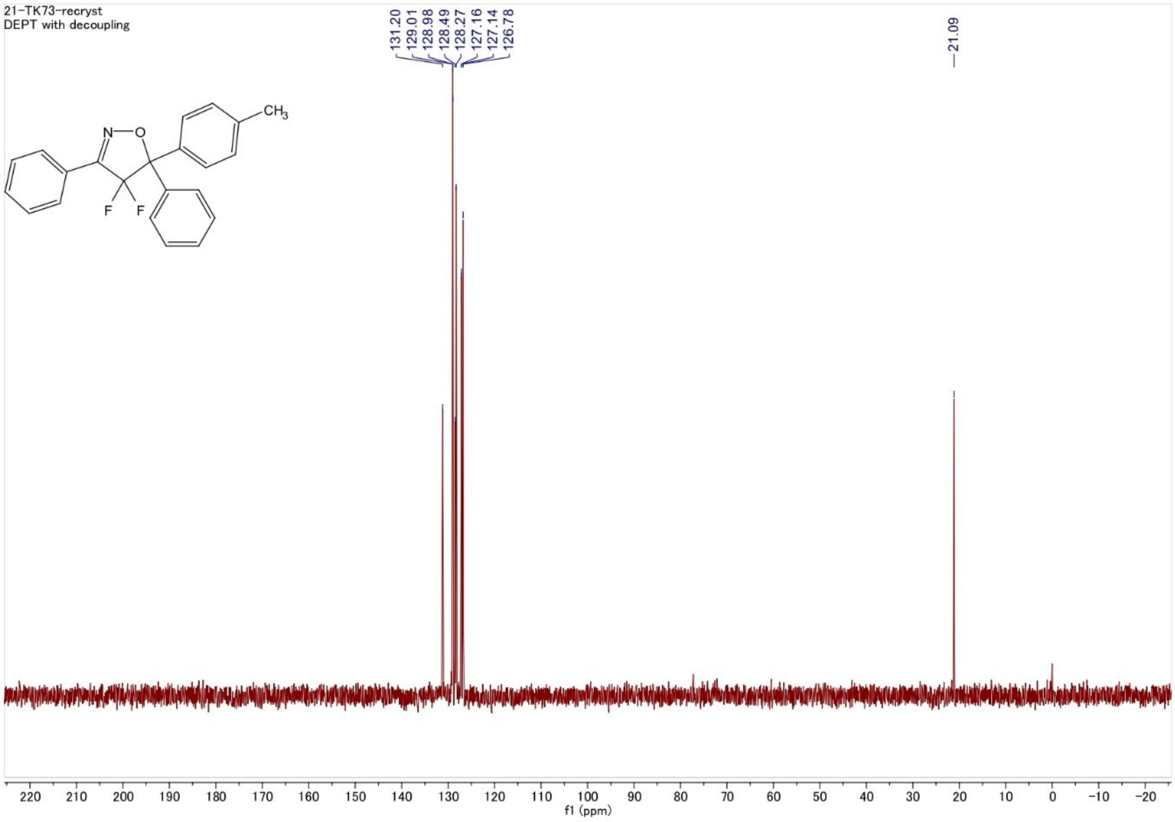

^{1}H NMR of **5aC**

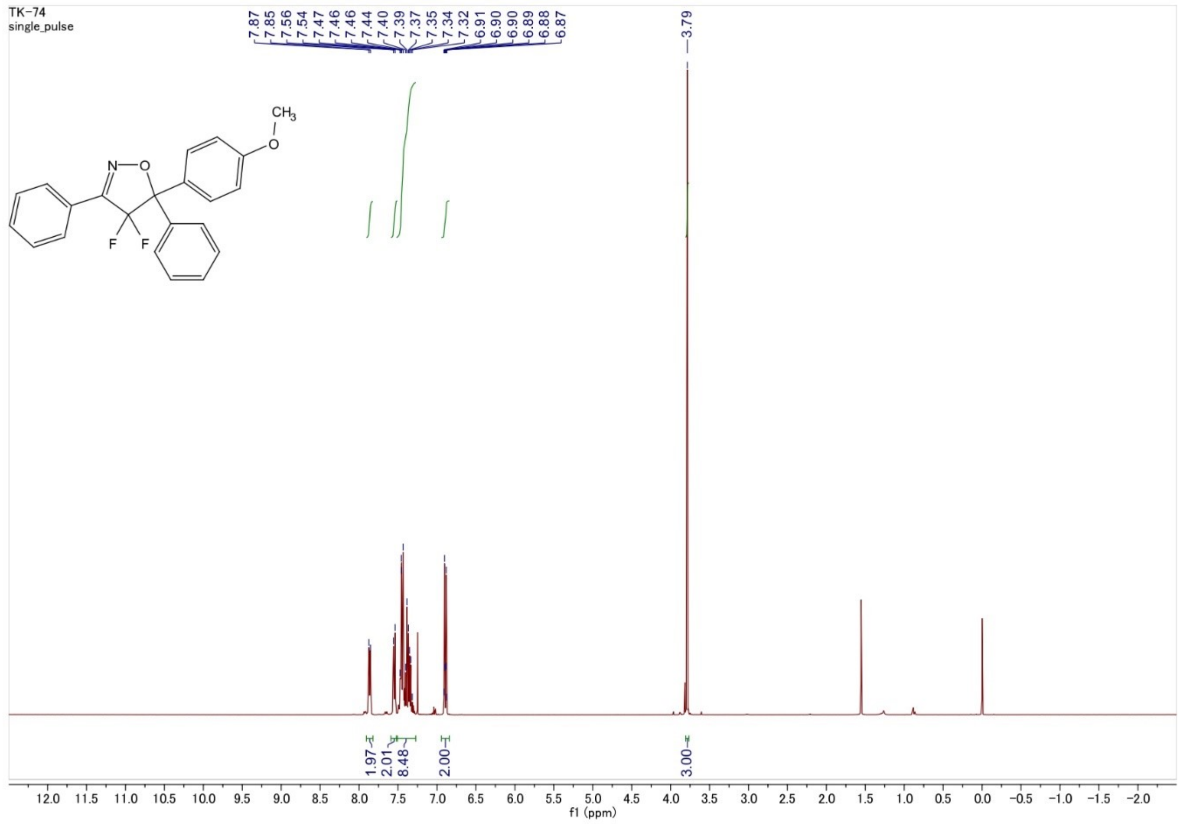

^{19}F NMR of **5aC**

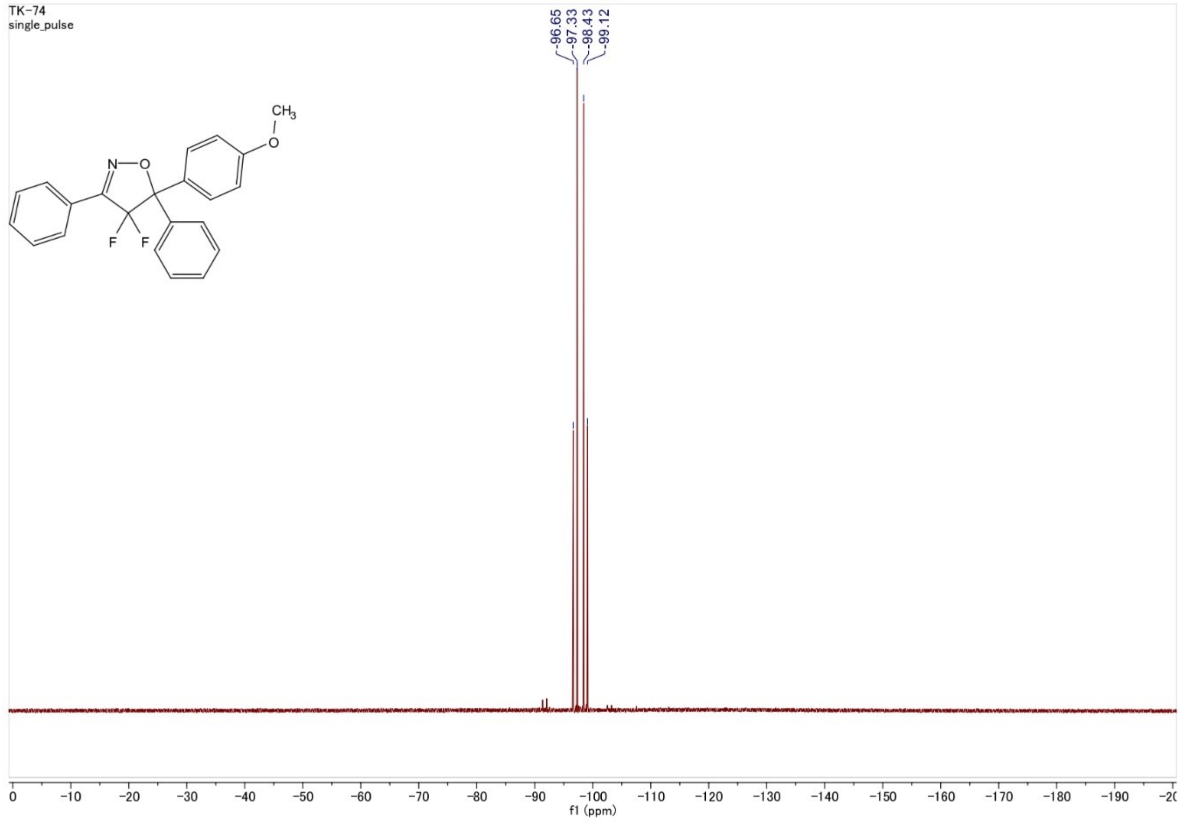

^{13}C NMR of **5aC**

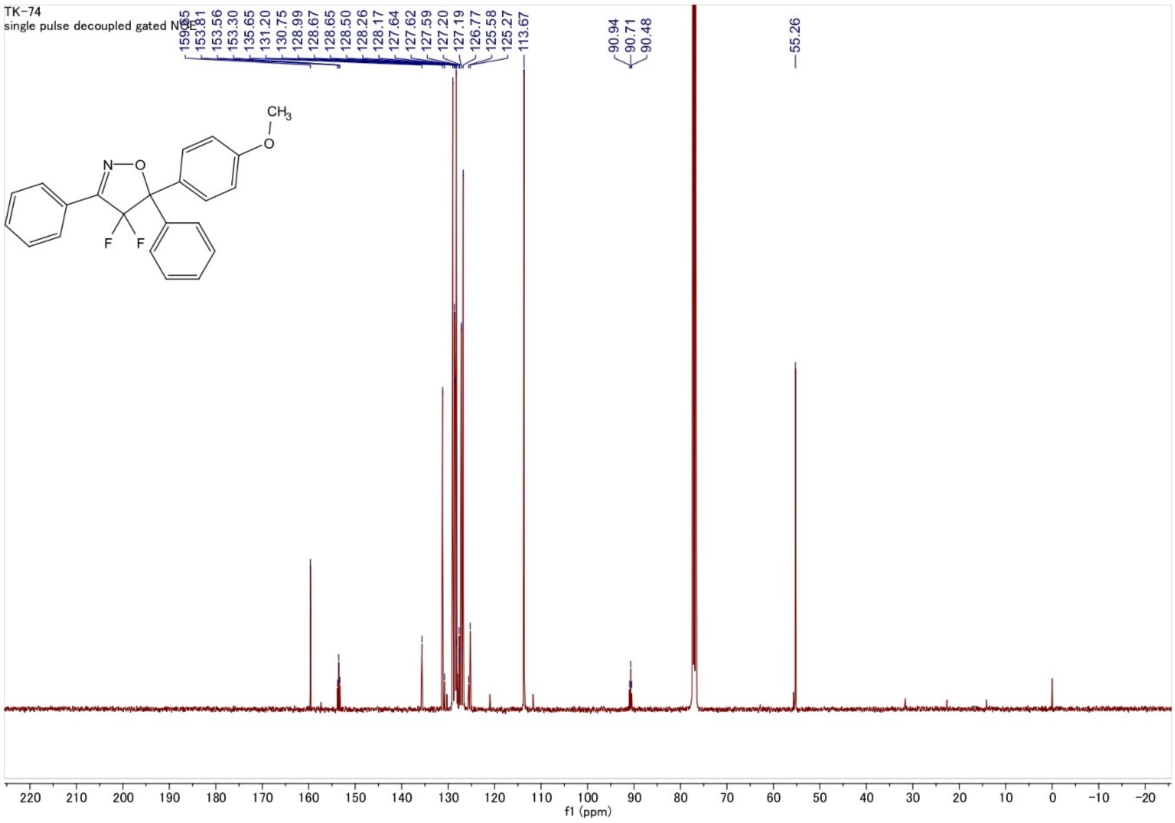

DEPT135 of **5aC**

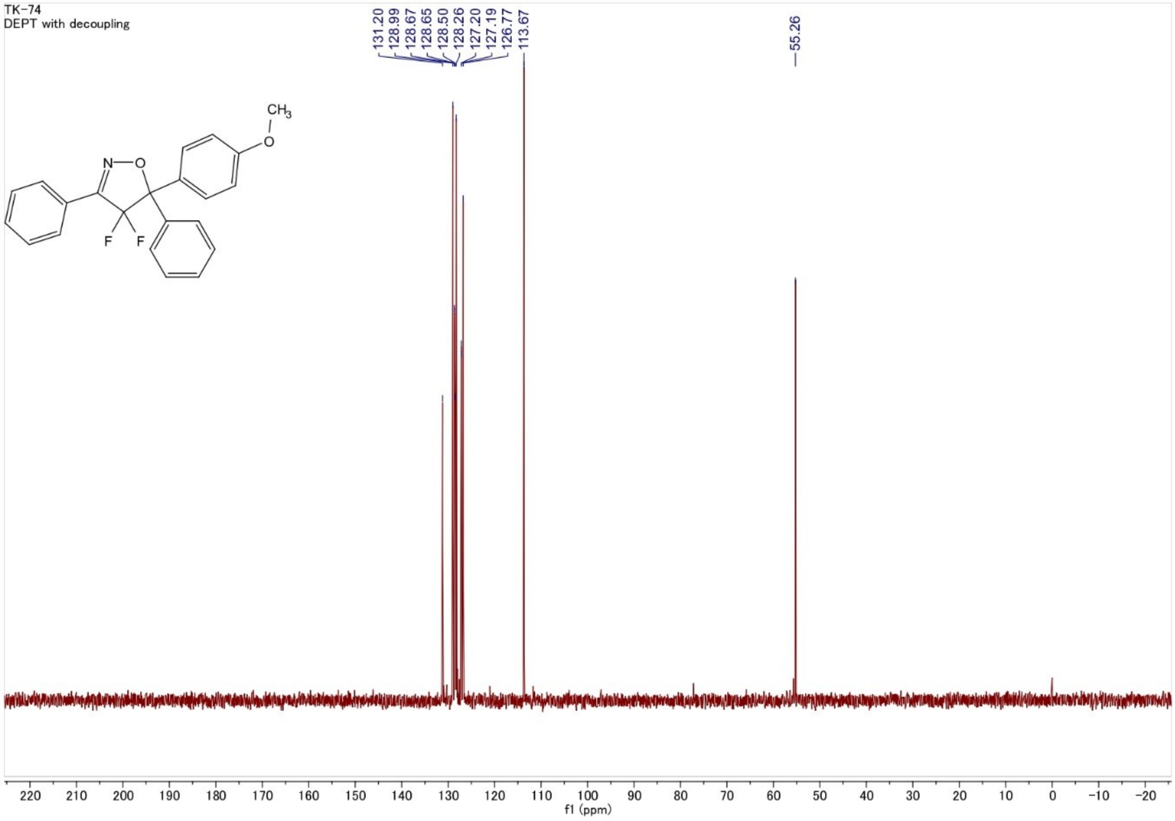

^{1}H NMR of **5aD**

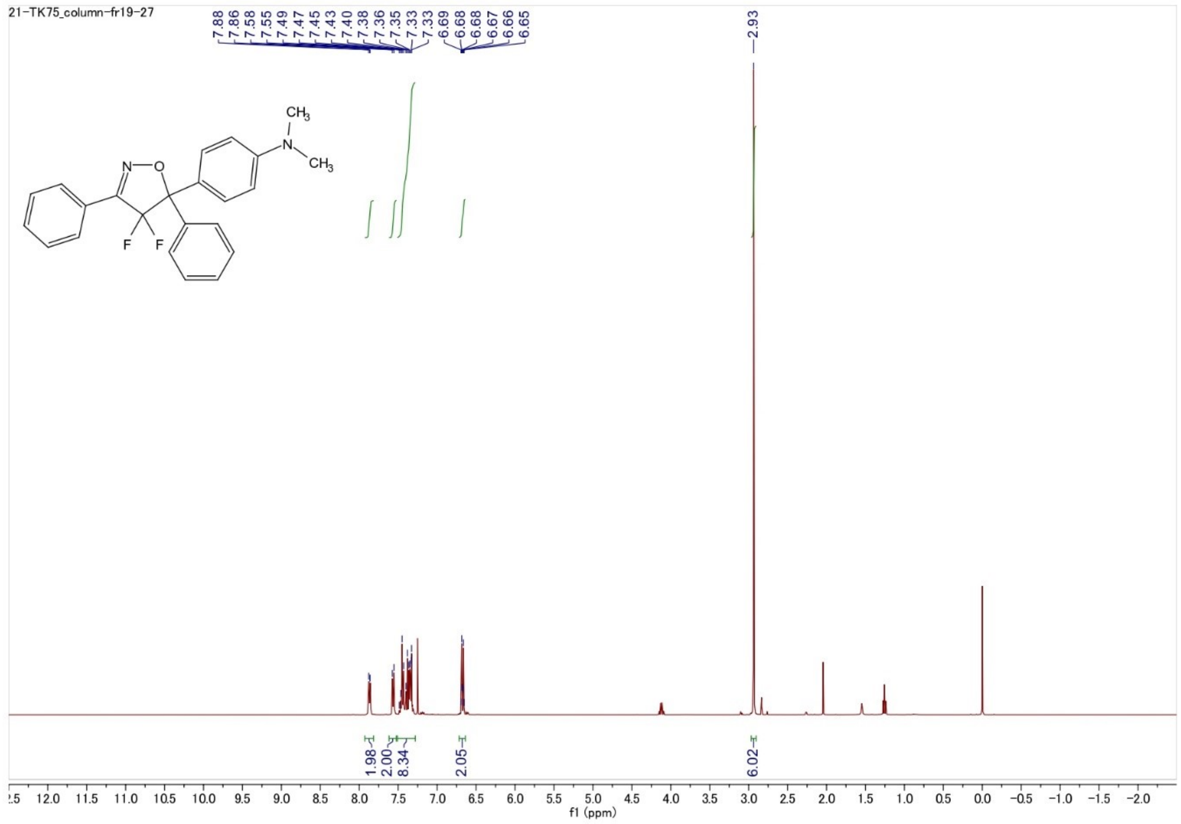

^{19}F NMR of **5aD**

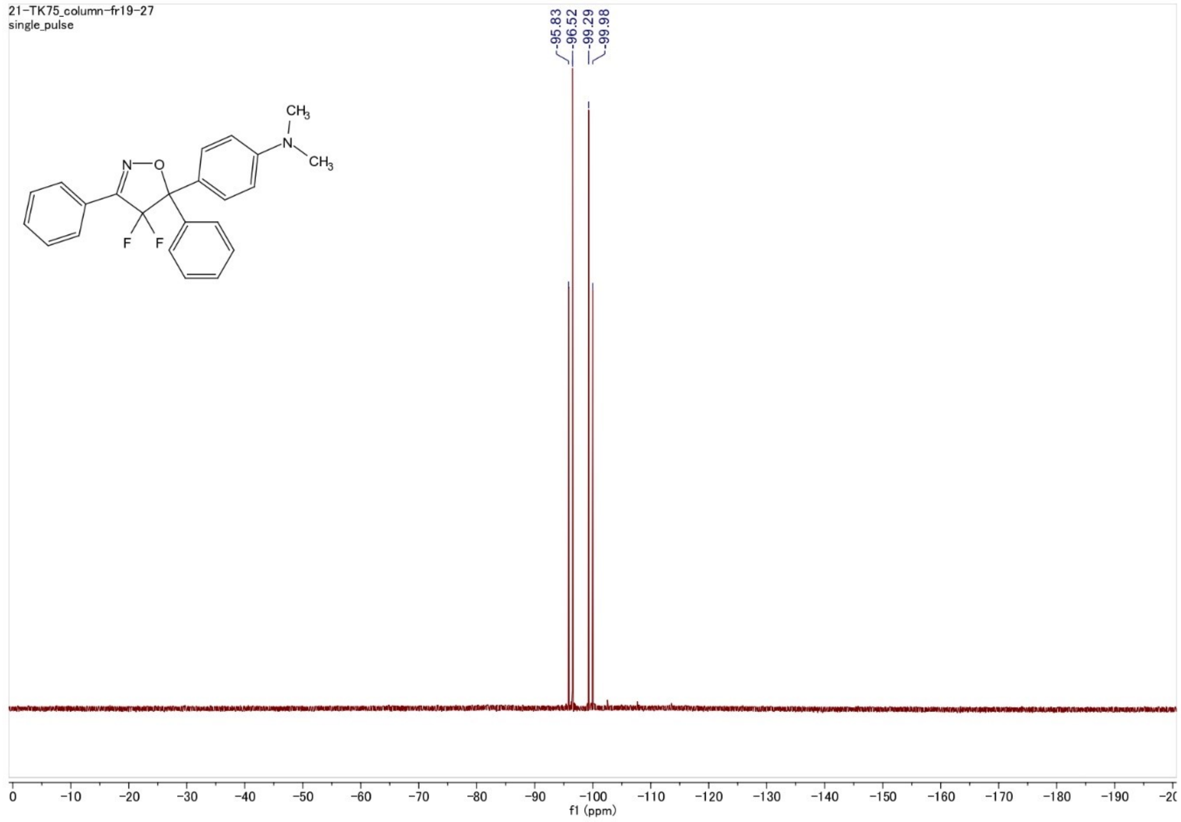

^{13}C NMR of **5aD**

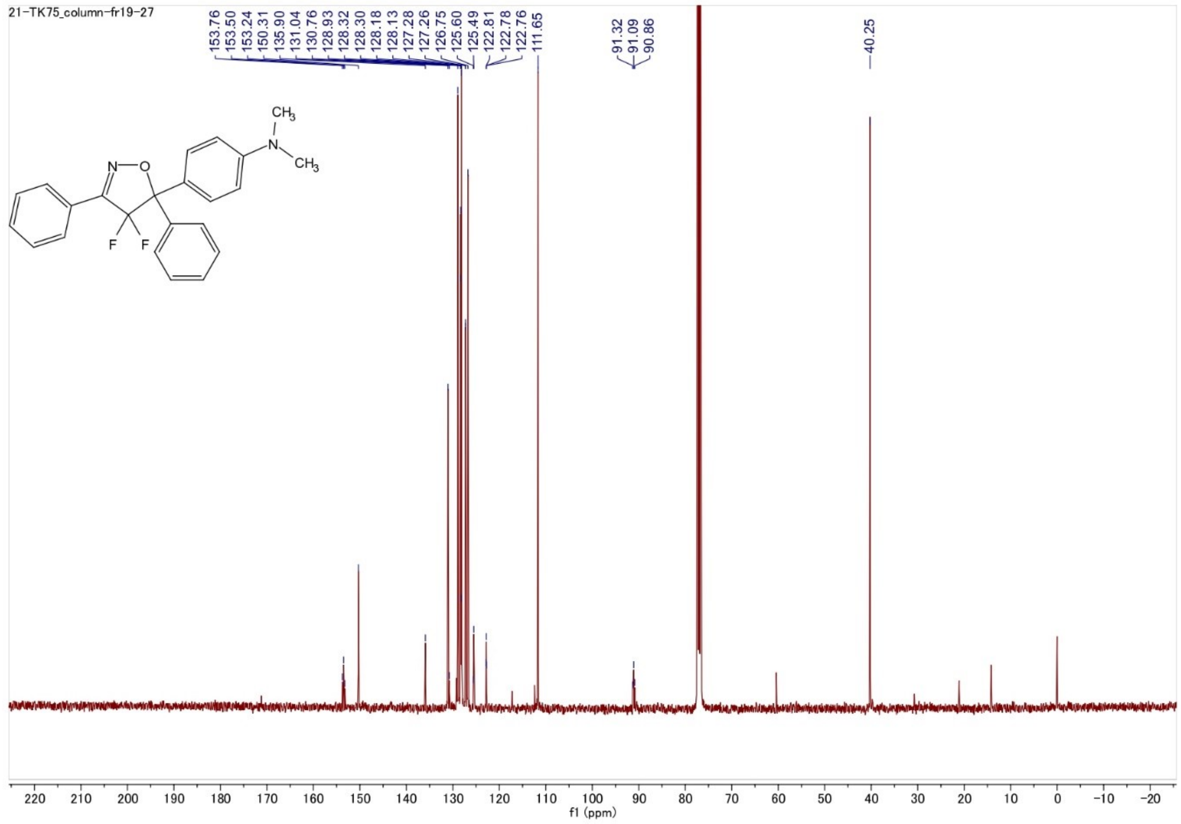

DEPT135 of **5aD**

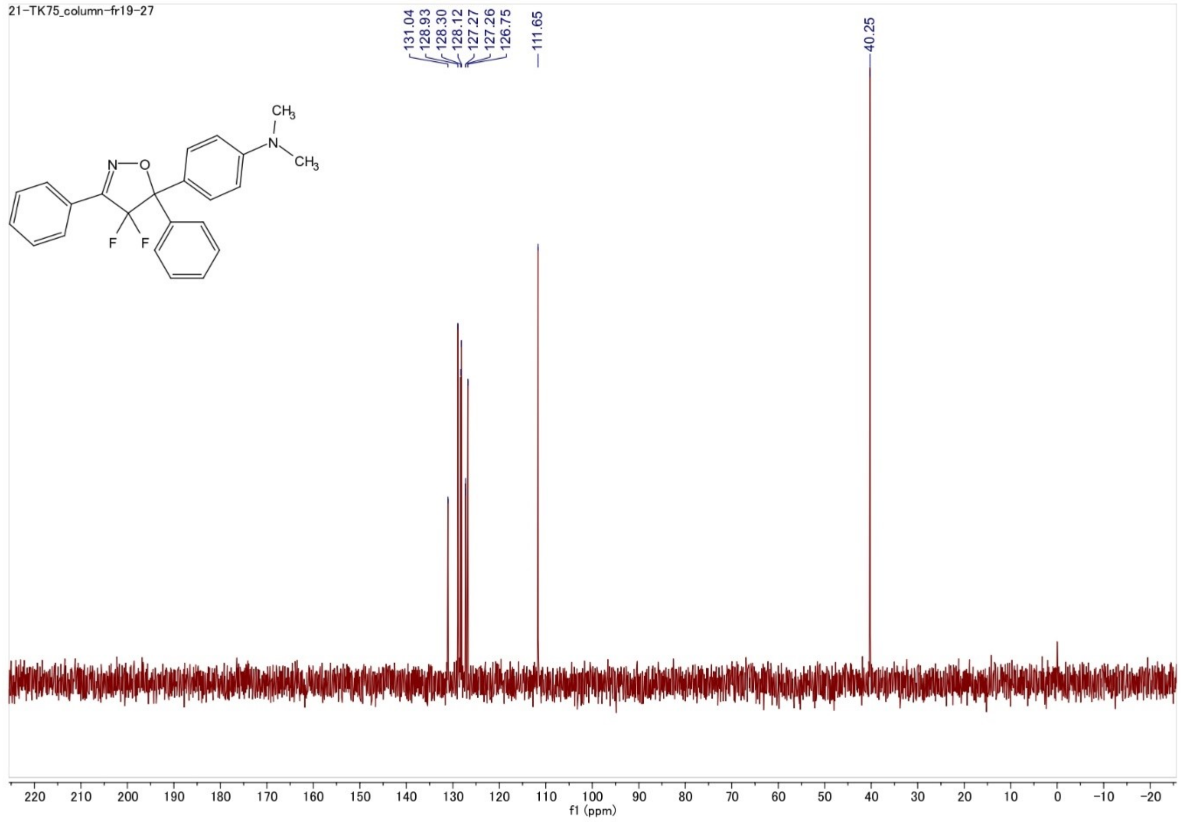

^{1}H NMR of **5aH**

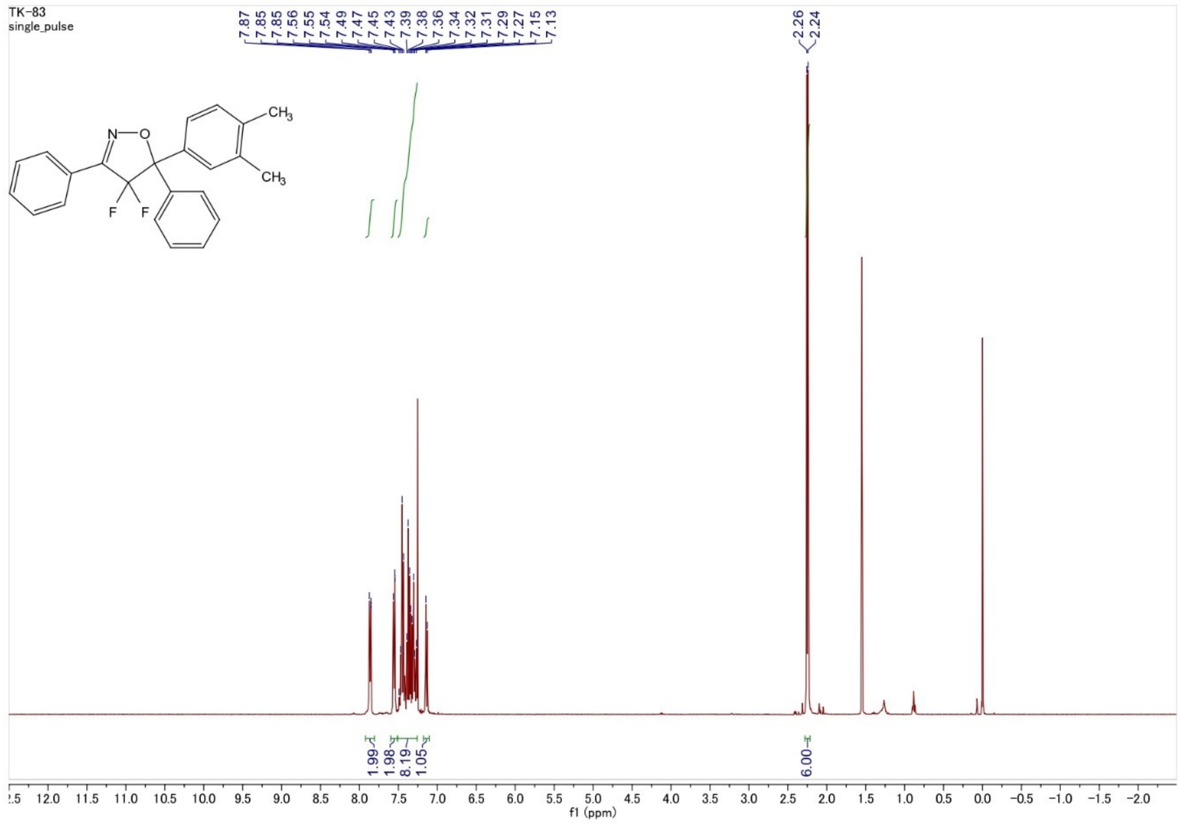

^{19}F NMR of **5aH**

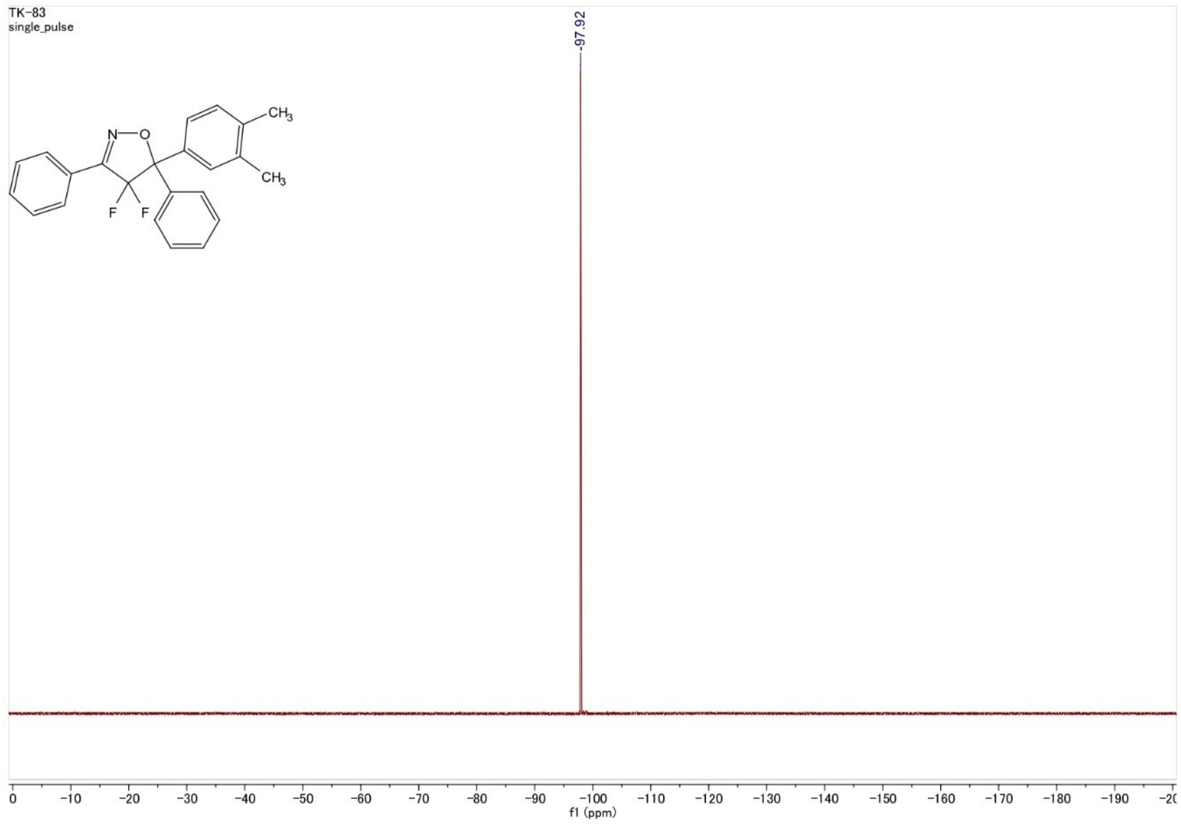

^{13}C NMR of **5aH**

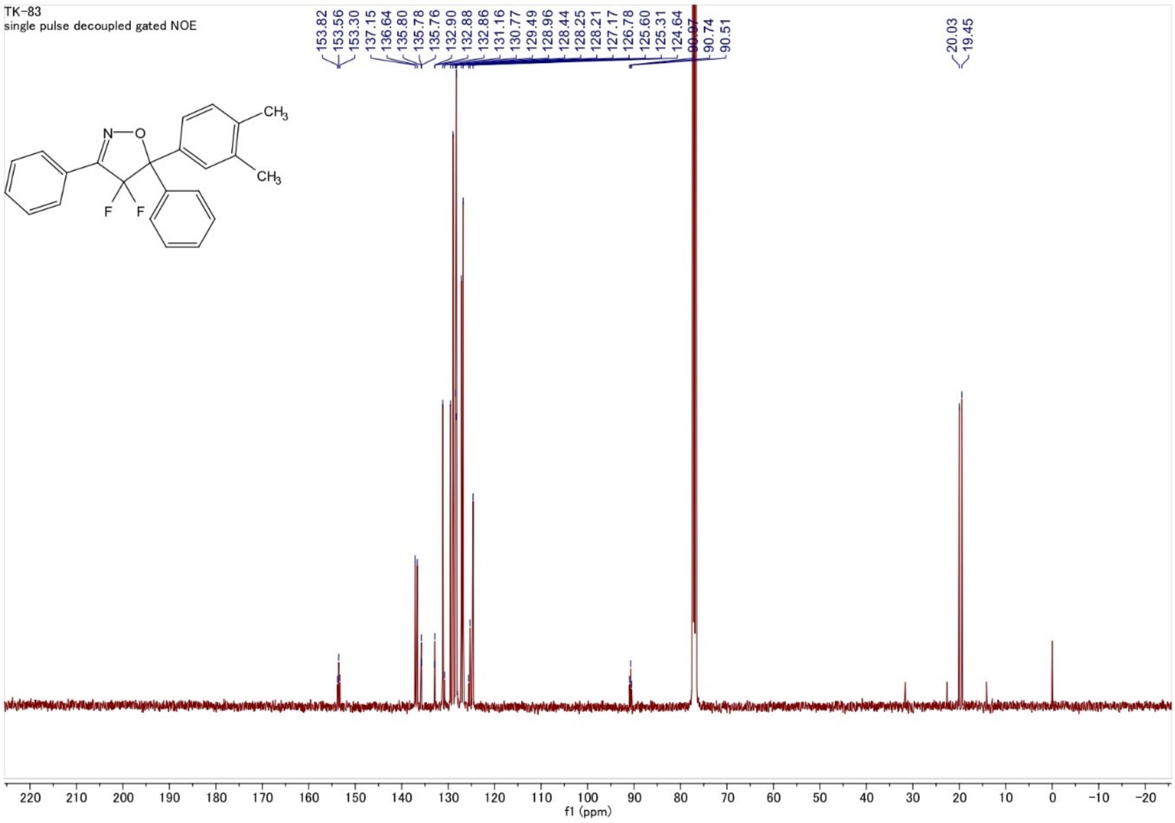

DEPT135 of **5aH**

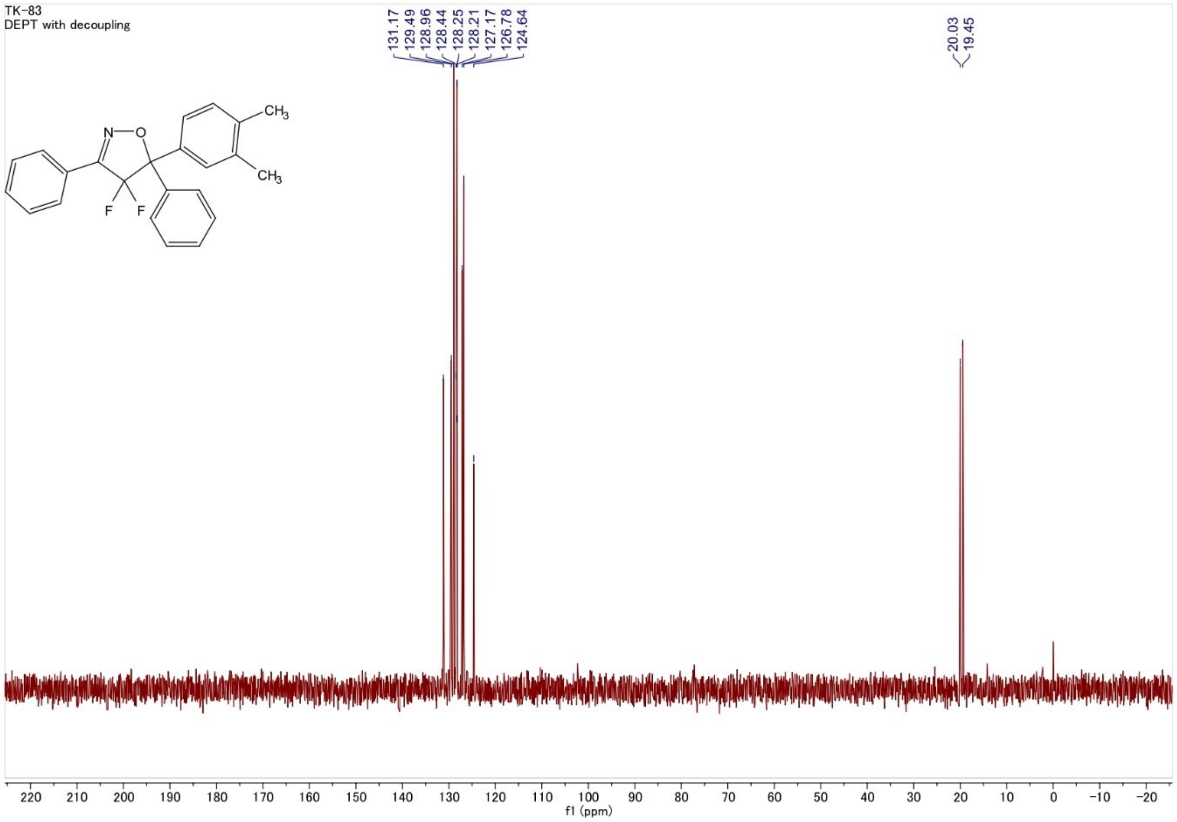

^{1}H NMR of **5al**

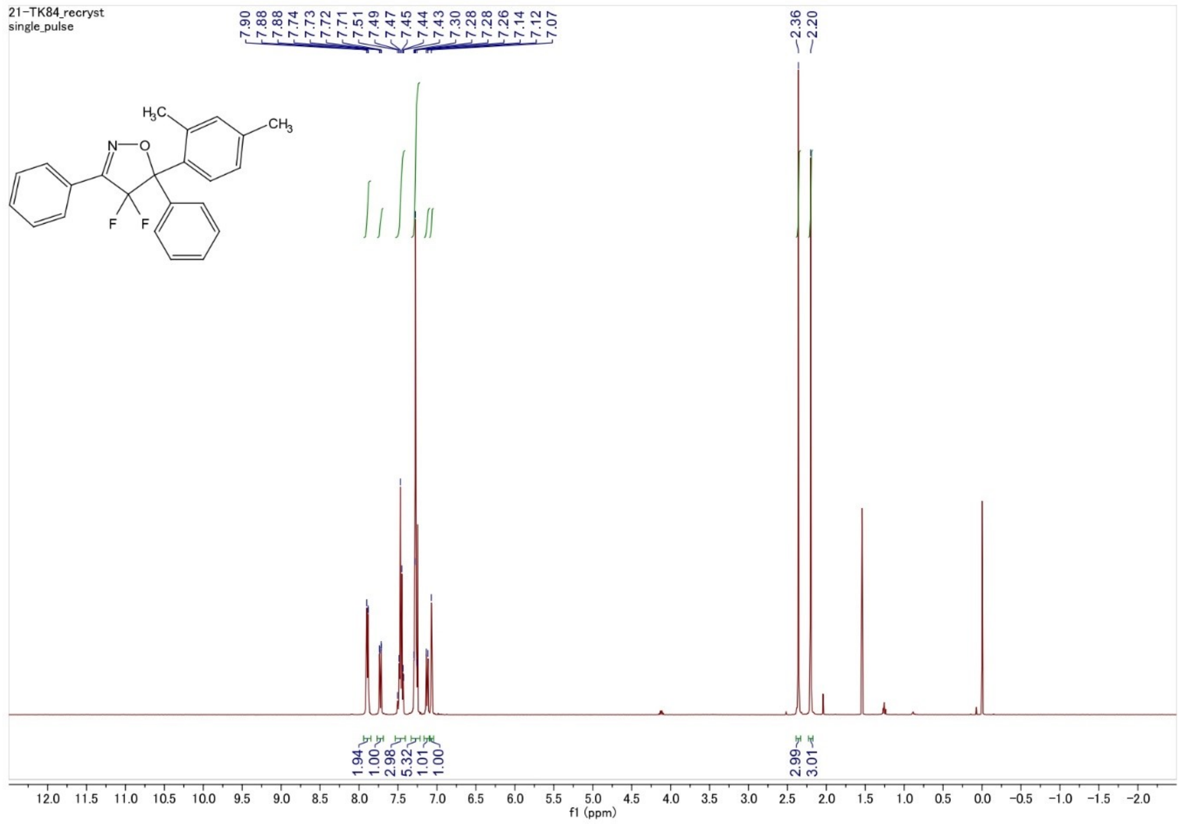

^{19}F NMR of **5al**

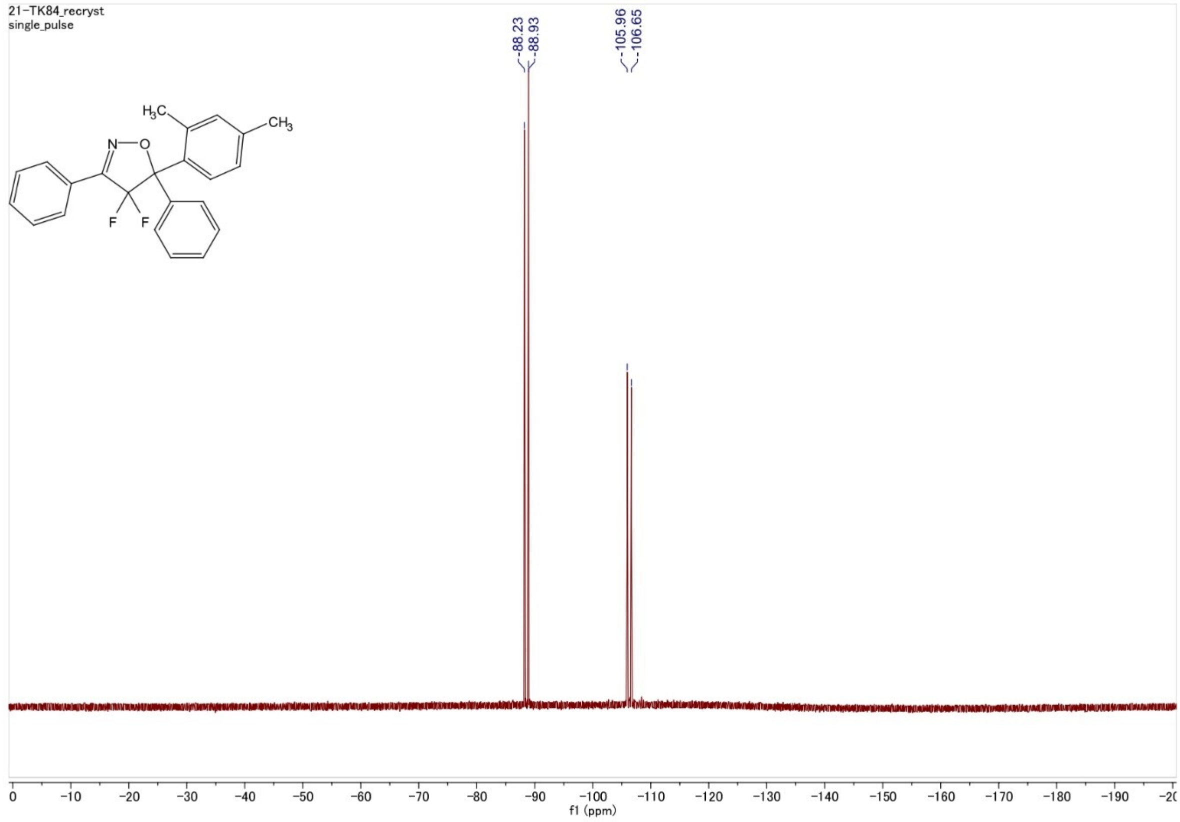

^{13}C NMR of **5al**

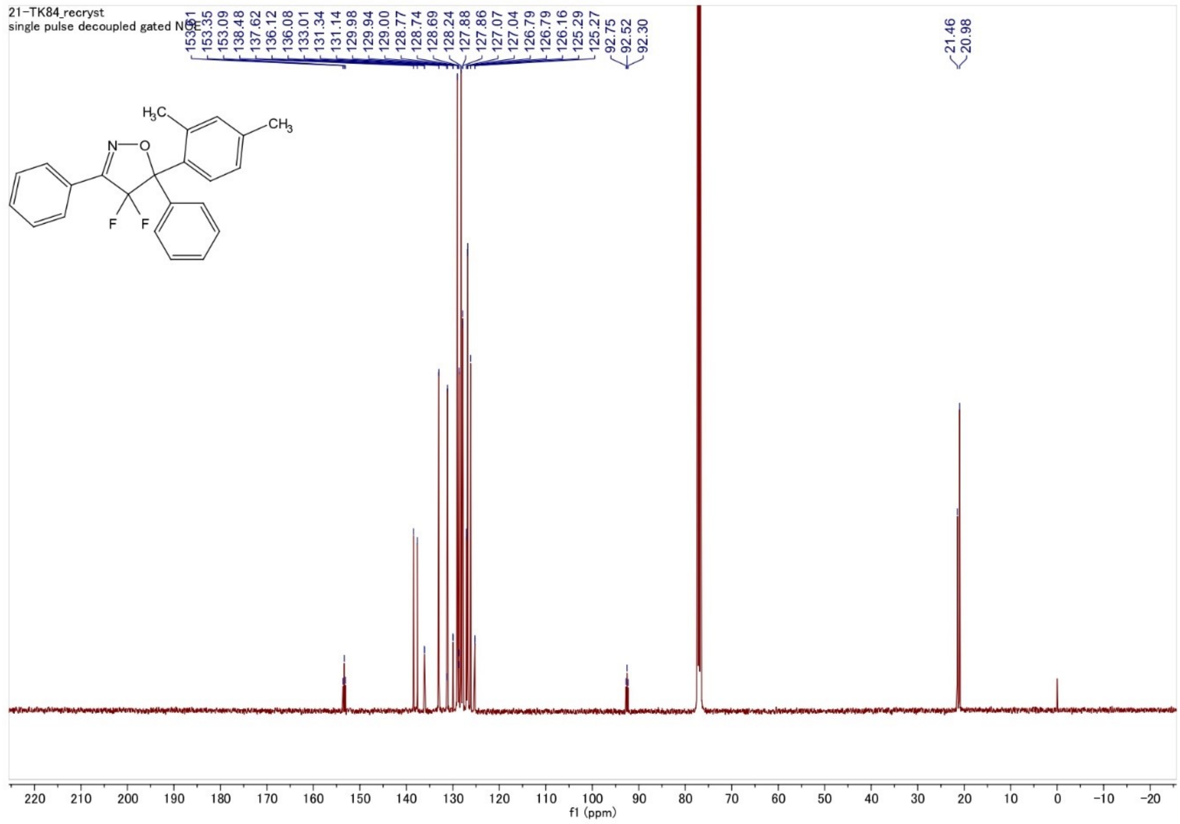

DEPT135 of **5al**

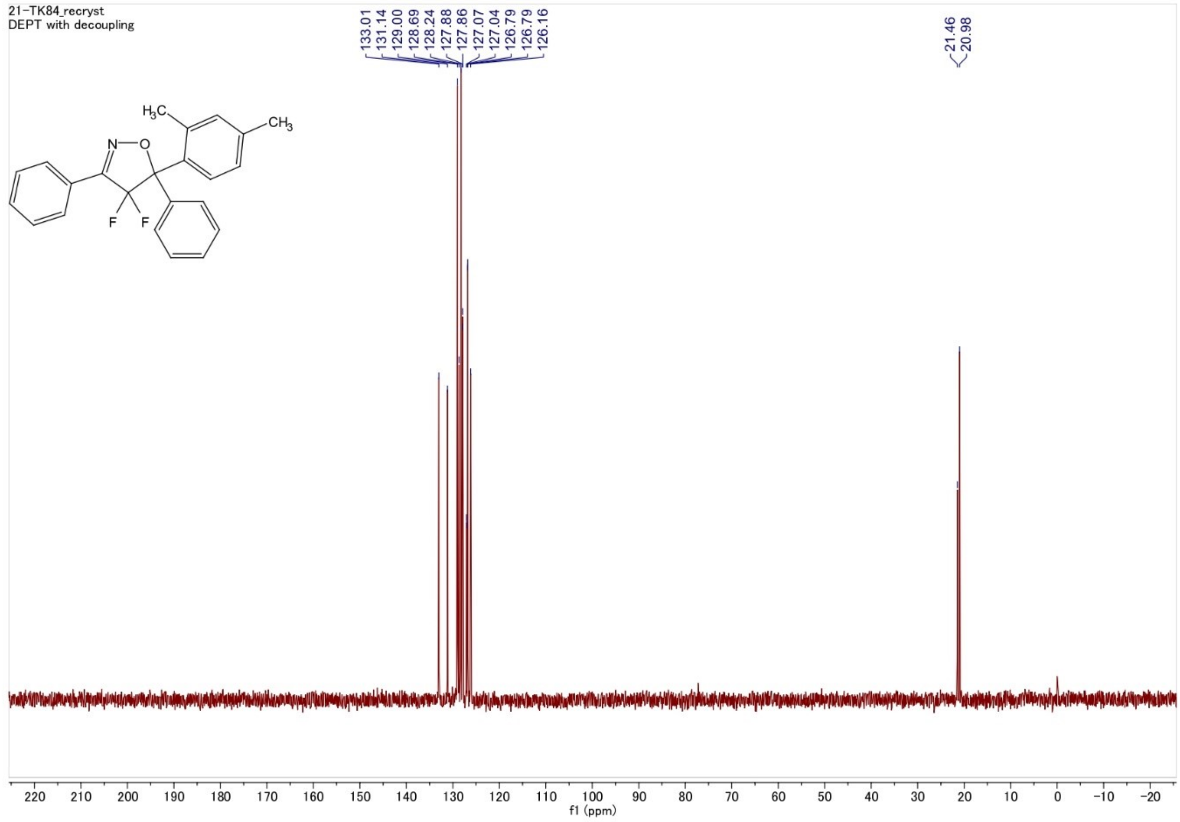

^{1}H NMR of **5aJ**

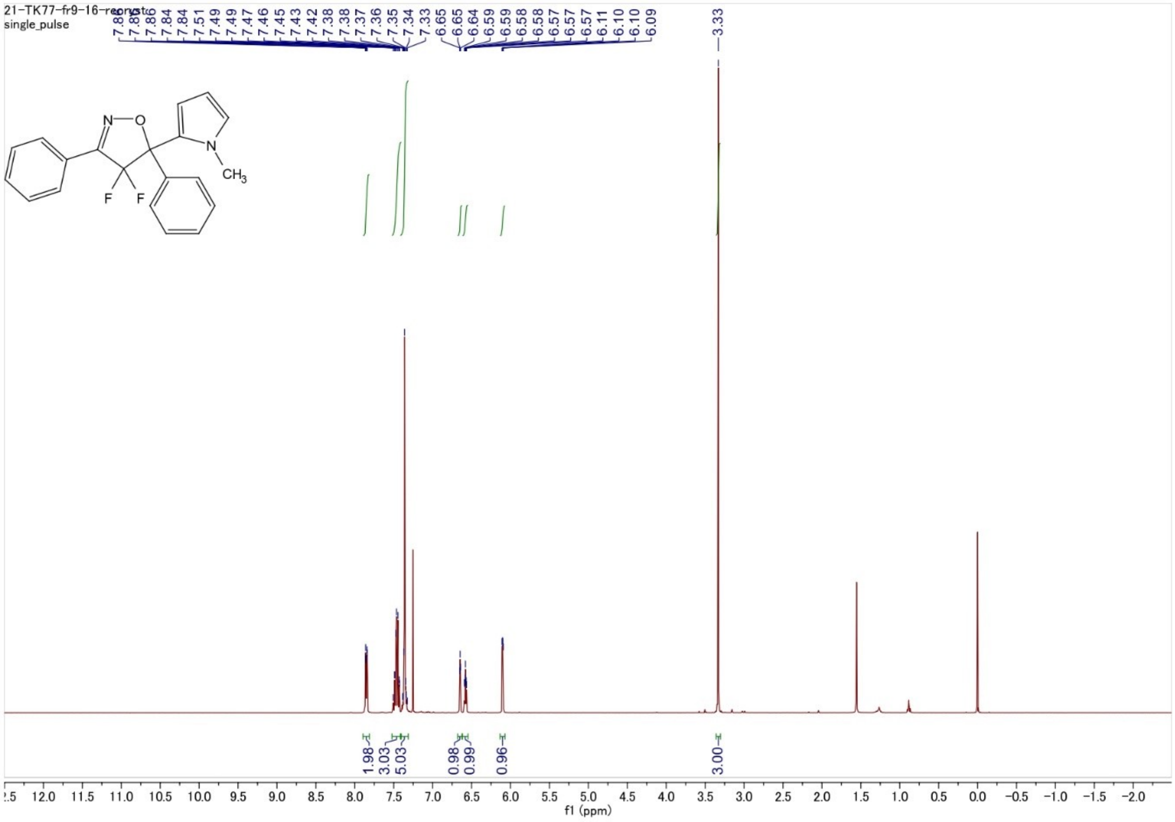

^{19}F NMR of **5aJ**

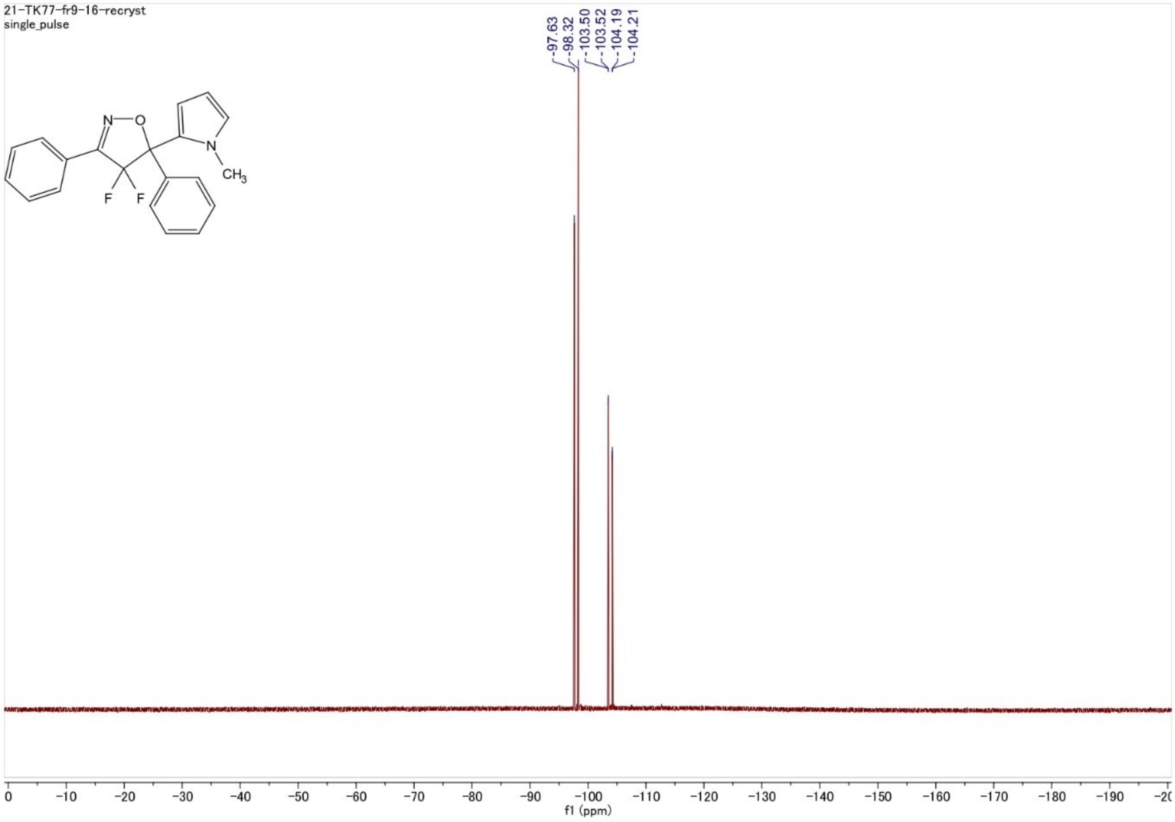

^{13}C NMR of **5aJ**

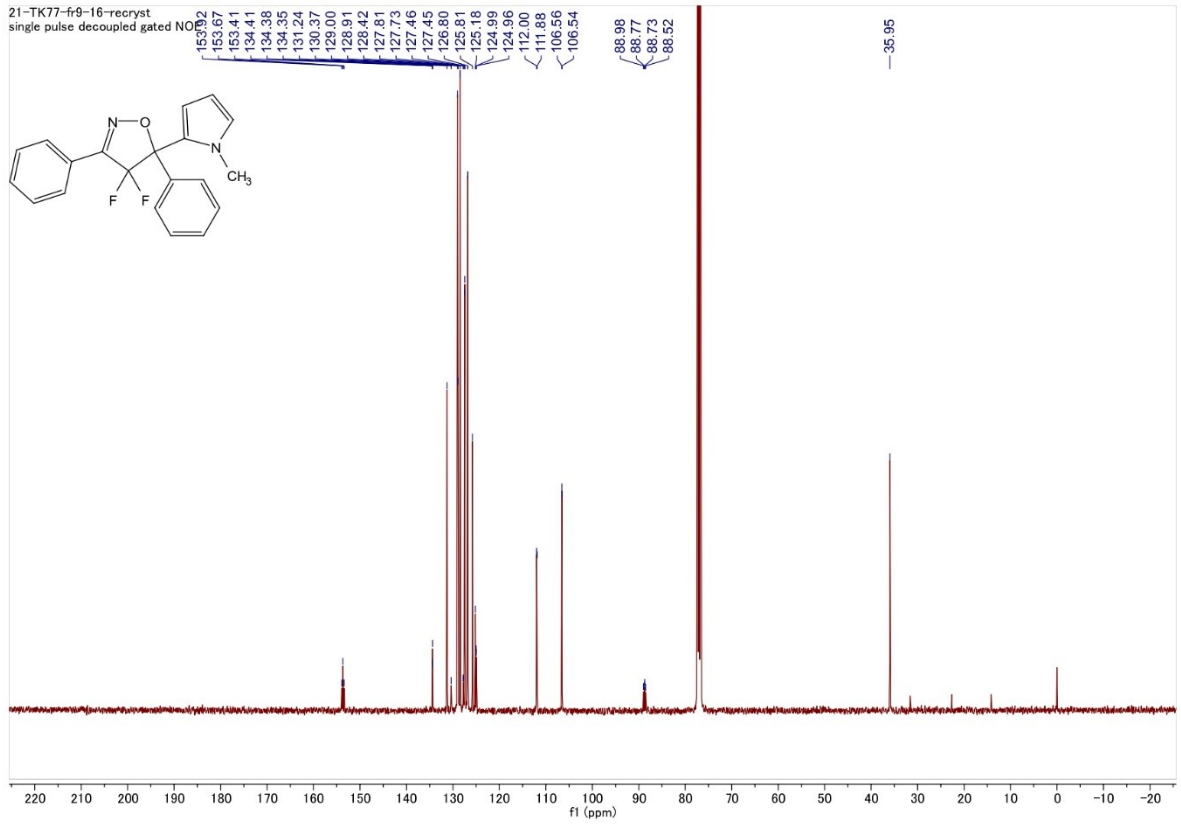

DEPT135 of **5aJ**

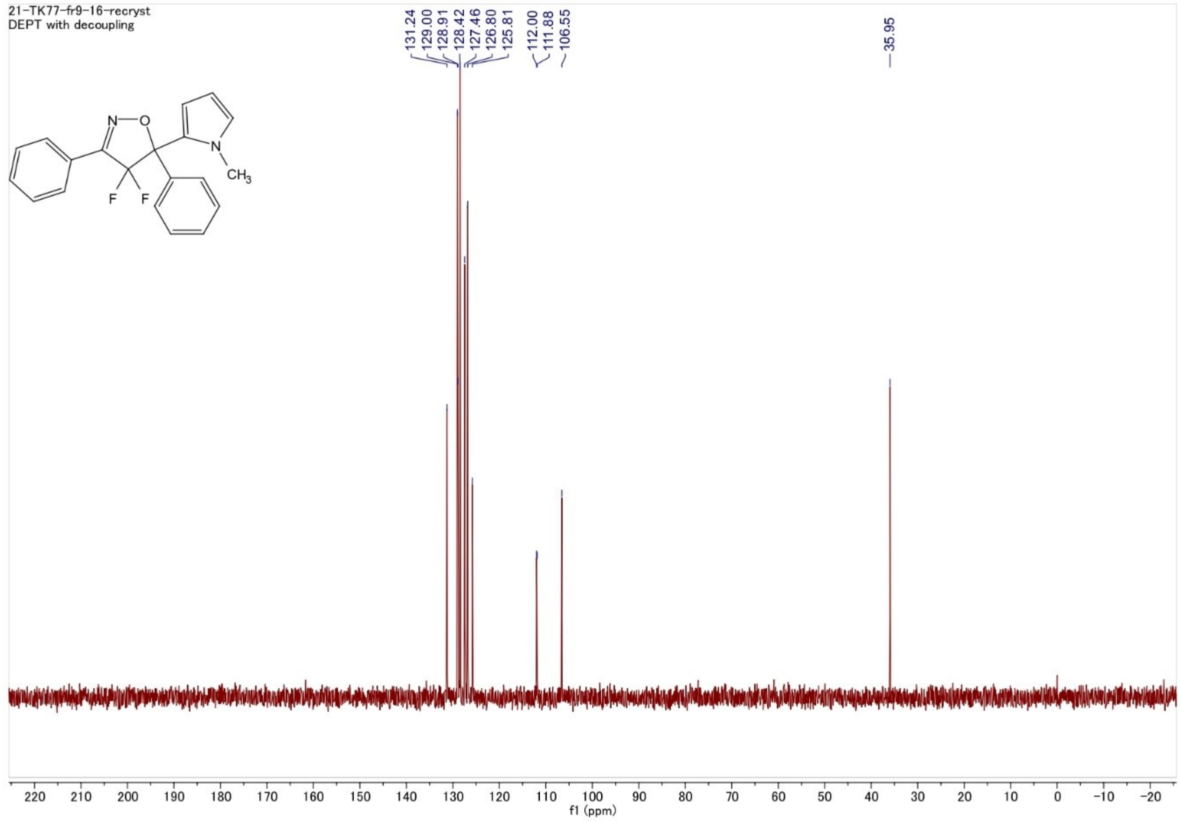

^{1}H NMR of **5aJ'**

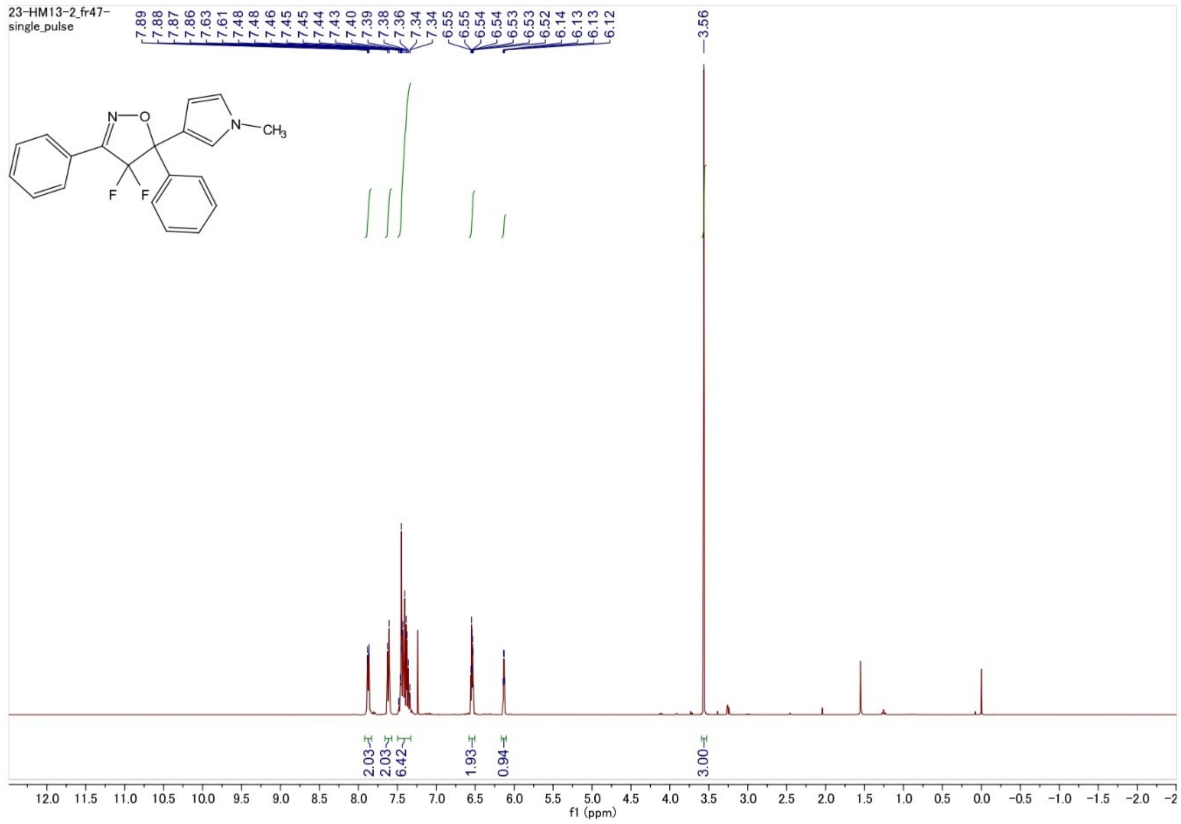

^{19}F NMR of **5aJ'**

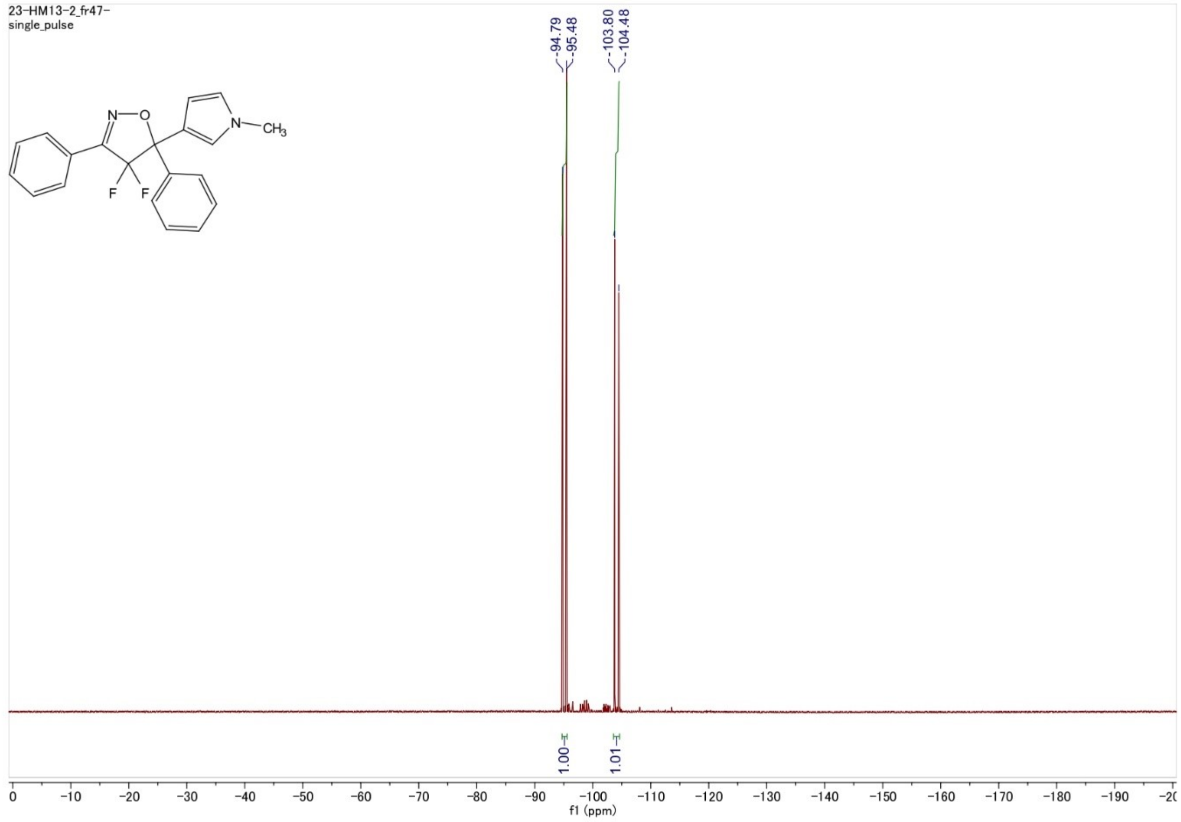

^{13}C NMR of **5aJ'**

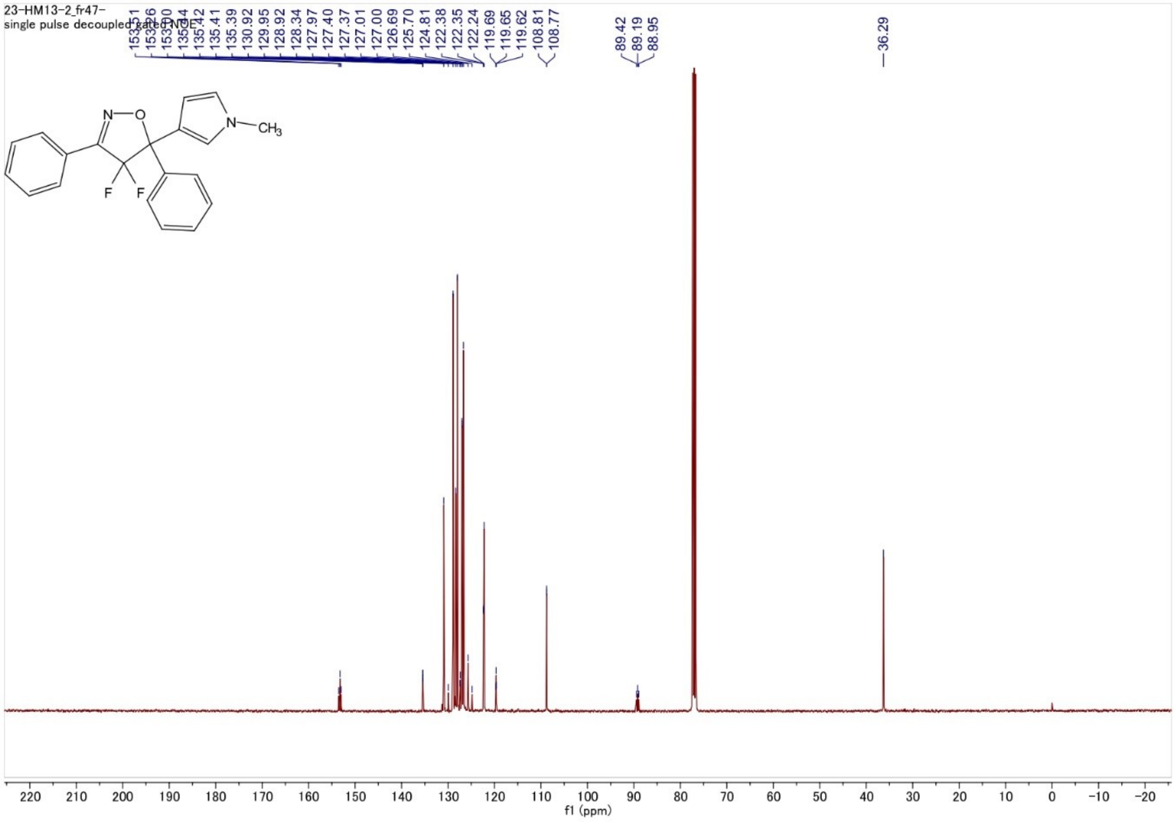

DEPT135 of **5aJ'**

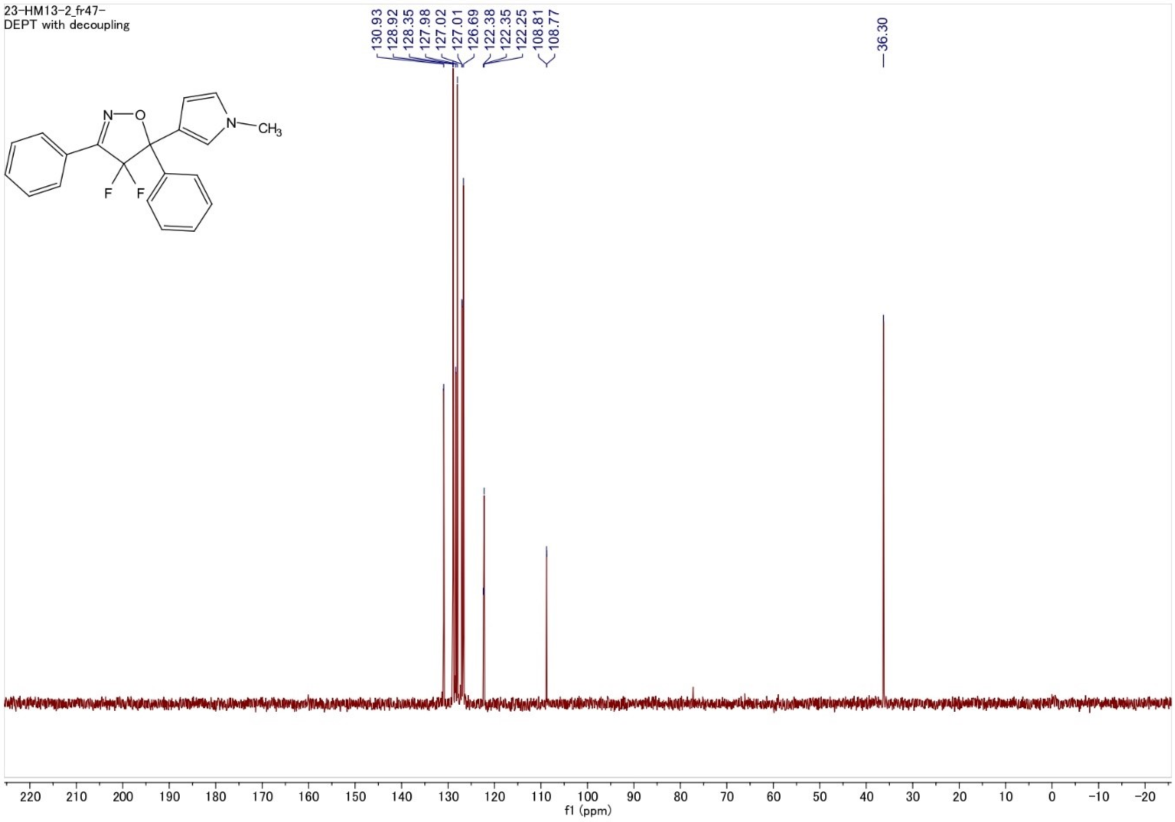

^{1}H NMR of **5aK**

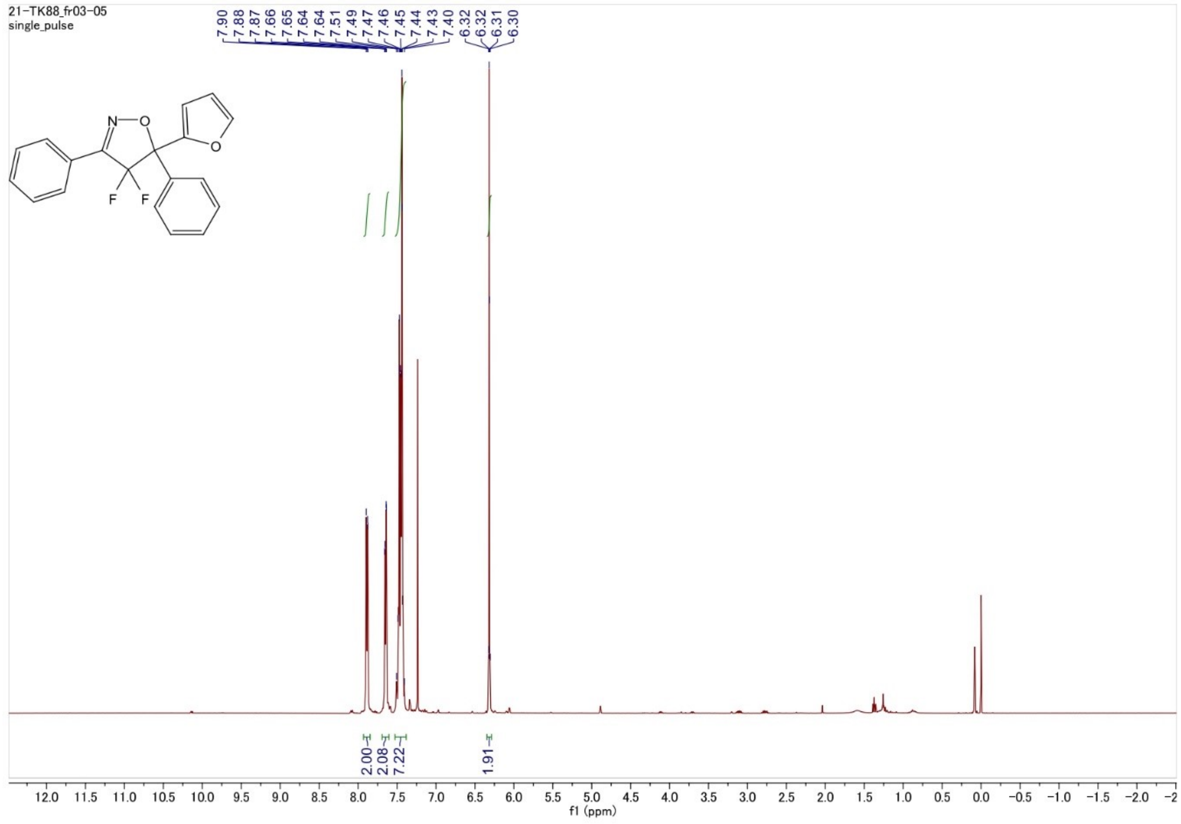

^{19}F NMR of **5aK**

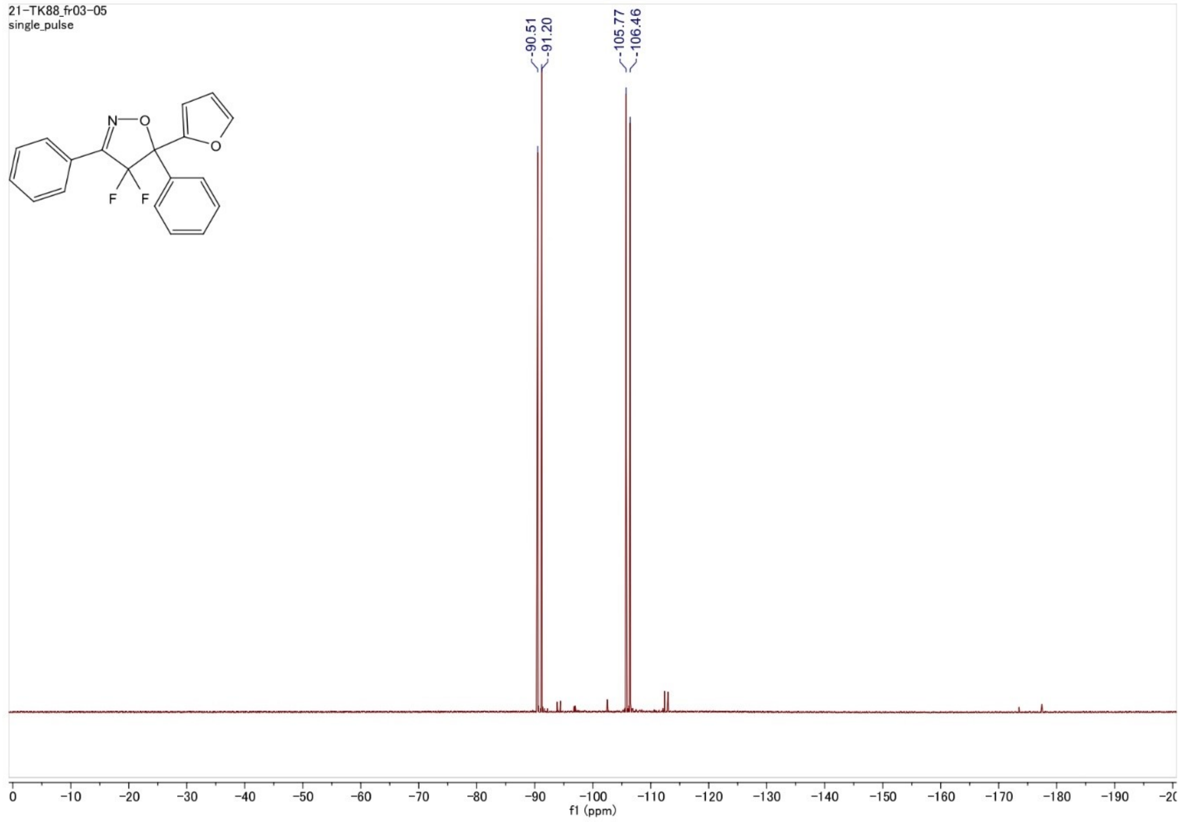

^{13}C NMR of **5aK**

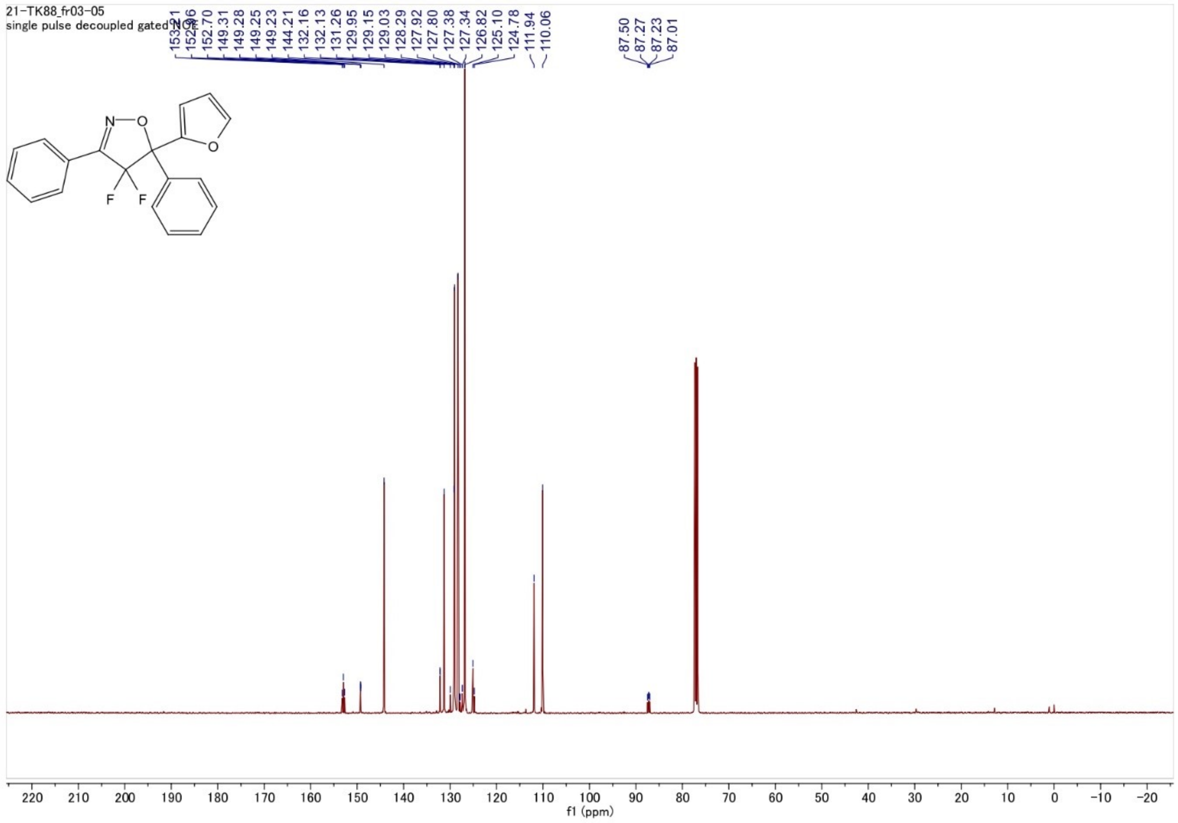

DEPT135 of **5aK**

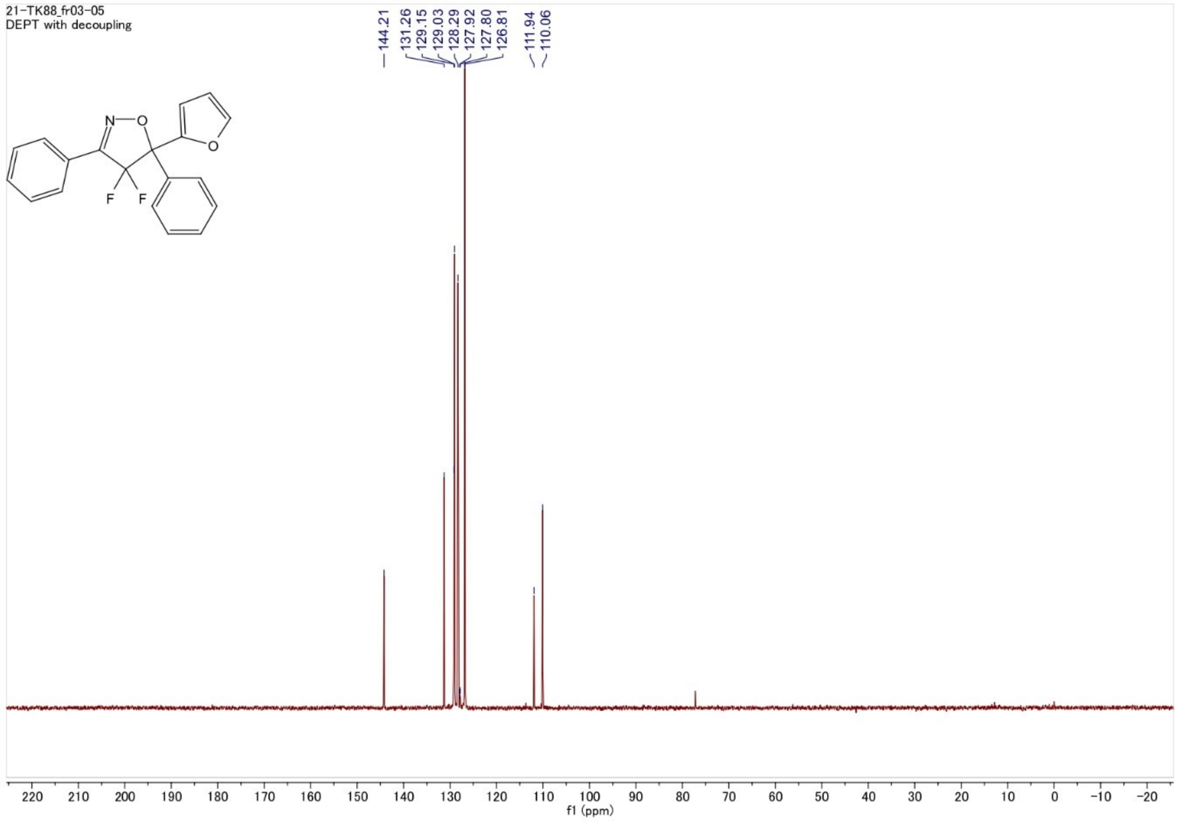

^{1}H NMR of **5aL**

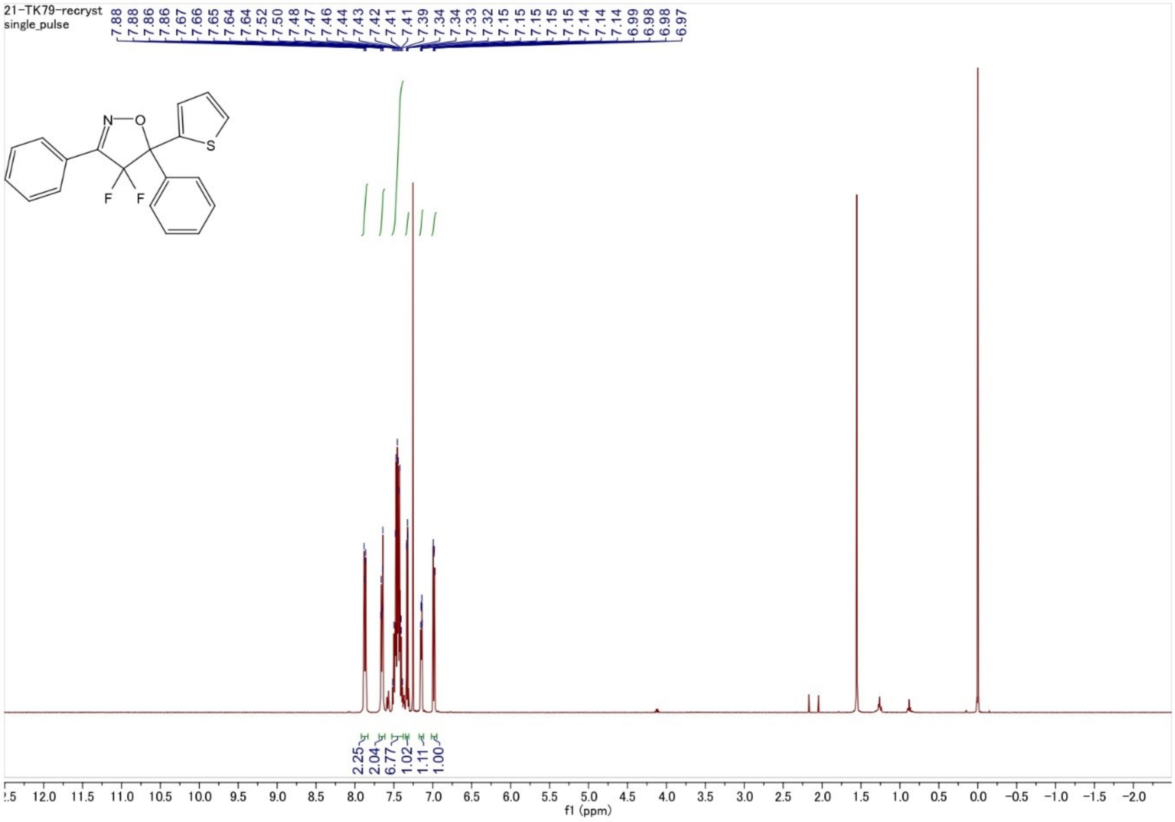

^{19}F NMR of **5aL**

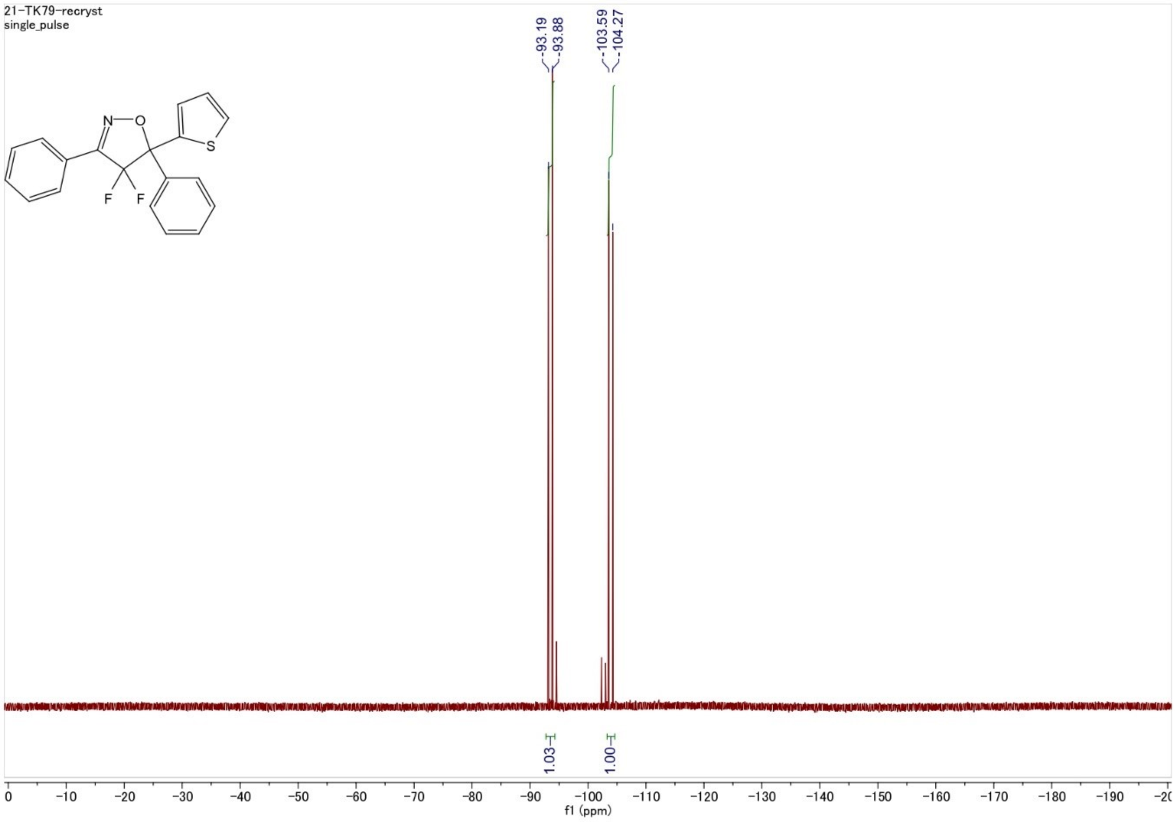

^{13}C NMR of **5aL**

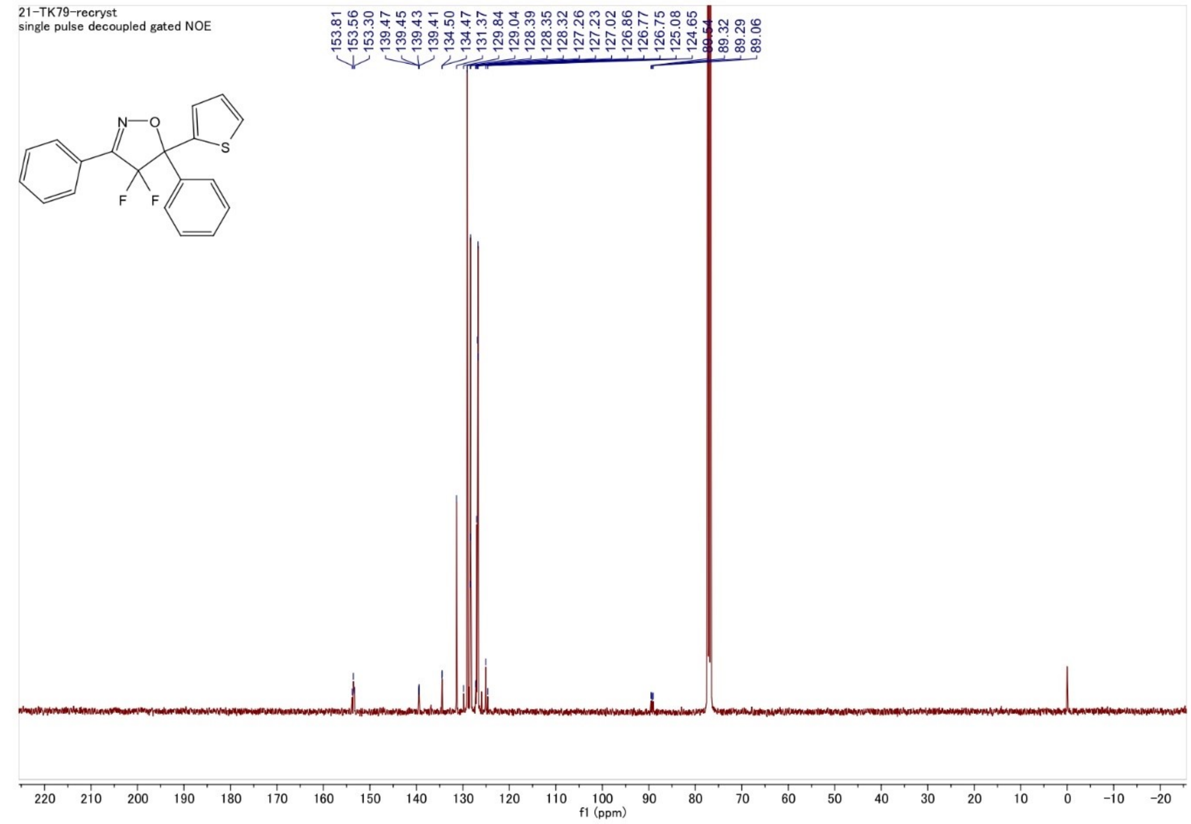

DEPT135 of **5aL**

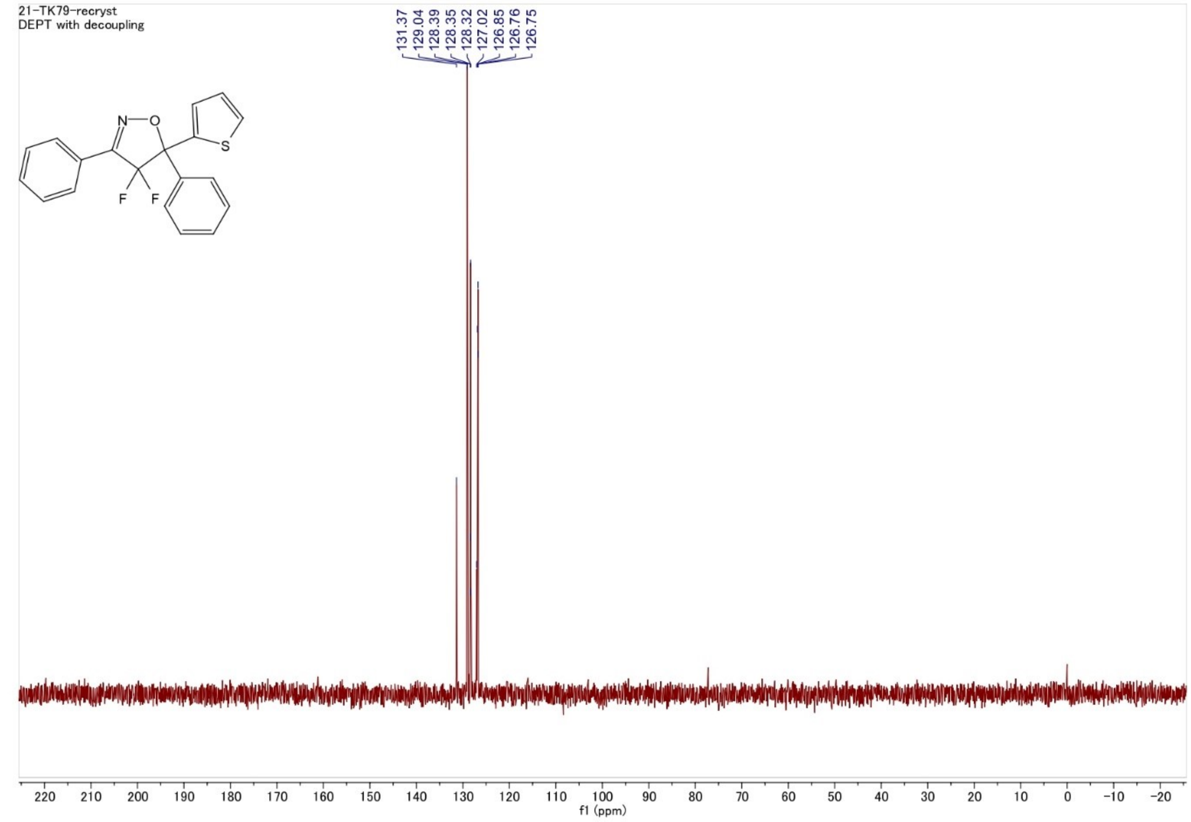

^{1}H NMR of **5bB**

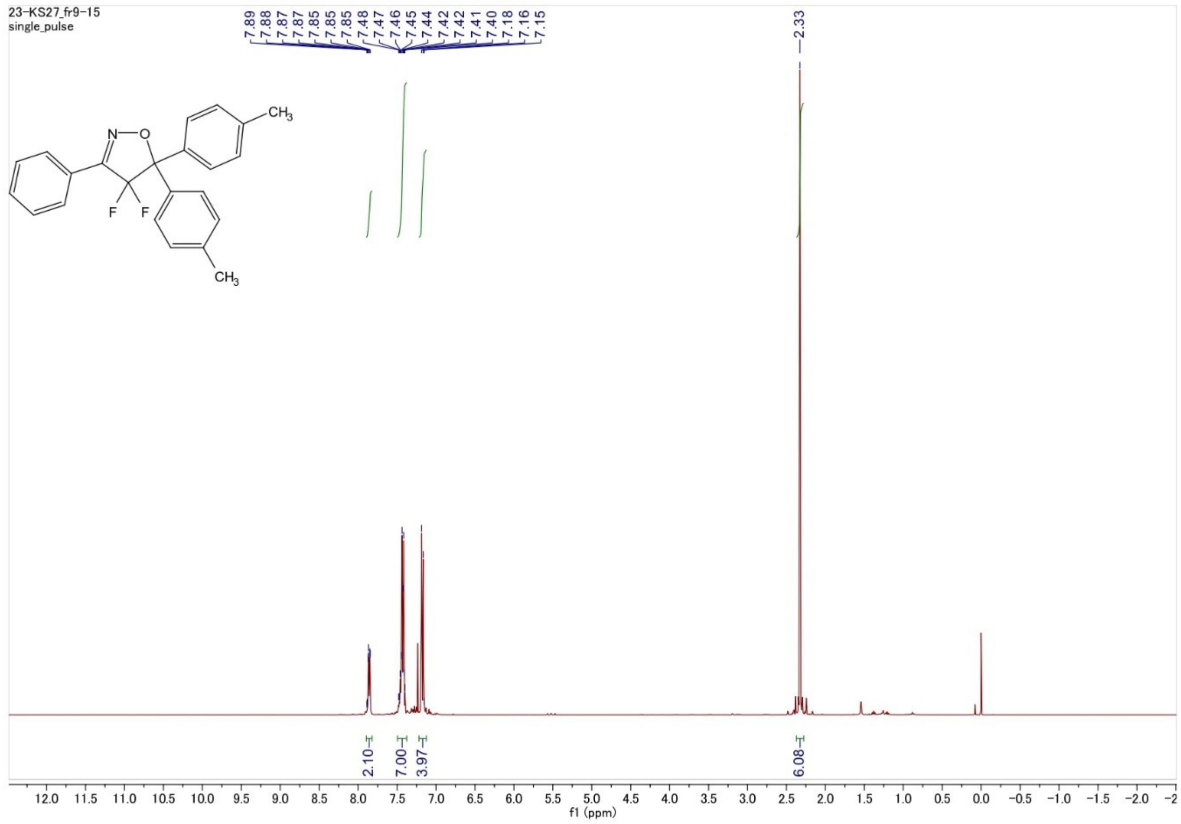

^{19}F NMR of **5bB**

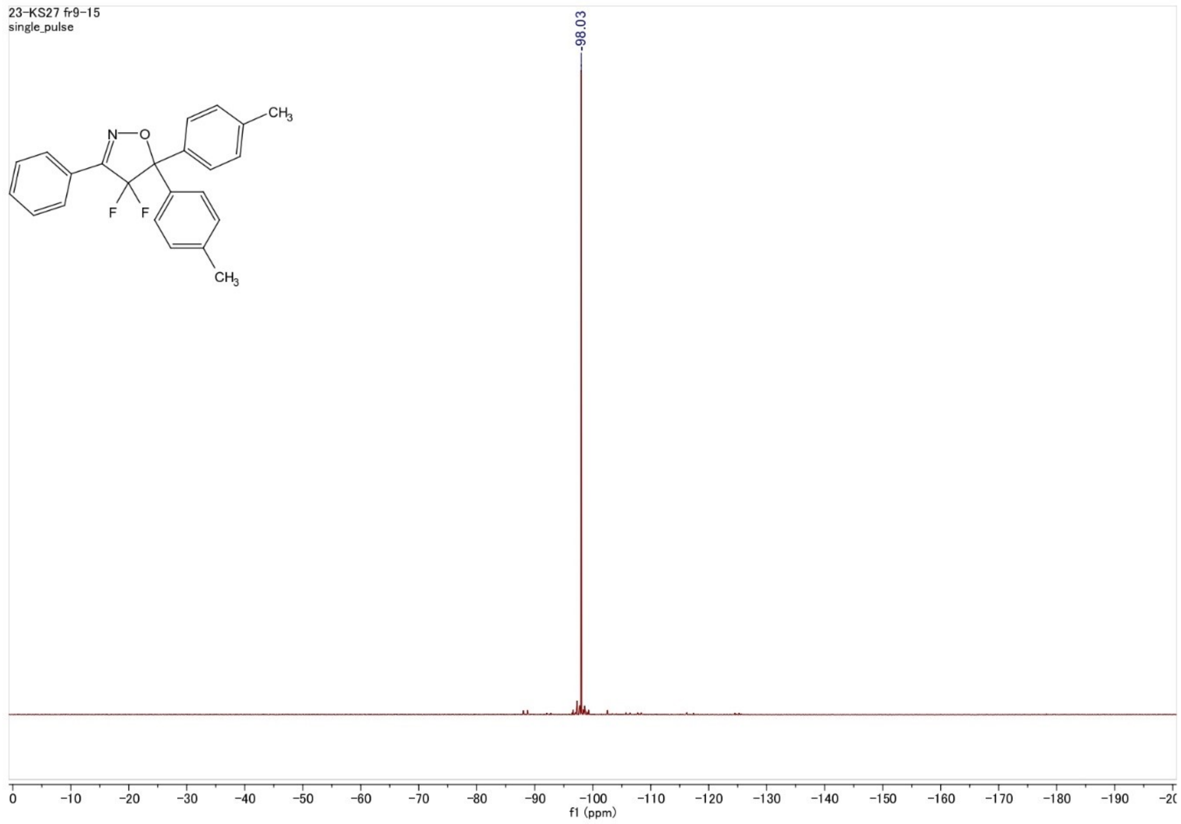

^{13}C NMR of **5bB**

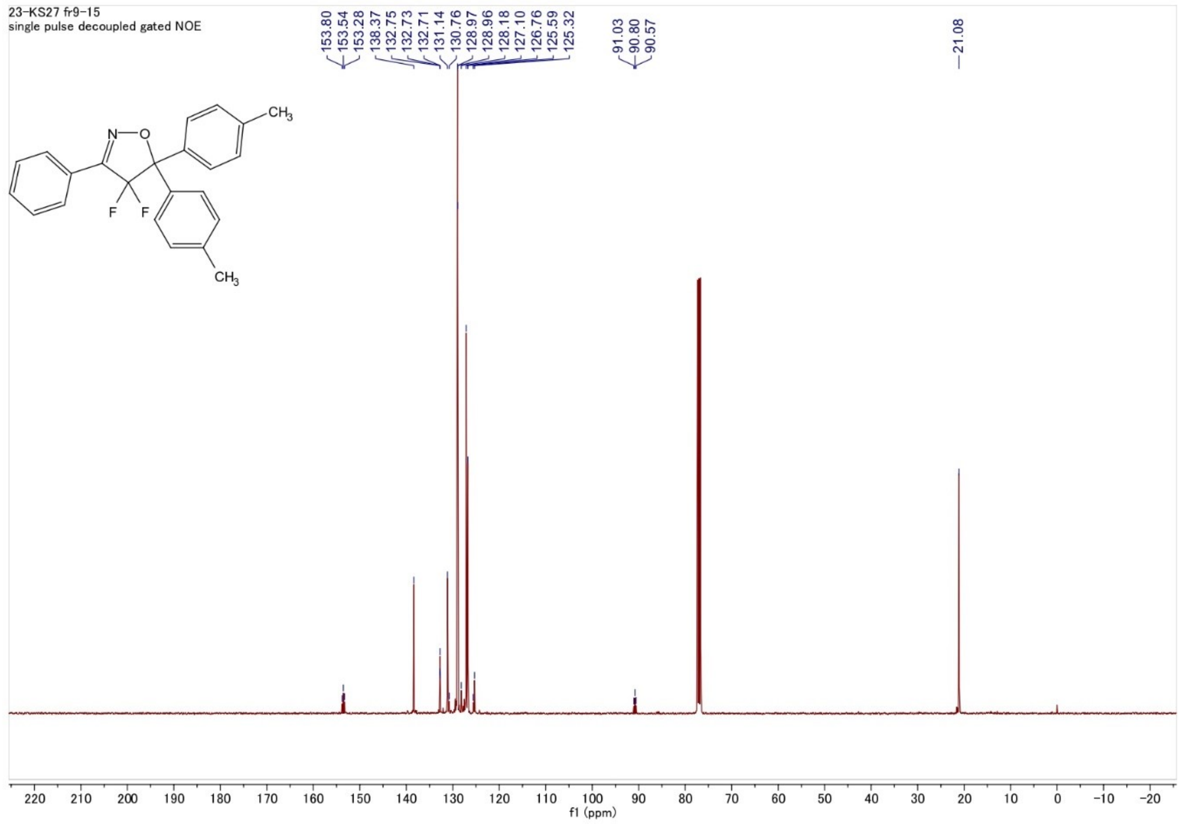

DEPT135 of **5bB**

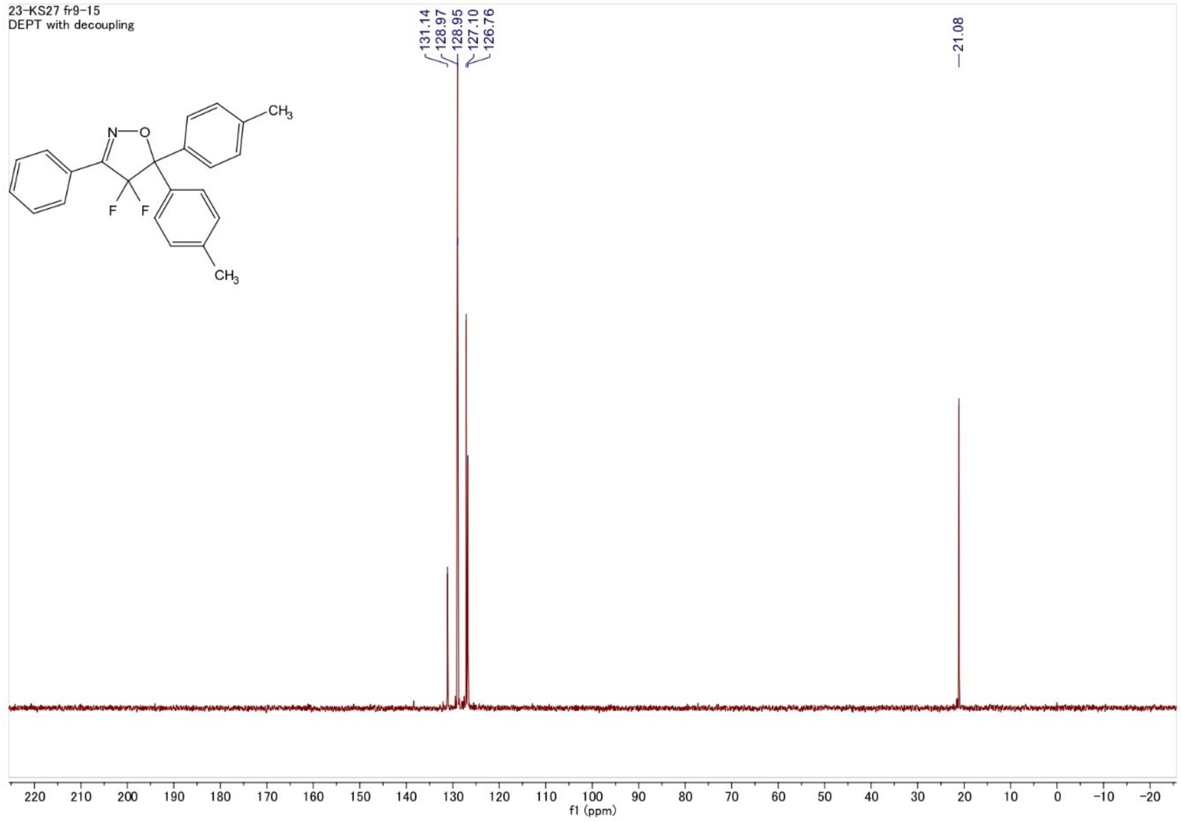

^{1}H NMR of **5cB**

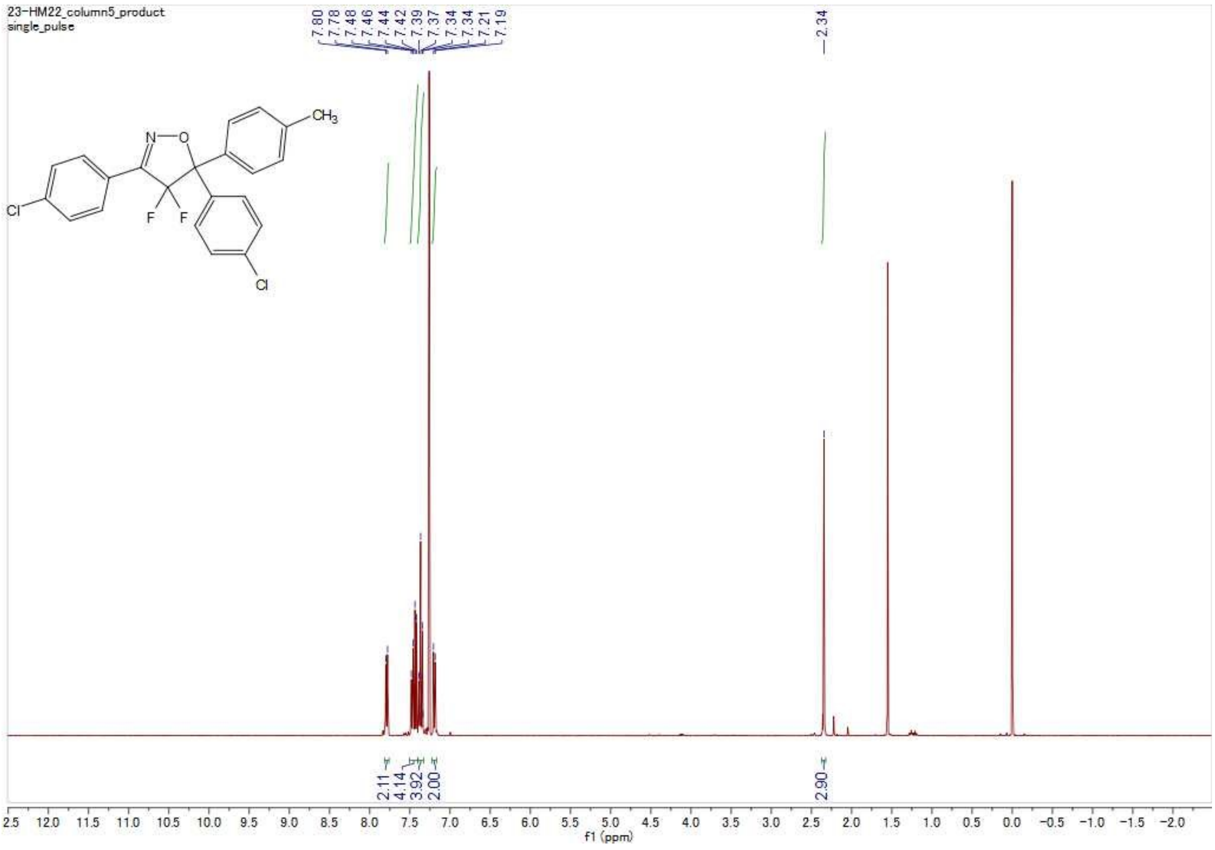

^{19}F NMR of **5cB**

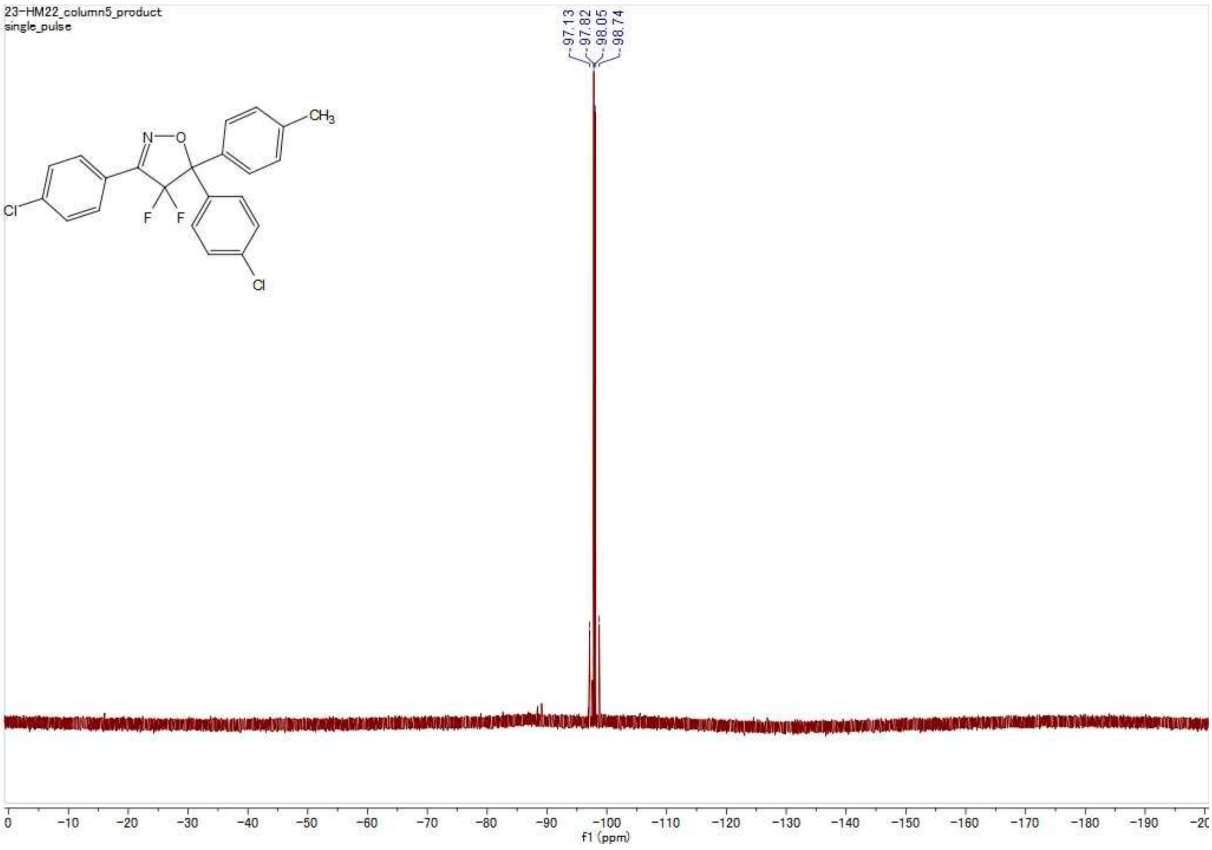

^{13}C NMR of **5cB**

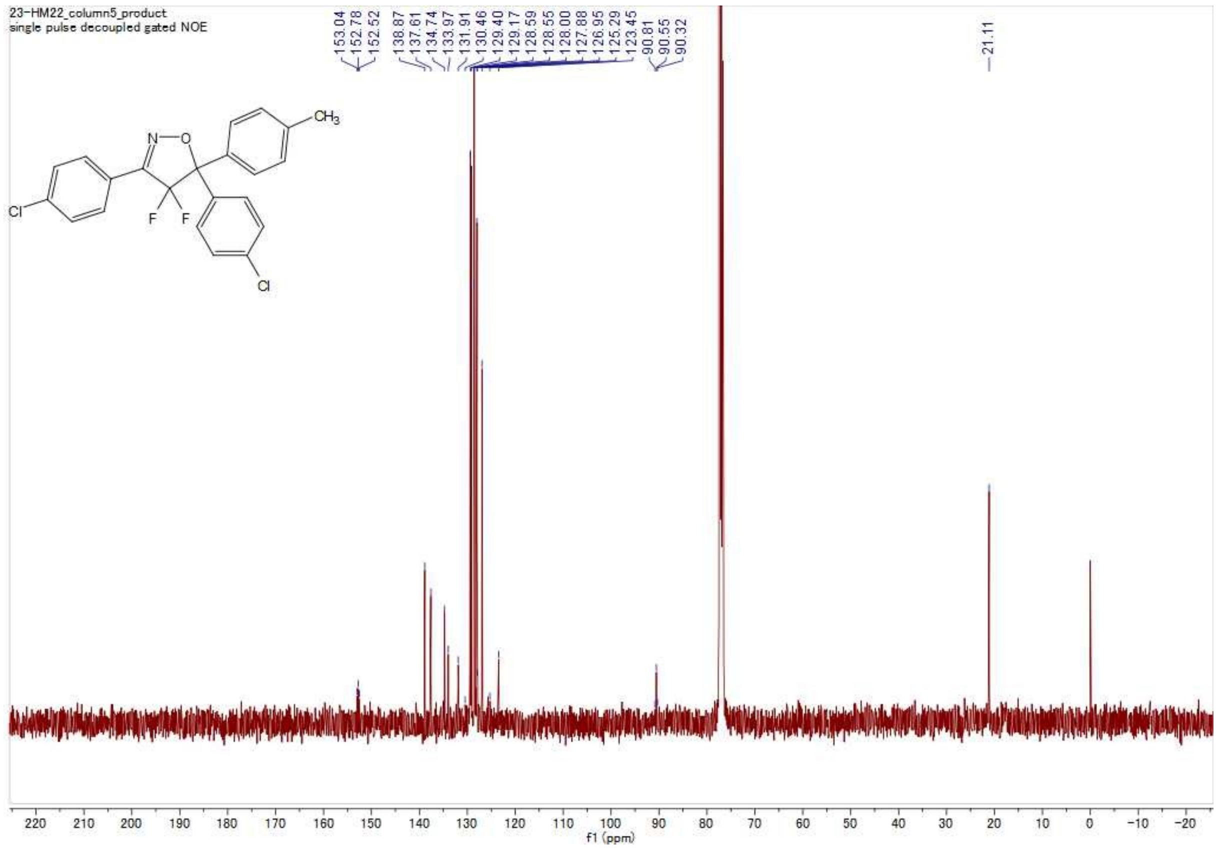

DEPT135 of **5cB**

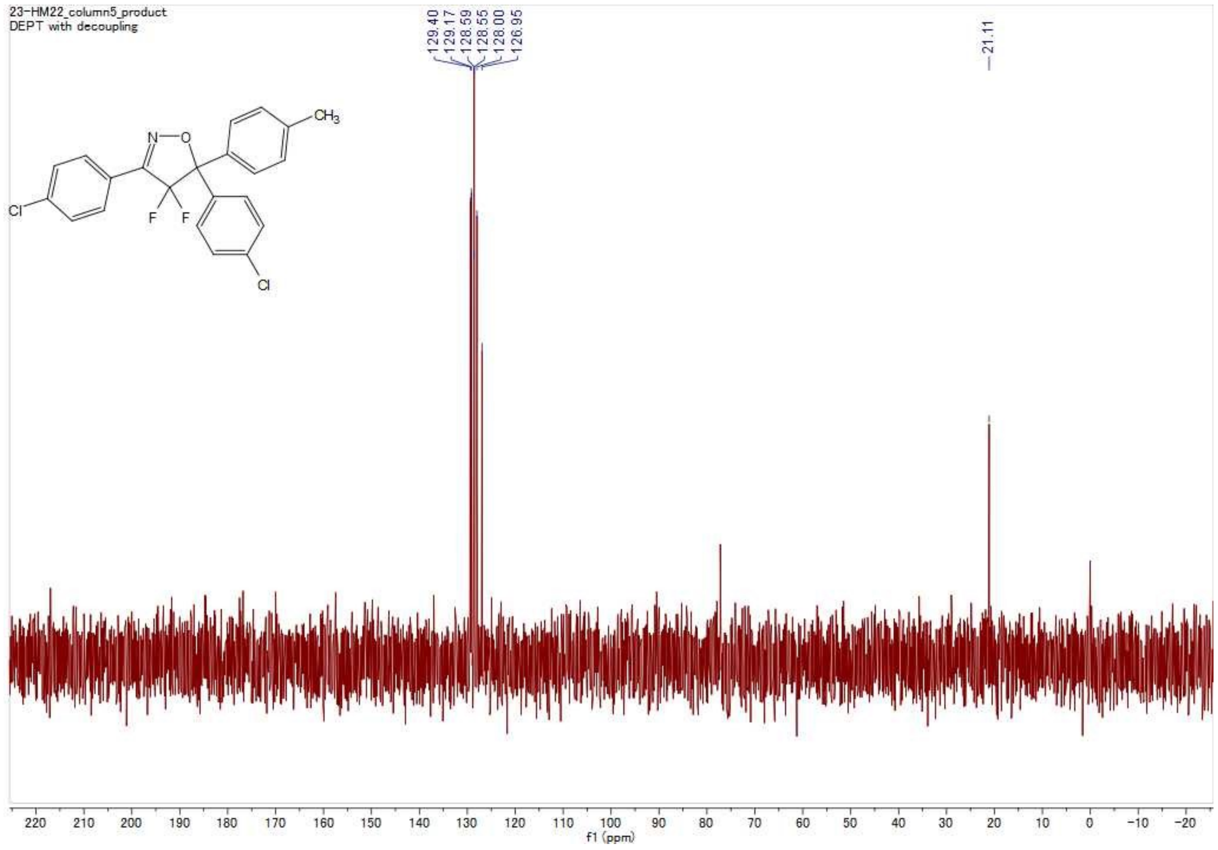

^{1}H NMR of **5dB**

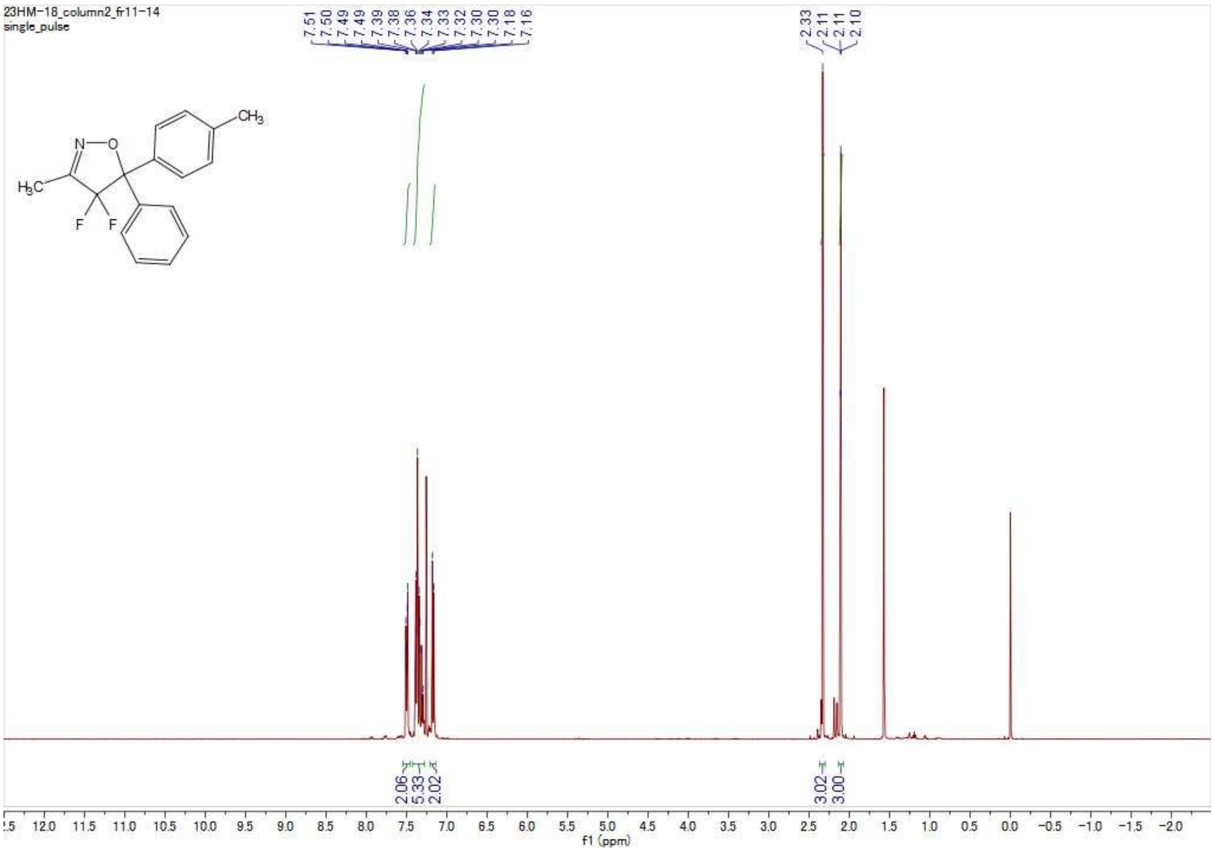

^{19}F NMR of **5dB**

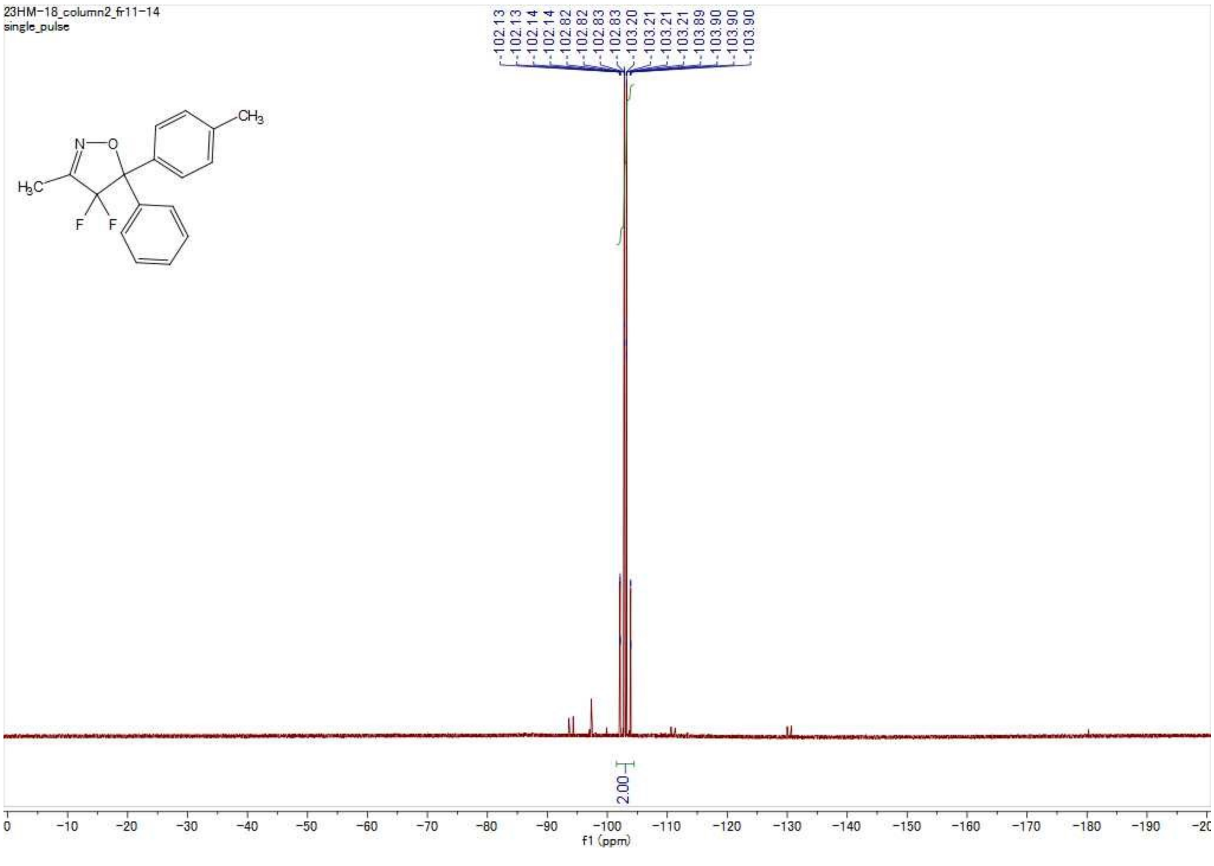

^{13}C NMR of **5dB**

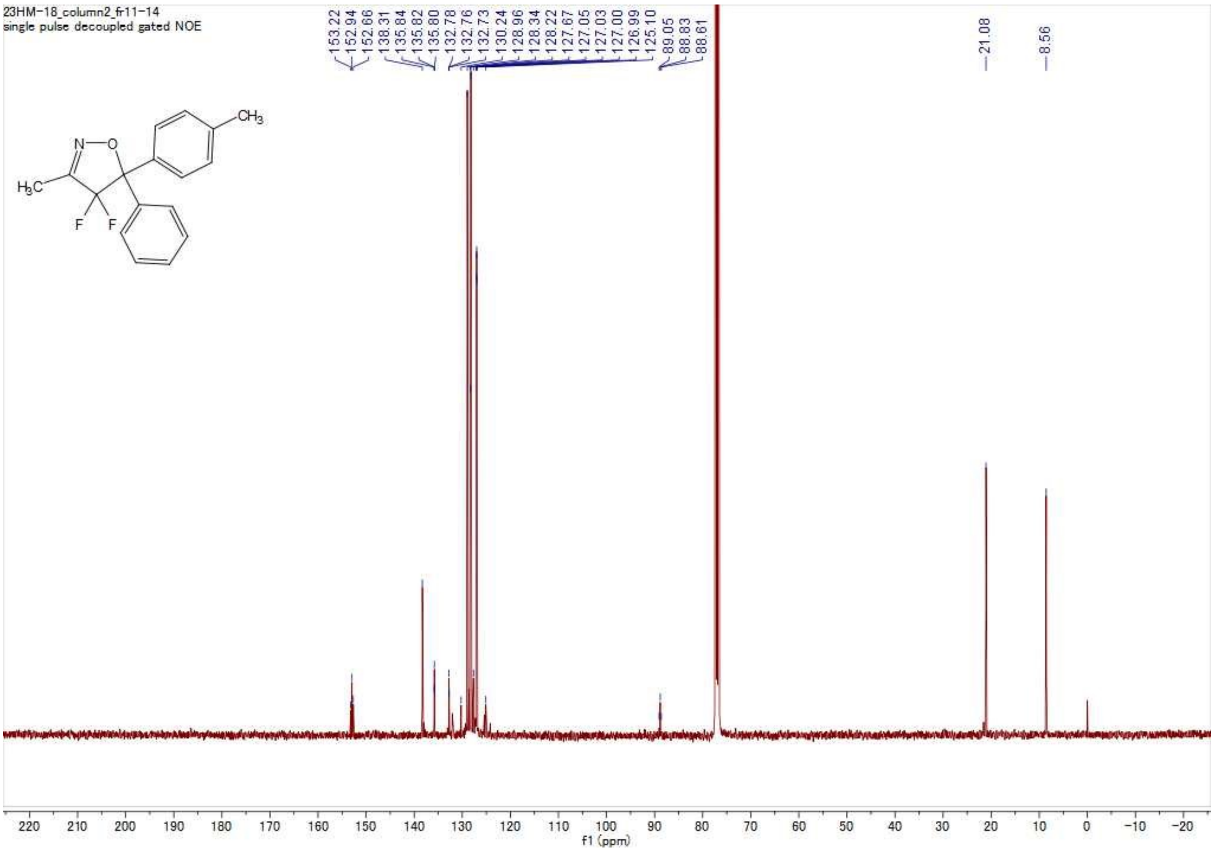

DEPT135 of **5dB**

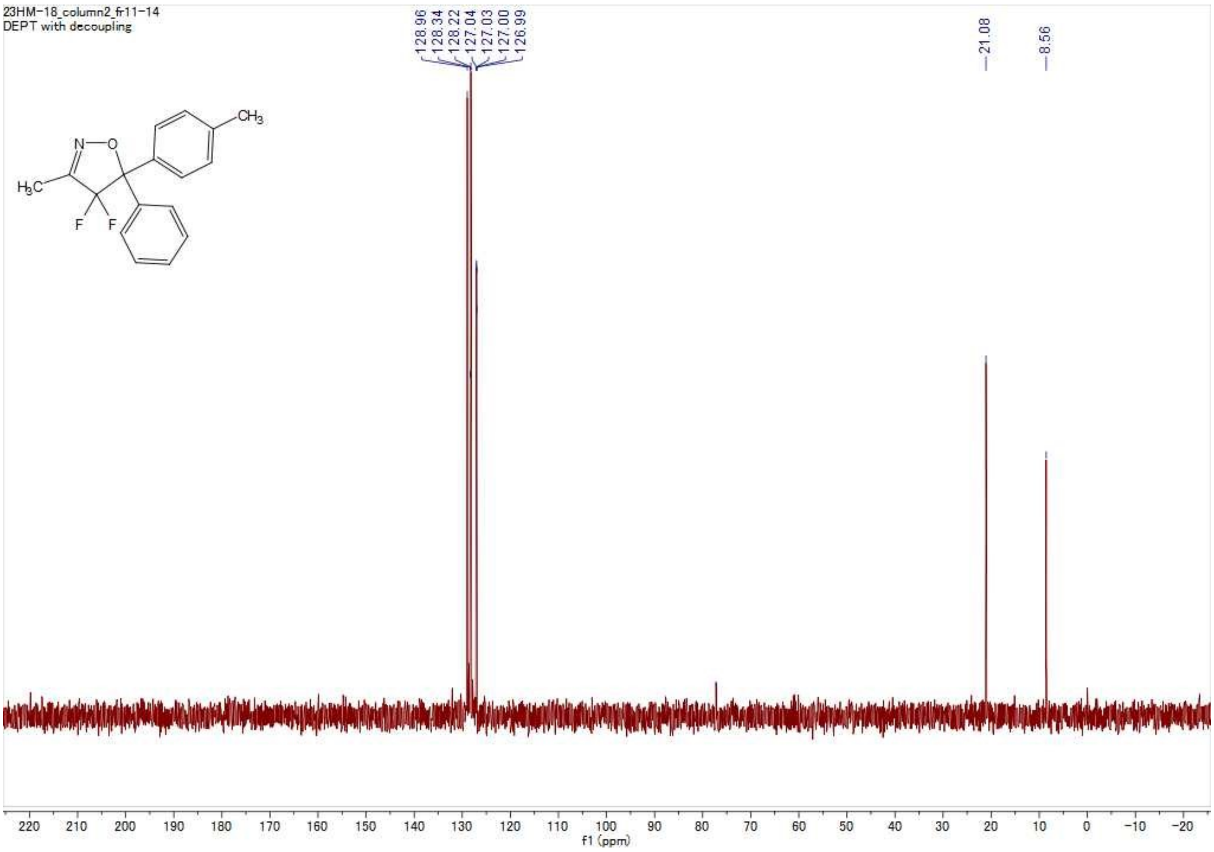

^{1}H NMR of **6**

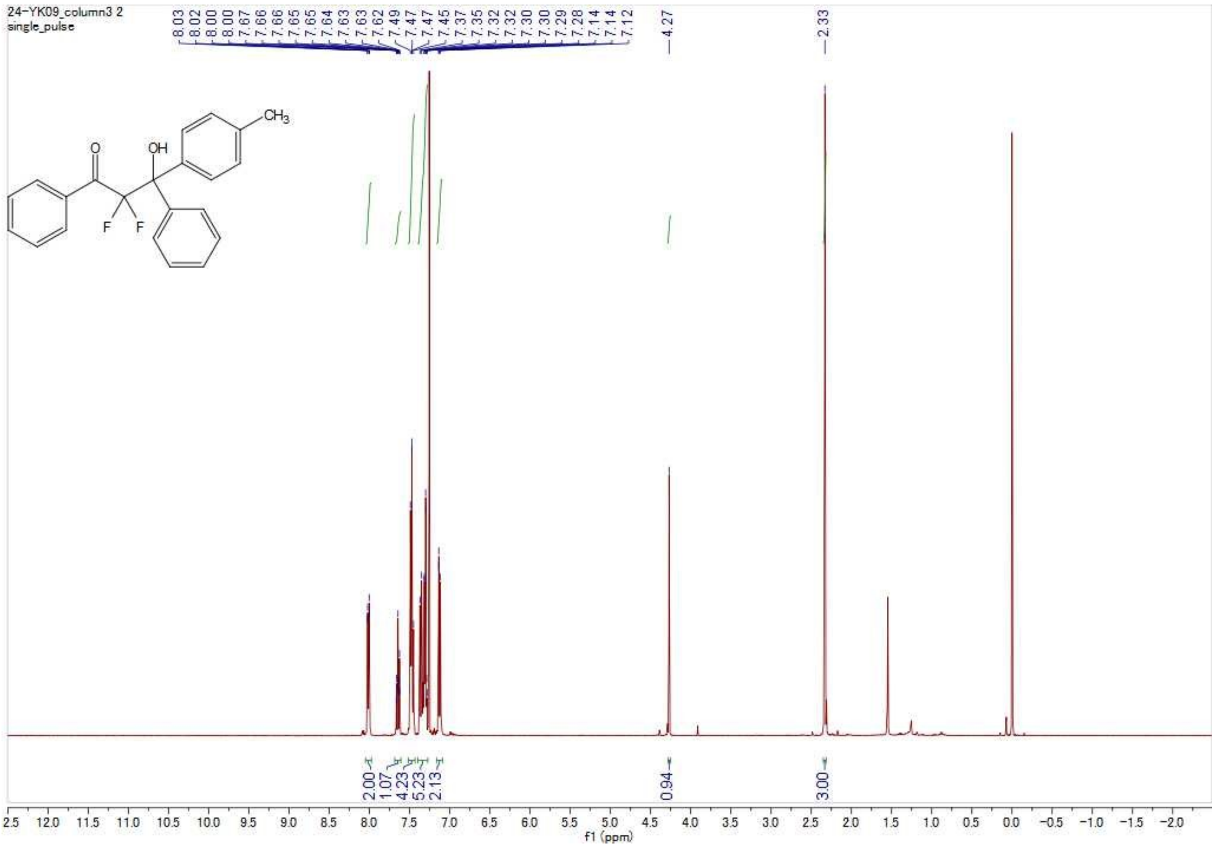

^{19}F NMR of **6**

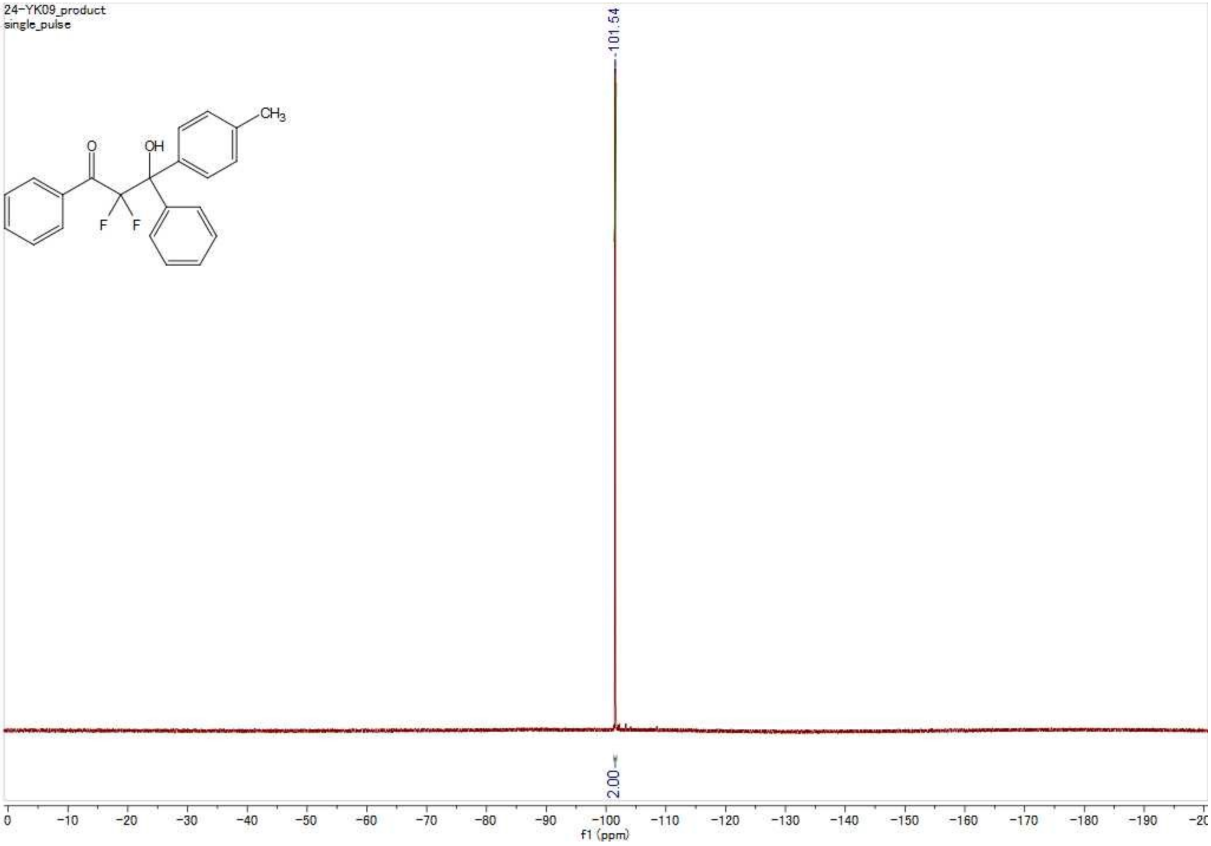

^{13}C NMR of **6**

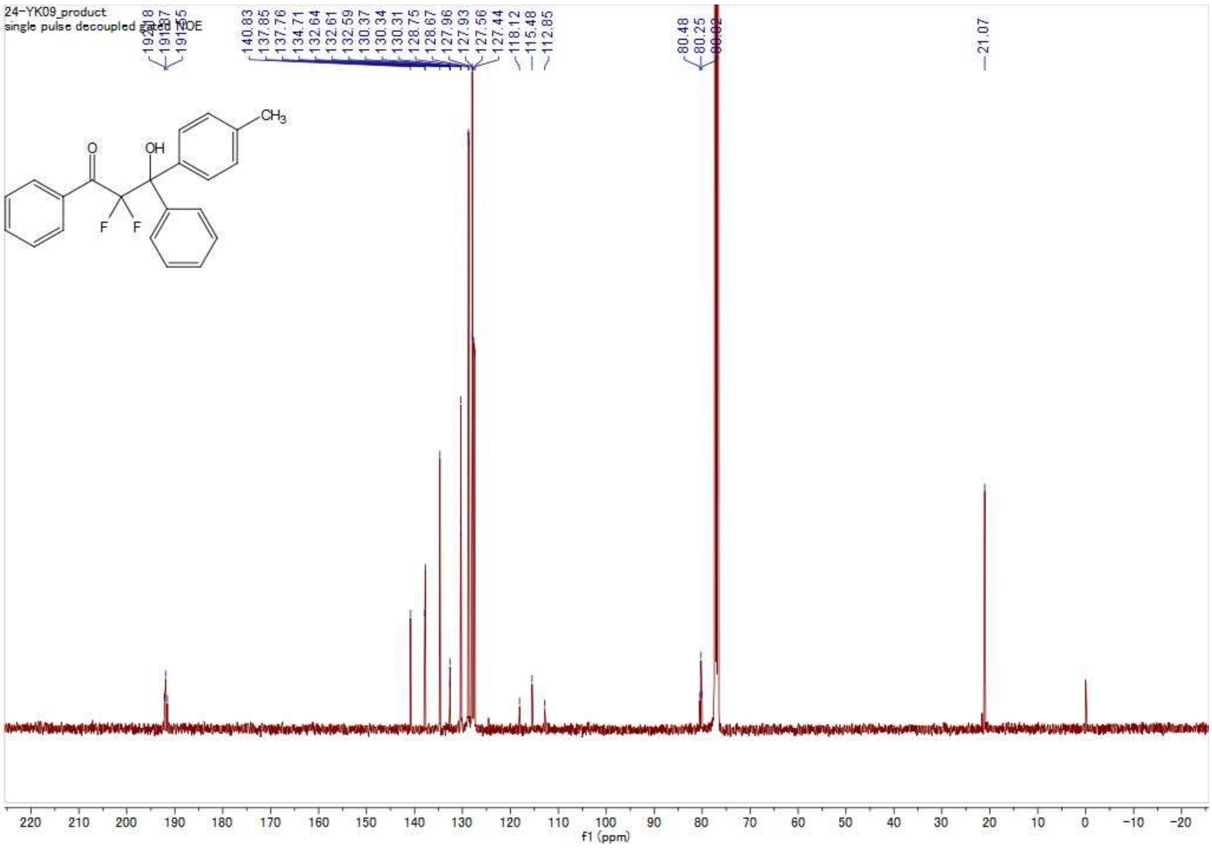

DEPT135 of **6**

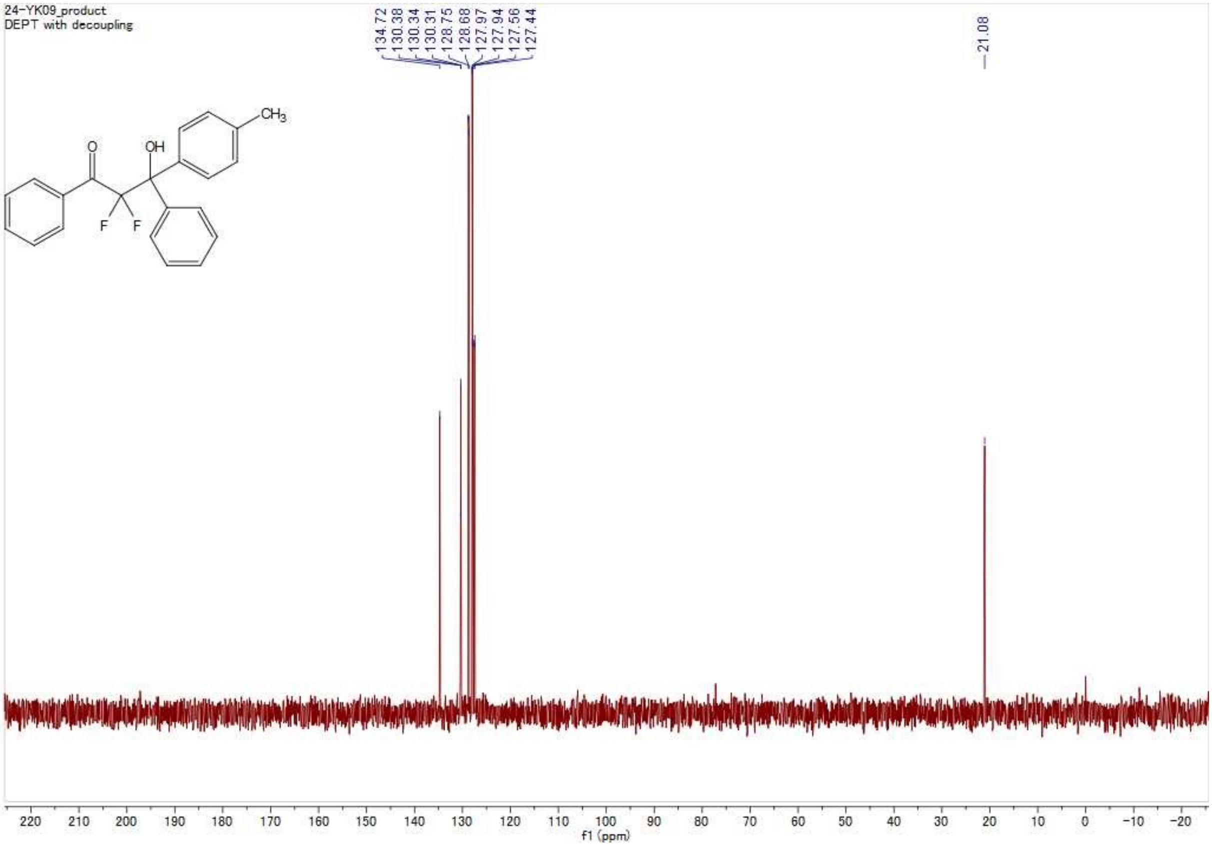

^{1}H NMR of **7**

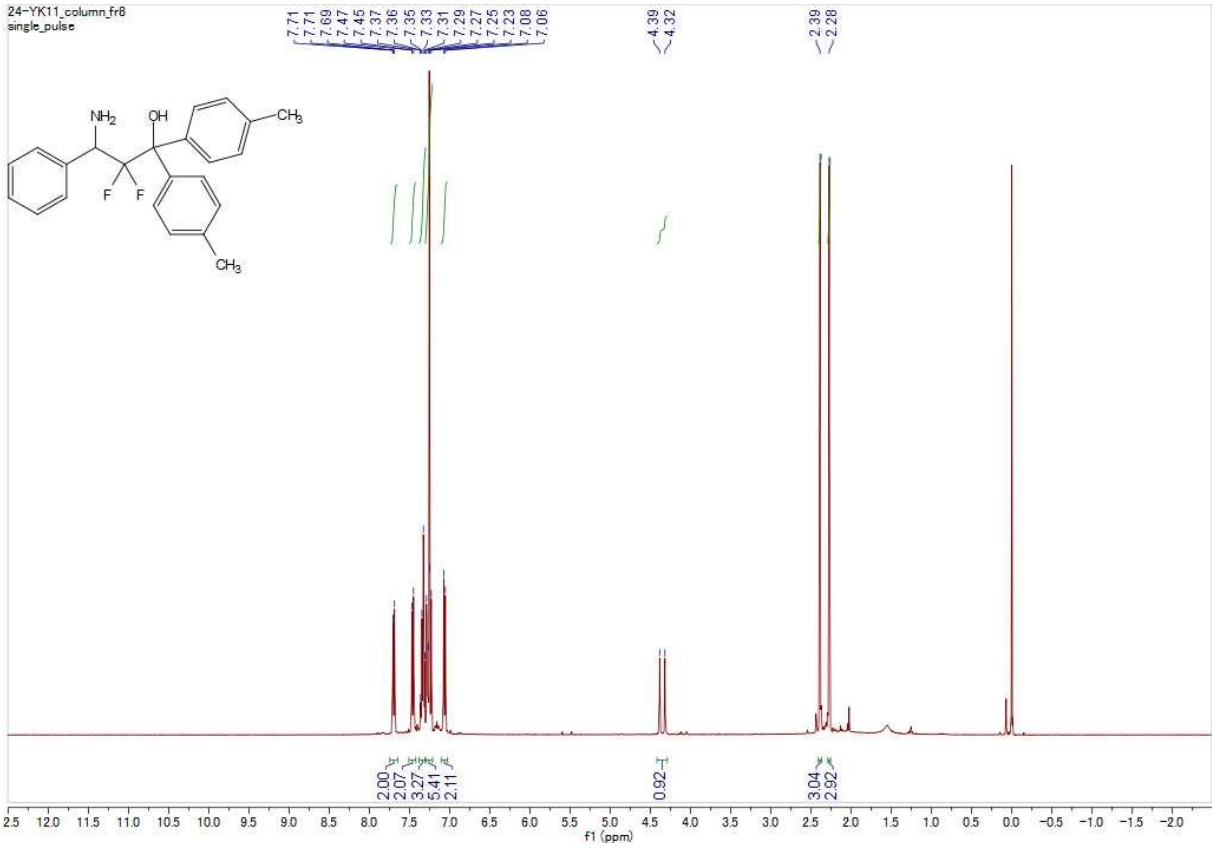

^{19}F NMR of **7**

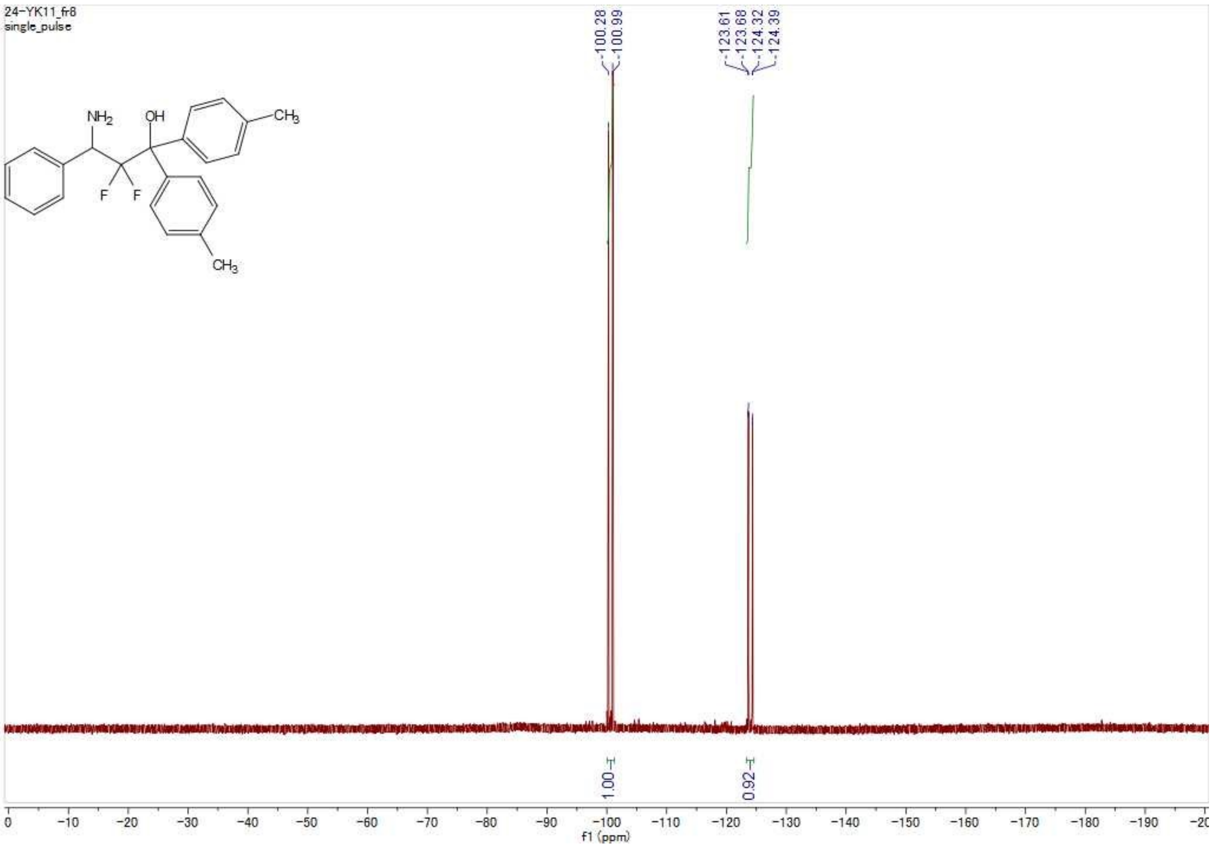

^{13}C NMR of **7**

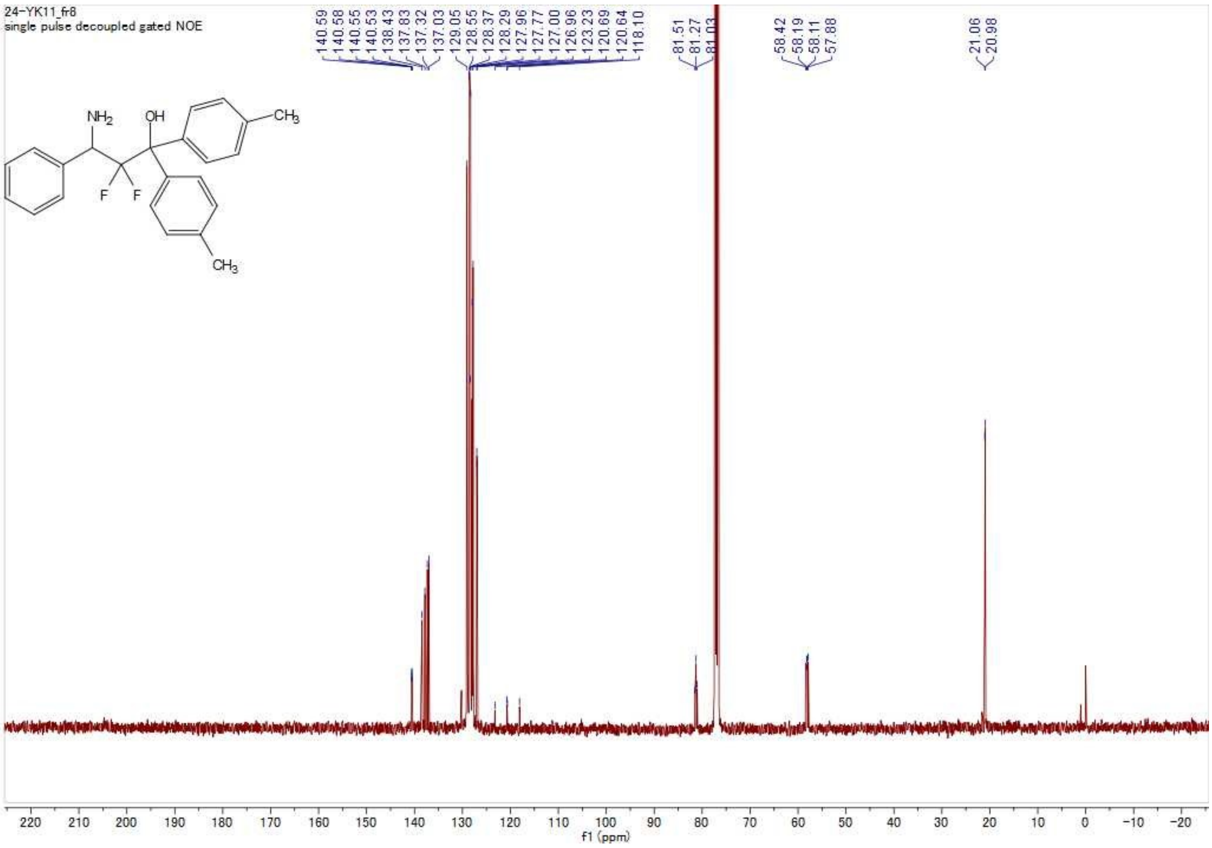

DEPT135 of **7**

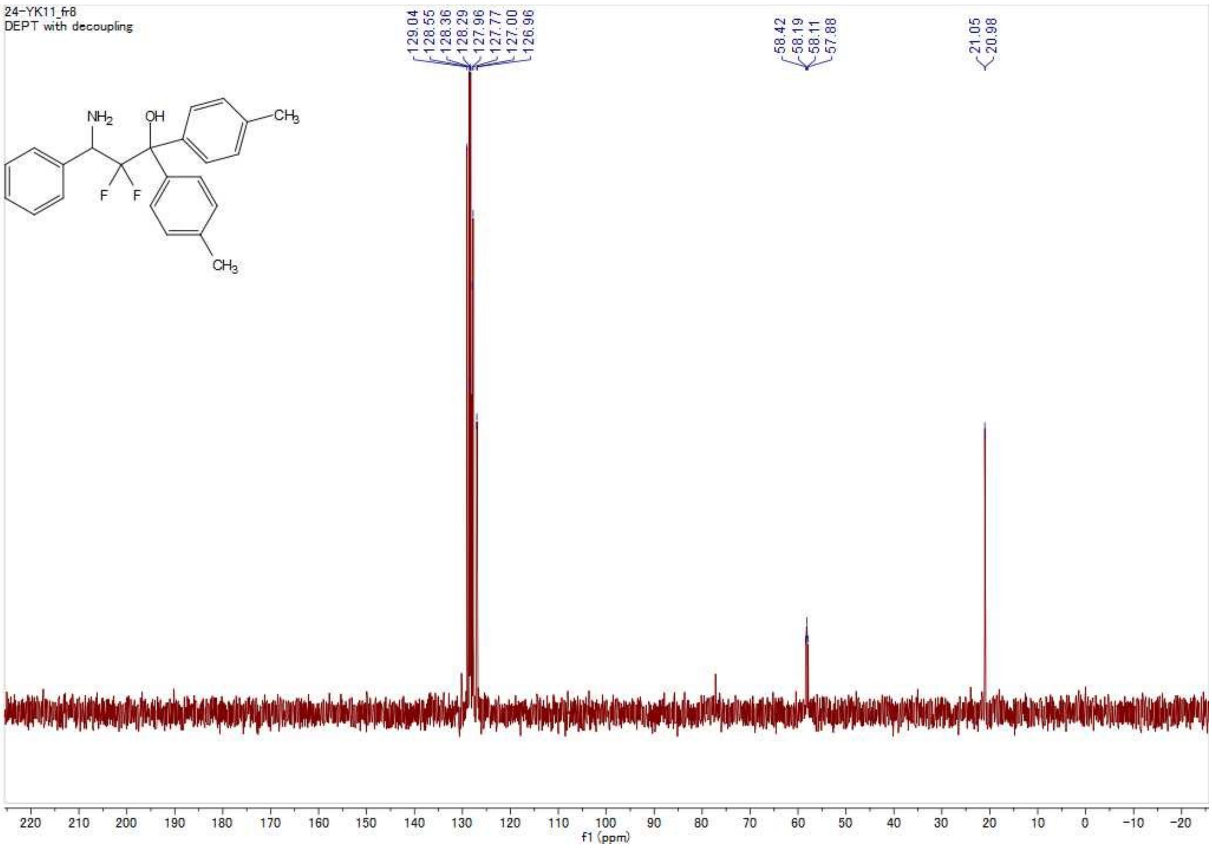

X-ray crystallographic data:

Crystallographic data for the structure have been deposited with the Cambridge Crystallographic Data Centre as supplementary publication CCDC: 1440941-1440946.

Experimental of 5aH.

Single colourless block-shaped crystals of **5aH** were obtained from slow evaporation of ethyl acetate and n-hexane at room temperature. A suitable crystal 0.10×0.08×0.05 mm^3 was selected and mounted on a MiTeGEN Dual Thickness MicroLoops in perfluoropolyether oil on an XtaLAB Synergy R, HyPix diffractometer. The crystal was kept at a steady T = 120.0(4) K during data collection. The structure was solved with the ShelXT 2018/2 (Sheldrick, 2018) structure solution program using the Intrinsic Phasing solution method and by using **Olex2** (Dolomanov et al., 2009) as the graphical interface. The model was refined with version 2018/3 of ShelXL 2018/3 (Sheldrick, 2015) using Least Squares minimisation.

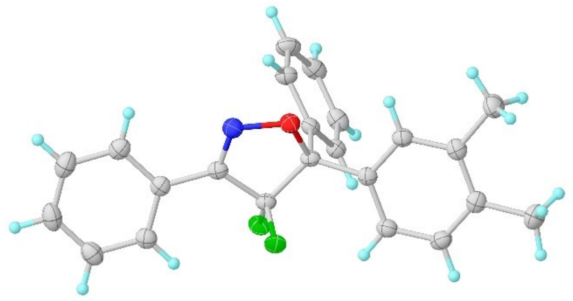

Crystal Data of 5aH.

$C_{23}H_{19}F_2NO$, M_r = 363.39, monoclinic, $P2_1/c$ (No. 14), a = 17.3894(2) Å, b = 6.21370(10) Å, c = 16.7372(2) Å, β = 96.2100(10)°, $\alpha = \gamma$ = 90°, V = 1797.88(4) Å^3, T = 120.0(4) K, Z = 4, Z' = 1, μ(Cu K$_\alpha$) = 0.791, 40254 reflections measured, 3825 unique (R_{int} = 0.0373) which were used in all calculations. The final wR_2 was 0.0912 (all data) and R_1 was 0.0379 (I > 2(I)).

ORTEP drawing of **5aH** showing thermal ellipsoids at the 50% probability level.

Compound 5aH

Formula	$C_{23}H_{19}F_2NO$
$D_{calc.}$/ g cm^{-3}	1.343
μ/mm^{-1}	0.791
Formula Weight	363.39
Colour	colourless
Shape	block
Size/mm^3	0.10×0.08×0.05
T/K	120.0(4)
Crystal System	monoclinic
Space Group	$P2_1/c$
a/Å	17.3894(2)
b/Å	6.21370(10)
c/Å	16.7372(2)
α/°	90
β/°	96.2100(10)
γ/°	90
V/Å^3	1797.88(4)
Z	4
Z'	1
Wavelength/Å	1.54184
Radiation type	Cu K$_\alpha$
Θ_{min}/°	2.556
Θ_{max}/°	78.006
Measured Refl.	40254
Independent Refl.	3825
Reflections with I > 2(I)	3612
R_{int}	0.0373
Parameters	246
Restraints	0
Largest Peak	0.256
Deepest Hole	-0.253
GooF	1.078
wR_2 (all data)	0.0912
wR_2	0.0895
R_1 (all data)	0.0404
R_1	0.0379

Experimental of 5aI.

Single colourless block-shaped crystals of **5aI** were obtained from slow evaporation of ethyl acetate and n-hexane at room temperature. A suitable crystal 0.12×0.08×0.04 mm^3 was selected and mounted on a MiTeGEN Dual Thickness MicroLoops in perfluoropolyether oil on an XtaLAB Synergy R, HyPix diffractometer. The crystal was kept at a steady T = 119.9(5) K during data collection. The structure was solved with the ShelXT (Sheldrick, 2015) structure solution program using the Intrinsic Phasing solution method and by using **Olex2** (Dolomanov et al., 2009) as the graphical interface. The model was refined with version 2018/3 of ShelXL 2018/3 (Sheldrick, 2015) using Least Squares minimisation.

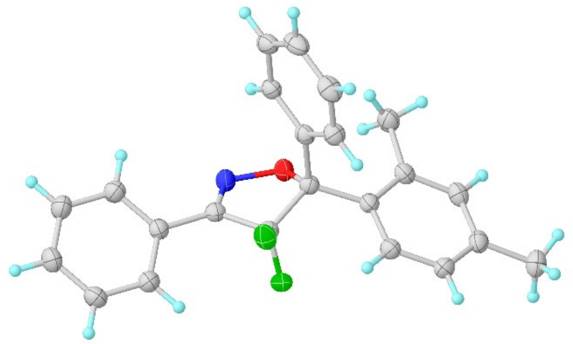

Crystal Data of 5aI.

$C_{23}H_{19}F_2NO$, M_r = 363.39, monoclinic, $P2_1/c$ (No. 14), a = 13.4568(2) Å, b = 16.4098(2) Å, c = 8.25210(10) Å, β = 94.7070(10)°, $\alpha = \gamma = 90°$, V = 1816.11(4) Å^3, T = 119.9(5) K, Z = 4, Z' = 1, μ(Cu K_α) = 0.783, 31987 reflections measured, 3869 unique (R_{int} = 0.0392) which were used in all calculations. The final wR_2 was 0.1079 (all data) and R_1 was 0.0450 (I > 2(I)).

ORTEP drawing of **5aI** showing thermal ellipsoids at the 50% probability level.

Compound 5aI

Formula	$C_{23}H_{19}F_2NO$
$D_{calc.}$/ g cm^{-3}	1.329
μ/mm^{-1}	0.783
Formula Weight	363.39
Colour	colourless
Shape	block
Size/mm^3	0.12×0.08×0.04
T/K	119.9(5)
Crystal System	monoclinic
Space Group	$P2_1/c$
a/Å	13.4568(2)
b/Å	16.4098(2)
c/Å	8.25210(10)
α/°	90
β/°	94.7070(10)
γ/°	90
V/Å^3	1816.11(4)
Z	4
Z'	1
Wavelength/Å	1.54184
Radiation type	Cu K_α
Θ_{min}/°	3.295
Θ_{max}/°	77.725
Measured Refl.	31987
Independent Refl.	3869
Reflections with I > 2(I)	3677
R_{int}	0.0392
Parameters	246
Restraints	0
Largest Peak	0.409
Deepest Hole	-0.169
GooF	1.086
wR_2 (all data)	0.1079
wR_2	0.1061
R_1 (all data)	0.0479
R_1	0.0450

Experimental of 5aJ.

Single colourless block-shaped crystals of **5aJ** were obtained from slow evaporation of ethyl acetate and n-hexane at room temperature. A suitable crystal 0.14×0.07×0.04 mm^3 was selected and mounted on a MiTeGEN Dual Thickness MicroLoops in perfluoropolyether oil on an XtaLAB Synergy R, HyPix diffractometer. The crystal was kept at a steady T = 120.0(5) K during data collection. The structure was solved with the ShelXT (Sheldrick, 2015) structure solution program using the Intrinsic Phasing solution method and by using **Olex2** (Dolomanov et al., 2009) as the graphical interface. The model was refined with version 2018/3 of ShelXL 2018/3 (Sheldrick, 2015) using Least Squares minimisation.

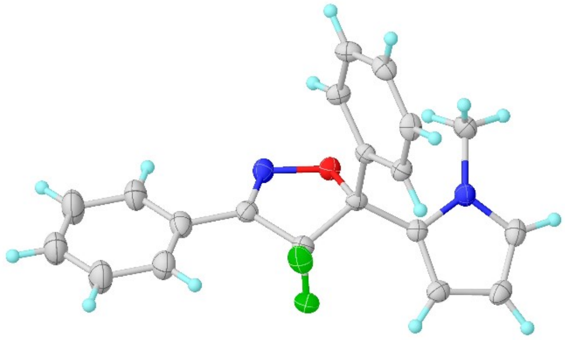

Crystal Data of 5aJ.

$C_{20}H_{16}F_2N_2O$, M_r = 338.35, triclinic, P-1 (No. 2), a = 8.14050(10) Å, b = 12.07940(10) Å, c = 17.1421(3) Å, α = 96.3100(10)°, β = 97.1640(10)°, γ = 93.9910(10)°, V = 1656.39(4) Å^3, T = 120.0(5) K, Z = 4, Z' = 2, μ(Cu K_α) = 0.834, 13056 reflections measured, 13056 unique (R_{int} = .) which were used in all calculations. The final wR_2 was 0.1394 (all data) and R_1 was 0.0486 (I > 2(I)).

ORTEP drawing of **5aJ** showing thermal ellipsoids at the 50% probability level.

Compound 5aJ

Formula	$C_{20}H_{16}F_2N_2O$
$D_{calc.}$/ g cm^{-3}	1.357
μ/mm^{-1}	0.834
Formula Weight	338.35
Colour	colourless
Shape	block
Size/mm^3	0.14×0.07×0.04
T/K	120.0(5)
Crystal System	triclinic
Space Group	P-1
a/Å	8.14050(10)
b/Å	12.07940(10)
c/Å	17.1421(3)
α/°	96.3100(10)
β/°	97.1640(10)
γ/°	93.9910(10)
V/Å^3	1656.39(4)

Z	4
Z'	2
Wavelength/Å	1.54184
Radiation type	Cu K_α
Θ_{min}/°	2.617
Θ_{max}/°	77.956
Measured Refl.	13056
Independent Refl.	13056
Reflections with I > 2(I)	11518
R_{int}	.
Parameters	546
Restraints	169
Largest Peak	0.326
Deepest Hole	-0.316
GooF	1.066
wR_2 (all data)	0.1394
wR_2	0.1357
R_1 (all data)	0.0547
R_1	0.0486
